# ^13^CO_2_ labeling kinetics in maize reveal impaired efficiency of C_4_ photosynthesis under low irradiance

**DOI:** 10.1093/plphys/kiac306

**Published:** 2022-06-25

**Authors:** David B Medeiros, Hirofumi Ishihara, Manuela Guenther, Laise Rosado de Souza, Alisdair R Fernie, Mark Stitt, Stéphanie Arrivault

**Affiliations:** Max Planck Institute of Molecular Plant Physiology, 14476 Potsdam-Golm, Germany; Max Planck Institute of Molecular Plant Physiology, 14476 Potsdam-Golm, Germany; Max Planck Institute of Molecular Plant Physiology, 14476 Potsdam-Golm, Germany; Max Planck Institute of Molecular Plant Physiology, 14476 Potsdam-Golm, Germany; Max Planck Institute of Molecular Plant Physiology, 14476 Potsdam-Golm, Germany; Max Planck Institute of Molecular Plant Physiology, 14476 Potsdam-Golm, Germany; Max Planck Institute of Molecular Plant Physiology, 14476 Potsdam-Golm, Germany

## Abstract

C_4_ photosynthesis allows faster photosynthetic rates and higher water and nitrogen use efficiency than C_3_ photosynthesis, but at the cost of lower quantum yield due to the energy requirement of its biochemical carbon concentration mechanism. It has also been suspected that its operation may be impaired in low irradiance. To investigate fluxes under moderate and low irradiance, maize (*Zea mays*) was grown at 550 µmol photons m^−2^ s^−l^ and ^13^CO_2_ pulse-labeling was performed at growth irradiance or several hours after transfer to 160 µmol photons m^−2^ s^−1^. Analysis by liquid chromatography/tandem mass spectrometry or gas chromatography/mass spectrometry provided information about pool size and labeling kinetics for 32 metabolites and allowed estimation of flux at many steps in C_4_ photosynthesis. The results highlighted several sources of inefficiency in low light. These included excess flux at phospho*enol*pyruvate carboxylase, restriction of decarboxylation by NADP-malic enzyme, and a shift to increased CO_2_ incorporation into aspartate, less effective use of metabolite pools to drive intercellular shuttles, and higher relative and absolute rates of photorespiration. The latter provides evidence for a lower bundle sheath CO_2_ concentration in low irradiance, implying that operation of the CO_2_ concentration mechanism is impaired in this condition. The analyses also revealed rapid exchange of carbon between the Calvin–Benson cycle and the CO_2_-concentration shuttle, which allows rapid adjustment of the balance between CO_2_ concentration and assimilation, and accumulation of large amounts of photorespiratory intermediates in low light that provides a major carbon reservoir to build up C_4_ metabolite pools when irradiance increases.

## Teaser

Analysis of metabolite pools, sizes, and fluxes reveals that multiple interlocking factors decrease the efficiency of photosynthesis in low irradiance in maize.

## Introduction

After the appearance of oxygenic photosynthesis about 2.7 billion years ago, falling atmospheric CO_2_ and rising O_2_ exposed a side reaction of Rubisco with O_2_ that leads to formation of 2-phosphoglycolate (2PG) (Lorimer, 1981; Bauwe et al., 2010). 2PG is recycled at the cost of energy, CO_2_, and NH_3_ in a process termed photorespiration (Bauwe et al., 2010; Bauwe, 2018). In C_4_ photosynthesis, a biochemical carbon concentration mechanism (CCM) generates an elevated CO_2_ concentration in enlarged bundle sheath cells (BSCs) around the vasculature, where Rubisco and the rest of the Calvin–Benson cycle (CBC) is located (von Caemmerer and Furbank, 2003; Langdale, 2011; Sage, 2017; Schlüter and Weber, 2020). The CCM starts with assimilation of bicarbonate by phospho*enol*pyruvate carboxylase (PEPC) in the mesophyll cells (MCs). 4-Carbon metabolites diffuse to the BSC where they are decarboxylated to release CO_2_ and 3-carbon metabolites that move back to the MC (Hatch, 2002; von Caemmerer and Furbank, 2003; Sage et al., 2011; Sage, 2017). C_4_ photosynthesis emerged 25–30 million years ago in response to a transition in the Earth’s climate from hot and wet conditions with atmospheric CO_2_ concentrations >1000 ppm to cooler and drier conditions with CO_2_ concentrations <300 ppm (Christin et al., 2008; Zachos et al., 2008). C_4_ photosynthesis evolved independently in at least 65 lineages (Sage et al., 2011; Sage, 2017) and, even after emerging in a given lineage, it continued to evolve and diversify (Bianconi et al., 2020). C_4_ species represent ∼3% of terrestrial plant species, account for 23% of total terrestrial carbon (C) gain (Still et al., 2003; Sage et al., 2011; Sage, 2017) and are prevalent in hot or dry environments in which photorespiration would be especially high (Still et al., 2003; Christin and Osborne, 2014; Sage, 2017; Sage et al., 2018). C_4_ photosynthesis brings several advantages including faster photosynthesis, lower photorespiration (Osmond and Harris, 1971; Sage et al., 2011), increased water use efficiency (Ghannoum et al., 2011), and increased nitrogen use efficiency (Brown, 1978; Kapralov et al., 2011; Sharwood et al., 2016; Iniguez et al., 2020). These gains, however, are at the cost of using part of the light energy to drive the CCM (Zhu et al., 2008). The resulting decrease in quantum efficiency will have an especially large impact in low irradiance. There is also considerable interest in whether further factors decrease the efficiency of C_4_ photosynthesis in low irradiance (Bellasio and Griffiths, 2014a, 2014b; Kromdijk et al., 2014; Sage, 2014). Any loss of photosynthetic efficiency in low light (LL) would impact on the yield of C_4_ crop plants growing in dense canopies.

The pathway of C_4_ photosynthesis is well established, with the exception of some transport steps (Weber and von Caemmerer 2010; Bräutigam et al., 2014; Schlüter and Weber, 2020). Independent evolution of C_4_ photosynthesis in different lineages has resulted in the use of three different decarboxylation reactions: plastidic NADP-dependent malic enzyme (NADP-ME); mitochondrial NAD-dependent malic enzyme (NAD-ME); or cytosolic PEP carboxykinase (PEPCK). There are accompanying differences in which 4-carbon metabolite moves to the BSC and which 3-carbon metabolite returns to the MC, as well as in the energy demand and where energy is required (Hatch, 2002; Heldt et al., 2005; Bräutigam et al., 2018; see Supplemental Text S1). There are many open questions related to the operation of C_4_ photosynthesis, and especially to how it may operate in different conditions (see Kromdijk et al*.*, 2010) including low irradiance.

C_4_ photosynthesis requires rapid intercellular movement of metabolites. This occurs largely by diffusion, driven by large intercellular concentration gradients (Leegood 1985; Stitt and Heldt, 1985a) and depends on efficient use of metabolite pools to generate these gradients. C_4_ photosynthesis also depends on the efficiency with which the decarboxylation reaction can operate to generate a high CO_2_ concentration in the BSC (C_BSC_). For example, rapid decarboxylation by NADP-ME or NAD-ME depends on re-oxidation of NADPH in the plastid or NADH in the mitochondria, respectively (Bräutigam et al., 2018, Zhao et al., 2022). Our current understanding of how concentration gradients are generated and decarboxylases operate is mainly based on measurements of total metabolite contents. However, in many cases, part or much of the total content is not directly involved in photosynthesis (see Arrivault et al., 2017, also discussion in Stitt and Heldt, 1985a; Usuda, 1987; Doncaster et al., 1989; Allen and Young, 2020). There is a need for more comprehensive information about the sizes of pools that are directly involved in photosynthesis, and about fluxes through these pools, as well as whether the size of these pools and the generated concentration gradients depend on the conditions, including low irradiance.

Although species that use different decarboxylation types are sometimes classified into distinct subtypes, many C_4_ species operate more than one route concomitantly (Furbank, 2011; Bräutigam et al., 2014; Wang et al., 2014; Weissmann et al., 2016; Arp et al., 2021). Indeed, Wang et al. (2014) argued that a CCM with only PEPCK is unlikely because it would require more energy in the BSC than could be delivered by BSC chloroplasts. It has been proposed that simultaneous operation of more than one pathway might decrease the size of the concentration gradients that is required to drive intercellular diffusion of a given metabolite (Furbank, 2011) or increase flexibility under different environmental conditions (Bellasio and Griffiths, 2014b; Stitt and Zhu, 2014; Wang et al., 2014). For example, measurements of overall metabolite levels in the NADP-ME subtype maize (*Zea mays*) indicate that the contribution of other decarboxylases might increase in low irradiance (Usuda, 1987; Leegood and von Caemmerer, 1989). Based on textbook pathways, the energy requirement is lower for the PEPCK than the NADP-ME route, but this has recently been called into question (see Supplemental Text S1). There is a need for analyses of flux to determine if and under what conditions decarboxylation pathways operate in parallel. This would provide a firmer basis for hypotheses about what advantage or disadvantage they might bring.

Another longstanding question relates to how flux is balanced between the CCM and CBC (Furbank and Hatch, 1987; Jenkins et al., 1989; Furbank et al., 1990; von Caemmerer, 2000; Kromdijk et al., 2014). Insufficient CCM flux would result in a low C_BSC_ and increased rates of Ribulose-1,5-bisphosphate (RuBP) oxygenation and photorespiration, whereas excess CCM flux would result in a high C_BSC_ and increased CO_2_ back-leakage to the MC (von Caemmerer, 2000; Kromdijk et al., 2014). Balancing of flux in the CCM and CBC will require regulation of enzyme activities (Doncaster and Leegood 1987; Doncaster et al., 1989; Furbank et al., 1997, Matsuoka et al., 2001). It will also require poising of metabolite levels, both to maintain substrates at appropriate levels for enzyme activity in the CCM and CBC and to generate the concentration gradients that drive intercellular shuttles (see above). The levels of most metabolites involved in C_4_ photosynthesis rise with increasing irradiance (Usuda, 1987; Leegood and von Caemmerer 1988, 1989; Doncaster et al., 1989; Ubierna et al., 2013). During a dark–light transition, the levels of CCM and CBC metabolites undergo large transient changes, and total C in these pools rises faster than can be accounted for by de novo C fixation, suggesting that they are in part built up by movement of C between existing pools of metabolites (Leegood and Furbank 1984; Usuda, 1985). However, little is known about how the CBC and CCM pools are balanced under different irradiance, and how quickly C can be shifted between the CBC and the CCM during photosynthesis.

Whereas it is clear that C_4_ plants carry out photorespiration (Osmond and Harris, 1971; Furbank and Badger, 1982), open questions remain about its role. Enzymes and transcripts for the photorespiratory pathway are present in C_4_ species, although at lower levels than in C_3_ species (Ohnishi and Kanai, 1983; Ueno et al., 2005; Mallmann et al., 2014), and mutations in the pathway are lethal (Zelitch et al., 2009). Based mainly on the response to a decrease in O_2_, photorespiration has been estimated to be 2%–7% of the rate of CO_2_ assimilation in C_4_ plants (Volk and Jackson, 1972; de Veau and Burris, 1989; Laisk and Edwards, 1998) with possibly higher rates after transfer to LL (Henderson et al., 1992; Kromdijk et al., 2010). The latter may be a consequence of a transient decrease in the C_BSC_ due to impaired operation of the CCM (Ubierna et al., 2011; Bellasio and Griffiths, 2014b). In C_3_ photosynthesis, photorespiration plays an essential role in recycling 2PG, but has also acquired ancillary functions (Bauwe et al., 2010; Bauwe, 2018). In C_4_ photosynthesis, photorespiratory intermediates might provide a C reservoir to allow build-up of CBC and CCM metabolite pools (Bartsch et al., 2010). However, information about rate of photorespiration in C_4_ photosynthesis and the size of photorespiratory intermediate pools in different conditions is needed to test these ideas.

There are also open questions related to CO_2_ back-leakage. Back-leakage is decreased by suberization of the BSC and by positioning of organelles to improve CO_2_ recapture (von Caemmerer and Furbank, 2003; Danila et al., 2021). It nevertheless occurs (Furbank et al., 1989, 1990) and is probably unavoidable due to the high density of plasmodesmata (Weiner et al., 1988; Sowinski et al., 2008; Danila et al., 2016, 2018) that is required for intercellular metabolite shuttling. The rate of back-leakage is often expressed as leakiness (Φ), the ratio of CO_2_ back-leakage from the BSC to the MC relative to the rate at which PEPC assimilates bicarbonate (von Caemmerer, 2000; Kromdijk et al., 2014). As Φ cannot be measured it is estimated using various indirect approaches (Ubierna et al., 2011; Kromdijk et al., 2014; Sage, 2014). Estimated values are usually in the range of 0.2–0.3 (Hatch et al., 1995; Kromdijk et al., 2014) but depend on the conditions, for example, back-leakage may increase in low irradiance (Evans et al., 1986; Kromdijk et al., 2014), and can be as high as 0.6–0.9 after a sudden decrease in irradiance (Cousins et al., 2006, 2008; Tazoe et al., 2006, 2008; Kromdijk et al. 2008, 2010, 2014; Pengelly et al. 2010). High Φ in low irradiance may be partly due to CO_2_ released by photorespiration or mitochondrial respiration making a larger relative contribution to C_BSC_ (Kromdijk et al., 2010, 2014; Bellasio and Griffiths, 2014a). On the other hand, Φ does not necessarily increase when plants are grown in LL (Tazoe et al., 2008; Kromdijk et al., 2010; Pengelly et al., 2010; Bellasio and Griffiths, 2014a). However, estimation of Φ requires many assumptions. An analysis of metabolic fluxes might provide independent insights into the magnitude of back-leakage in different conditions.

The analysis of ^14^CO_2_ labeling kinetics played a key role in the discovery of C_4_ photosynthesis (Hatch and Slack, 1966; Nickell, 1993; Hatch, 2002) but since then there have been few detailed analyses of fluxes in C_4_ photosynthesis. In the last decade methods were established that combine labeling with the stable isotope ^13^CO_2_ and liquid chromatography and tandem mass spectrometry (LC–MS/MS) or gas chromatography linked to mass spectrometry (GC–MS). These allow labeling kinetics to be analyzed for large sets of metabolites (Szecowka et al., 2013; Ma et al., 2014; Allen and Young, 2020, Xu et al., 2021, 2022). Their application to C_4_ photosynthesis revealed that flux is shifted from a NADP-ME decarboxylation route to a PEPCK route in maize *dct1* mutants, which are impaired in a transporter that imports malate into the BSC chloroplast (Weissmann et al., 2016). Studies in wild-type maize (Arrivault et al., 2017) confirmed that overall labeling kinetics were fully consistent with the topology of the C_4_ pathway, and provided direct evidence for the presence of substantial intercellular concentration gradients for 3-phosphoglycerate (3PGA), triose-phosphate (triose-P), malate, aspartate, probably alanine but not for pyruvate. They also indicated that PEPCK or NAD-ME is responsible for a minor component of the CCM, demonstrated that there is quite rapid C exchange between the CBC and the CCM, and provided direct evidence for flux through photorespiration.

As outlined above, there are several reasons to suspect that low irradiance may decrease the efficiency of C_4_ photosynthesis. In low irradiance there will be a particularly strong tradeoff between the use of light energy to drive CO_2_ concentration and to drive CO_2_ fixation in the CBC. It may also be especially challenging to coordinate fluxes in two pathways and across two cell types, each of which has its own light reactions (Leegood and von Caemmerer, 1989; Bellasio and Griffiths, 2014a, 2014b; Kromdijk et al., 2014; Sage, 2014). In the following experiments, we compare pool sizes and flux patterns in maize in moderate and low irradiance and show that several interlocking factors impair photosynthetic efficiency of maize in low irradiance.

## Results

### Experimental design

Comparison of photosynthetic flux in different conditions requires analysis of labeling kinetics in short and precise ^13^CO_2_ pulses. We redesigned the labeling set-up used in Arrivault et al. (2017) to ensure better reproducibility of short pulses (Supplemental Figure S1; see “Materials and Methods”). One major upgrade was a gas mixing system in which separate air mixtures with ^12^CO_2_ and ^13^CO_2_ were continuously run through a four-way valve, with the former initially directed to the labeling chamber and the latter to a CO_2_ trap. This allowed almost instantaneous switching from the ^12^CO_2_ to the ^13^CO_2_ air mixture. A second major upgrade was a custom-designed labeling chamber with a gas half-time of 0.35 s (calculated as described in Szecowka et al., 2013). Plant material was quenched without opening the chamber by pouring liquid N_2_ through a plastic funnel in the chamber lid. This set-up allowed reliable and reproducible ^13^CO_2_ pulses as short as 5 s (see also Ermakova et al., 2021).

Maize (*Z.mays*) plants were grown at an irradiance of 550 µmol m^−2^ s^−1^ (medium light [ML]). On the day of the experiment, they were left at ML or illuminated from dawn on with 160 µmol m^−2^ s^−1^ (LL). The rate of photosynthesis in ML and LL was 22.4 ± 1.5 and 8.3 ± 0.7 µmol CO_2_ m^−2^ s^−1^, respectively, or 122.8 ± 8.2 and 45.5 ± 3.9 nmol CO_2_ g^−1^ FW s^−1^ (specific leaf area of 0.005484 m^−2^ g^−1^). Pulse duration was chosen to match the slower flow of ^13^C through metabolism in LL, and ranged between 5 s and 60 min in ML, and 10 s and 240 min in LL. We collected two to six biological replicates for each pulse duration. Labeling was performed 5–12 h after the start of the light period in ML and 3–13 h after the start of the light period in LL. In both cases, samples were collected on 4 consecutive days. Samples with different pulse durations were collected in a randomized manner with respect to day sampled and time of day. Metabolites were analyzed by LC–MS/MS and GC–MS, providing coverage of 32 metabolites (see Supplemental Table S1 for a list of analyzed metabolites, abbreviations, their assignment to metabolic sectors, and the method of analysis). Original data for isotopomer abundance, metabolite content (calculated by summing all isotopomers for a given metabolite), and ^13^C enrichment are provided in Supplemental Data Sets S1–S6. Some variation was seen in metabolic contents between samples (Supplemental Data Set S5) but for most metabolites this was unrelated to the day and time of sampling (Supplemental Figure S2), indicating that metabolism was in steady state. Exceptions included glucose and fructose in LL whose levels rose significantly later in the light period and aspartate in LL whose level showed a downward trend later in the light period.

### Correction for inactive pools and deconvolution of positional labeling in malate and aspartate

We initially performed *k*-means clustering on ^13^C enrichment calculated from raw isotopomer data. Whereas enrichment kinetics largely followed the pattern expected for C_4_ photosynthesis (Supplemental Figure S3), there were deviations, as previously seen in Arrivault et al. (2017). In particular, malate and sedoheptulose 1,7-bisphosphate (SBP) are expected to label rapidly (malate is a 4-carbon acid involved in shuttling of newly fixed C from the MC to the BSC, SBP is a CBC intermediate) but were assigned to slow-labeling clusters. These deviations can be attributed to two factors: (1) compartmentalized metabolite pools that are not involved in photosynthesis and (2) the topology of C_4_ photosynthesis (Arrivault et al., 2017). The data were processed to correct for these confounding factors.

For most metabolites that labeled rapidly, the unlabeled isotopomer (*m*_0_) declined to a low level. However, for some, *m*_0_ showed a rapid initial decline and then plateaued, whereas heavily labeled isotopomers rose to represent a high proportion of the other isotopomers (Supplemental Figure S4; these plots use a linear *x*-axis for better visualization of the plateau). In many cases, this pattern points to the existence of at least two pools, one involved in photosynthesis and labeled to high enrichment by newly fixed ^13^C, and another that is not involved in photosynthesis and is not rapidly labeled by newly fixed ^13^C (termed “active” and “inactive” pools, respectively). Metabolites with a large inactive pool included malate, SBP, glucose 1-phosphate (G1P), UDP-glucose (UDPG), serine, glycine, fumarate, and (in ML only) glycerate. The inactive pool (estimated as the average *m*_0_ value in the plateau) was subtracted from the *m_o_* value in each sample to obtain the corrected *m_o_* value for the active pool (calculation steps for correction are presented in Supplemental Data Sets S1 and S3). This corrected *m_o_* value was combined with values for the labeled isotopomers to estimate the size (nmol g^−1^ FW) and enrichment (%) of the active pool (Supplemental Tables S2 and S3, respectively). We used these corrected values for all subsequent calculations and data displays.

Overall enrichment of aspartate and malate (even after correcting for the inactive pool) rose rather slowly (Supplemental Table S3). This is because ^13^C is rapidly incorporated by PEPC onto the C4 position of 4-carbon acids, but only slowly into positions C1, C2, and C3 (hereafter referred to as C1–3 or the “backbone”). Labeling of C1–3 requires that ^13^C is fixed in the CBC and then moves from 3PGA via phosphoglycerate mutase and enolase into Phospho*enol*pyruvate (PEP), and from PEP into the backbone of the 4-carbon acids. Our mass spectrometry data do not give direct information about positional labeling. Instead, we used the known pathway topology of C_4_ photosynthesis to deconvolute the positional labeling of malate and aspartate. During a ^13^CO_2_ pulse, every molecule of oxaloacetate (OAA) that is formed by PEPC will contain a ^13^C atom in its C4 position, irrespective of whether PEP is labeled or not. Thus, every molecule of malate or aspartate generated downstream of PEP must include a ^13^C at its C4 position, irrespective of whether it is an *m_1_*, *m_2_*, *m_3_*, or *m_4_* isotopomer. In order to estimate position-dependent enrichments in the C4 and C1–3 positions of malate and aspartate, we first estimated ^13^C amounts (natom ^13^C equivalents g^−1^ FW) in malate and aspartate (calculation steps are presented in Supplemental Data Sets S7 and S8). Estimations of ^13^C amounts were carried out in two ways; either using the average metabolite pool size, or by using metabolite pool size at each time point. Both approaches yielded similar results (Supplemental Figure S5). Estimates from the first approach were used for all further calculations and displays.

**Figure 1:**
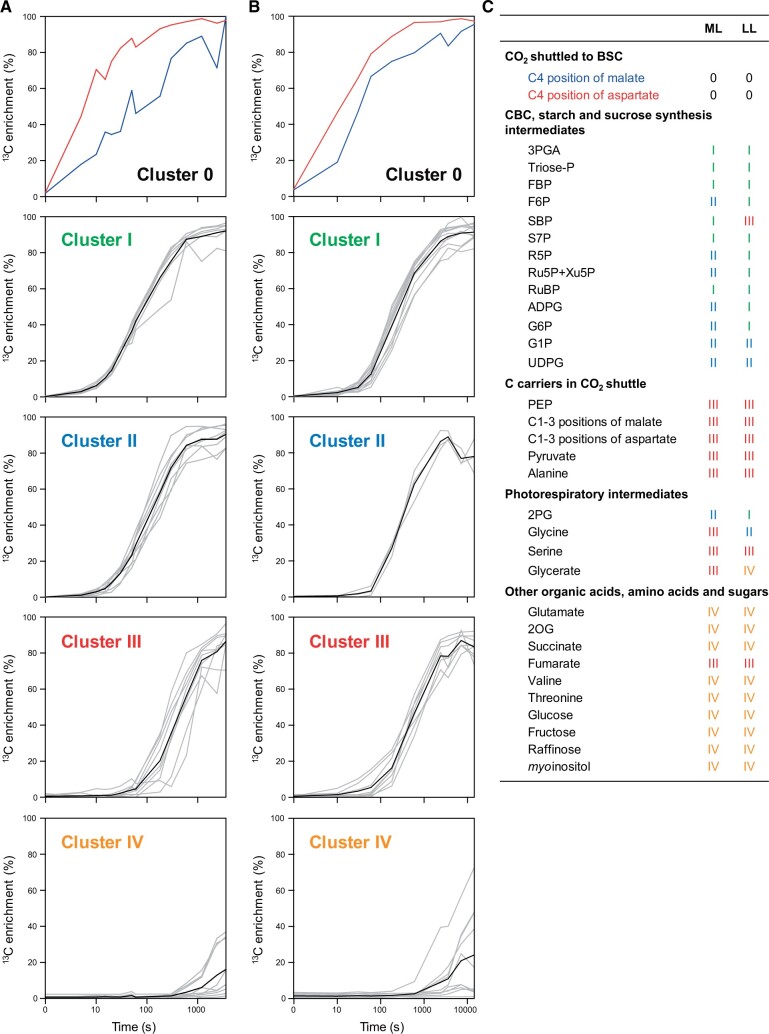
Overview of corrected ^13^C enrichment kinetics by *k*-means clustering. A, Clustering in medium light (ML), and B, in low light (LL) using data corrected for inactive pools and positional labeling in malate and aspartate. Carbon position-dependent ^13^C enrichments were separately calculated for the C4 and C1–3 positions of malate and aspartate (for further information about calculations, see Supplemental Data Set S9). Data are presented in Supplemental Table S3. In clusters I–IV, gray lines show the ^13^C enrichment of individual metabolites and black lines show average ^13^C enrichment of all metabolites in the cluster. The *x*-axis corresponds to the labeling time on a log scale. The duration of the applied pulses was extended to match the expected slower flow of ^13^C through metabolism in LL. Therefore, the scale of the *x*-axis is adjusted such that the *x*-axis is four-fold more expanded in LL (B) than in ML (A) plots. C, Metabolites grouped according to the process in which they are primarily involved and summary of their cluster grouping in ML and LL. Abbreviations listed in alphabetical order: 2PG, 2-phosphoglycolate; 2OG, 2-oxoglutarate, 3PGA, 3-phosphoglycerate; ADPG, ADP-glucose; BSC, bundle sheath cells; CBC, Calvin-Benson cycle; F6P, fructose 6-phosphate; FBP, fructose 1,6-bisphosphate; G1P, glucose 1-phosphate; G6P, glucose 6-phosphate; PEP, phosphoenolpyruvate; R5P, ribose 5-phosphate; Ru5P+Xu5P, ribulose 5-phosphate + xylulose 5-phosphate; RuBP, ribulose 1,5-bisphosphate; S7P, sedoheptulose 7-phosphate; SBP, sedoheptulose 1,7-bisphosphate; Triose-P, triose-phosphate (dihydroxyacetone phosphate); UDPG, UDP-glucose.

Figure 1 shows the results of *k*-means clustering using the corrected data set with ^13^C enrichment values for active metabolite pools and with separate estimates of ^13^C enrichment in the C4 and C1–3 positions of malate and aspartate (for data, see Supplemental Table S3; for plots of ^13^C enrichment for each individual metabolite, see Supplemental Figure S6). The scale of the time axes was expanded in the LL plot, to take account of the lower rate of photosynthesis in LL. The next subsection focuses on shared features in ML and LL. Differences between ML and LL will be dealt with in later sections.

### General features that are shared in ML and LL

The clusters recapitulated the topology of C_4_ photosynthesis, both in ML and LL. Cluster 0 labeled most rapidly and contained the C4 positions of malate and aspartate, corresponding to ^13^C that has been incorporated by PEPC and is en route to the BSC where it is released. Enrichment rose faster in aspartate than in malate (see later for more analysis). Cluster I labeled slightly more slowly and included most of the CBC intermediates plus (in LL) ADP-glucose (ADPG). At the end of the kinetic, their ^13^C enrichment was >90% (Supplemental Table S3; Supplemental Figure S6; Supplemental Data Sets S2 and S4). Incomplete labeling of CBC intermediates was previously seen in maize (Weissmann et al., 2016; Arrivault et al., 2017) and several C_3_ species (Hasunuma et al*.*, 2009; Szecowka et al., 2013; Ma et al., 2014; Arrivault et al., 2017; Allen and Young, 2020; Xu et al., 2021, 2022; Wieloch et al., 2022) and may be due to some backflow of unlabeled C from large and slowly turning over pools like sucrose and starch (Xu et al., 2021). Cluster II labeled more slowly. It contained intermediates involved in starch and sucrose synthesis like glucose 6-phosphate (G6P), G1P, and UDPG. These intermediates have a relatively small stromal pool and a larger cytosolic pool, which are labeled more slowly because flux in the sucrose biosynthesis pathway is lower than in the CBC (see Stitt and Heldt, 1985b; Szecowka et al., 2013). Cytosolic pools might also explain why fructose 6-phosphate (F6P), ribose 5-phosphate (R5P), and ribulose 5-phosphate + xylulose 5-phosphate (Ru5P + Xu5P) are in cluster II in ML. Cluster III labeled after an initial delay and included PEP, the C1–3 positions of malate and aspartate, as well as pyruvate and alanine. These represent the 3-carbon backbone on which C is shuttled into the BSC. PEP and 3PGA might be expected to be at or close to isotopic equilibrium, as they are linked via reversible reactions catalyzed by phosphoglycerate mutase and enolase. As discussed in Arrivault et al. (2017), delayed labeling of PEP compared to 3PGA may be due to recycling of unlabeled C from intermediates in the CO_2_ shuttle. The PEPC reaction does not incorporate any ^13^C into the C1–3 positions of malate and aspartate and, following decarboxylation, these unlabeled carbons are recycled to PEP, strongly diluting the ^13^C that enters PEP from the CBC during the first part of the labeling kinetic. Photorespiration intermediates were distributed between cluster I (2PG in LL), cluster II (2PG in ML; glycine in LL), cluster III (glycine, serine, and glycerate in ML; serine in LL), and cluster IV (glycerate in LL). Other organic acids, amino acids, and sugars labeled slowly and were assigned to cluster III (e.g. fumarate) or cluster IV (e.g. 2-oxoglutarate [2OG], glutamate, succinate).

### Differences in metabolite pool sizes between ML and LL

**Figure 2:**
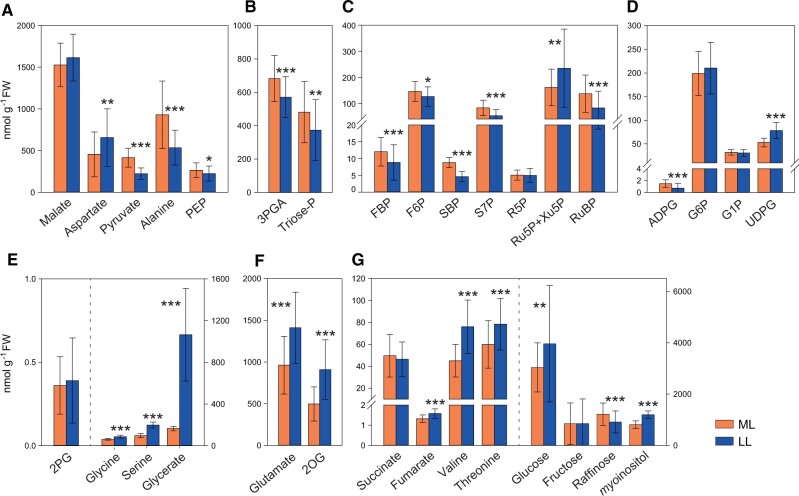
Metabolite active pool sizes in ML and LL. For a given compound, amounts of all isotopomers were summed. Total amounts of pyruvate, PEP, and 3PGA were determined enzymatically. Metabolites are grouped according to the process in which they are primarily involved: A, CO_2_ shuttle intermediates; B, 3PGA and triose-P; C, other CBC intermediates; D, starch and sucrose intermediates; E, photorespiratory intermediates; F, glutamate and 2OG; and G, further organic acids, amino acids and sugars. Amounts (nmol g^−1^ FW) are shown for medium light (ML) and for low light (LL). Mean ± sd, *n* = 50–60 and 43–45 replicates in ML and LL, respectively. Significant differences between the two irradiances according to Student’s *t* test are indicated by asterisks (*P < 0.05, **P < 0.01 and ***P < 0.001). The amounts of the unlabeled form and each ^13^C-isotopomer are provided in Supplemental Data Sets S1 and S3, the total amounts in Supplemental Data Set S5, and mean ± sd in Supplemental Table S2. For abbreviations, see legend of Figure 1.

**Figure 3:**
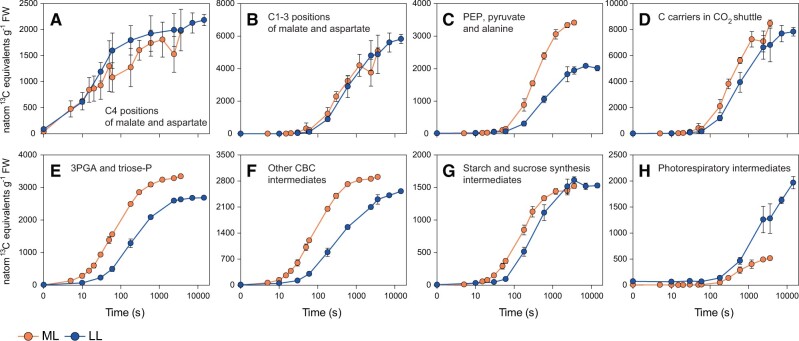
Summed ^13^C amounts in the C4 positions of malate and aspartate and the C1–3 positions of malate and aspartate, compared to summed ^13^C amounts in other sets of metabolites. A, C4 positions of malate and aspartate; B, C1–3 positions of malate and aspartate; C, PEP, pyruvate, and alanine; D, C1–3 positions of malate and aspartate, PEP, pyruvate and alanine; E, 3PGA and Triose-P; F, other CBC intermediates; G, starch and sucrose intermediates (G6P, G1P, UDPG, ADPG); and H, photorespiratory intermediates. ^13^C amounts (natoms ^13^C equivalents g^−1^ FW) are shown for medium light (ML) and for low light (LL). Mean ± sd, *n* = 3–6 and 2–6 replicates in ML and LL, respectively. For information about calculations, see Supplemental Data Sets S7 and S8. Means ± sd are presented in Supplemental Table S5. For abbreviations, see legend of Figure 1.

We next compared ML and LL, starting by inspecting pool sizes. Active pool sizes of individual metabolites are summarized in Figure 2 (see Supplemental Table S2 for values). The summed ^13^C in sets of metabolites that are involved in various sub-processes are shown in Figure 3 (individual metabolite amounts and summed amounts in Supplemental Tables S4 and S5, respectively; see Supplemental Figure S7 for the labeling kinetics from which amounts were estimated). They include the amount of ^13^C en route to the BSC (Figure 3A; the C4 positions of malate and aspartate), in the backbone that carries C into the BSC (Figure 3B, C1–3 positions of malate and aspartate), in the 3-carbon metabolites that move back to the MC (Figure 3C; PEP, pyruvate, and alanine), in the backbone of all CO_2_ shuttle metabolites (Figure 3D; PEP, C1–3 positions of malate and aspartate, and pyruvate and alanine), in the energy shuttle (Figure 3E; 3PGA and triose-P), in other CBC metabolites (Figure 3F), in metabolites of starch and sucrose biosynthesis (Figure 3G) and in photorespiratory metabolites (Figure 3H).

Most metabolite pools were larger in ML than LL. This will, via mass action alone, increase the rate of enzyme catalysis and, by providing larger total pools from which concentration gradients can be generated, support faster intercellular diffusion of metabolites. Metabolites that increased significantly in ML compared to LL included CBC intermediates like fructose 1,6-bisphosphate (FBP), SBP, and RuBP that rose by 36%, 91%, and 66%, respectively (Figure 2C; Supplemental Figure S7B; see also Figure 3F). This resembles the response in C_3_ plants (see Borghi et al., 2019 and references therein). Levels of intermediates involved in starch and sucrose synthesis showed varied responses, with a significant two-fold increase of ADPG, no change of G6P and G1P and a significant 30% decrease in UDPG in ML compared to LL (Figure 2D; Supplemental Figure S7C; Figure 3G). The latter might point to post-translational activation of sucrose phosphate synthase, analogous to what happens in C_3_ species (Lunn and MacRae, 2003; Stitt et al*.*, 2010).

Intermediates involved in intercellular metabolite shuttles also showed a varied response. The active pool of malate was similar in LL and ML, the active pool of aspartate was even larger in LL than ML (Figure 2A), and the combined malate and aspartate active pool was also larger in LL than ML (Figure 3, A and B). Other metabolites that participate in intercellular shuttles had larger pool sizes in ML than LL, but the increase was much smaller than the approximately three-fold increase in the rate of photosynthesis. The combined pool size of PEP, pyruvate, and alanine increased by ∼70% (Figure 3C; Supplemental Table S5), and that of 3PGA and triose-P by only ∼25% (Figure 3D; Supplemental Table S5). These observations indicate that metabolite pools are used less effectively to facilitate intercellular movement in LL than in ML, with an especially large effect for malate and aspartate.

The level of 2PG was similar in LL and ML, the active pools of glycine, serine, and glycerate were significantly larger (42%, 104%, and 544%, respectively) in LL than in ML (Figure 2E; Supplemental Figure S7E), and there was three-fold more C in the combined active pool of glycine, serine, and glycerate in LL than in ML (Figure 3H). This is at first sight unexpected, as the lower rate of RuBP regeneration in LL might be expected to lead not only to a lower rate of RuBP carboxylation but also to a lower rate of RuBP oxygenation and, hence, lower levels of photorespiratory intermediates in LL than in ML. The implication is that CO_2_ levels in the BSC may be lower in LL than in ML, leading to a higher rate of RuBP oxygenation and photorespiration in LL than in ML (see later for more discussion).

Levels of glutamate and 2OG were high in LL and decreased significantly by approximately two-fold in ML (Figure 2F). These metabolites were labeled very slowly (see Figure 1; Supplemental Figure S6F). Levels of fumarate, valine, and threonine increased significantly in LL compared to ML, while succinate remained unaltered (Figure 2G). However, it is important to note that their absolute levels were much lower than the metabolites that are directly involved in C_4_ photosynthesis.

### Differences in temporal labeling kinetics in ML and LL

**Figure 4:**
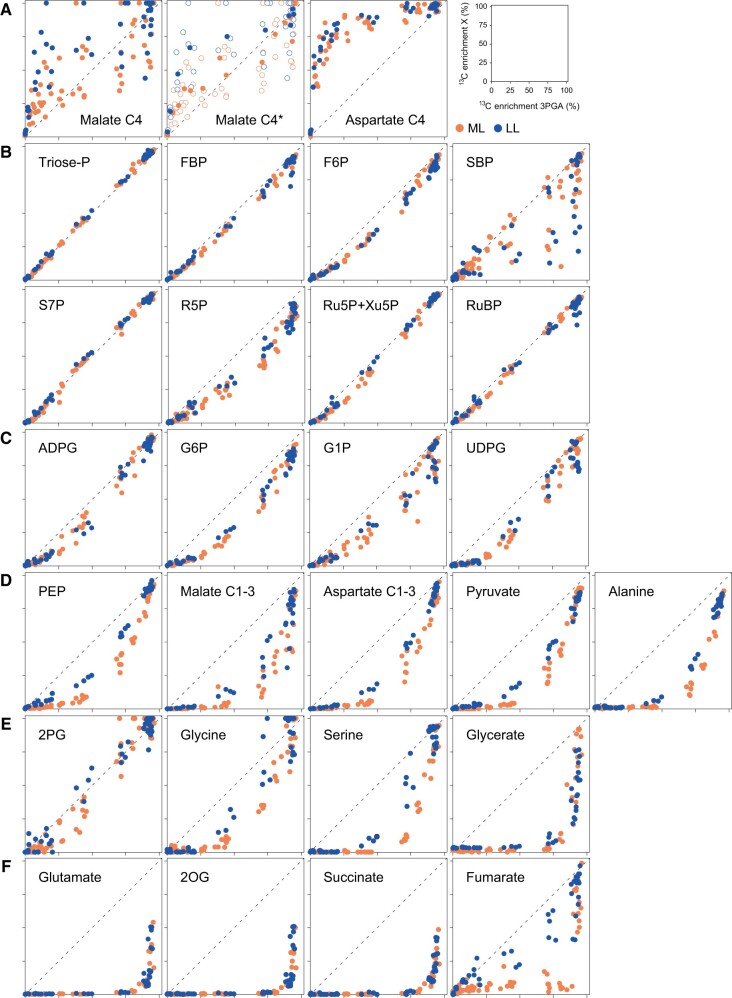
Regression plots of ^13^C enrichment of 3PGA versus ^13^C enrichment of all other metabolites and positional ^13^C enrichment for malate and aspartate. Unless otherwise stated, plots show all individual samples, with ^13^C enrichment of 3PGA on the *x*-axis and ^13^C enrichment of metabolite X on the *y*-axis. The identity of metabolite X is indicated in the panel. For the ^13^C enrichment of the C4 position of malate, a regression plot using mean values was also done (*n* = 3–6 and 2–6 replicates in ML and LL, respectively) and superimposed to the one obtained with individual samples. This plot is indicated as Malate C4*. A, C4 positions of malate and aspartate; B, CBC intermediates; C, starch and sugar synthesis intermediates; D, PEP, C1–3 positions of malate and aspartate, pyruvate and alanine; E, photorespiratory intermediates; and F, other organic acids and amino acids. Each axis runs from 0% to 100%, with intervals of 25%. The dotted line indicates a regression with a slope of 1. Regressions are shown for medium light (ML) and for low light (LL). Data are presented in Supplemental Data Set S6 and Supplemental Table S3. For abbreviations, see legend of Figure 1.

Although the labeling kinetics of the various clusters in Figure 1 were similar in LL and ML, close inspection indicated some differences, for example, clusters I and III were closer together in LL than in ML. Also, as already noted, some metabolites were assigned to different clusters in LL and ML. Comparison of labeling kinetics in ML and LL is complicated by the differing rates of photosynthesis. To compare labeling kinetics independently of the rate of ^13^C incorporation we plotted the ^13^C enrichment of each intermediate against that of 3PGA (Figure 4, original values are provided in Supplemental Data Set S6). We will first explain the rationale of the plot, and then how it reveals changes in flux patterns or poising of reactions between LL and ML.

3PGA is the first product of ^13^CO_2_ fixation by Rubisco. If an intermediate is labeled at the same rate as 3PGA (i.e. is in isotopic equilibrium with 3PGA) in a regression of the enrichment in that intermediate against enrichment in 3PGA the data points will lie around a straight line with a slope of 1. This theoretical relationship is indicated by a dotted line in the plots in Figure 4. If data points lie above the dotted line the intermediate is labeled more rapidly than 3PGA, and if they lie below the dotted line the intermediate is labeled more slowly than 3PGA. In regressions with the C4 positions of malate or aspartate, the data points lay above the dotted line (Figure 4A). This is expected since the C4 positions represent ^13^C that is being shuttled into the BSC. The noise in the plot for malate may reflect errors in estimating its pool size and hence the level of ^13^C enrichment in the active malate pool. Enrichment of most other CBC intermediates rose in parallel with that of 3PGA (Figure 1) and, correspondingly, they lay on or close to the dotted line (Figure 4B). This reflects the very fast half-lives of CBC intermediates (<1 s, Stitt et al., 1980, Arrivault et al., 2009). The plot for SBP was noisy, especially in LL, probably for the same reasons as that for malate. Among the intermediates in starch and sucrose synthesis, ADPG was labeled slightly more slowly than 3PGA (clusters II and I in ML and LL, respectively; Figure 1) and lay just below the dotted line (Figure 4C), whereas other intermediates such as G6P, G1P, and UDPG were labeled more slowly (mainly cluster II, Figure 1) and therefore lay on a curve below the dotted line (Figure 4C). As already mentioned, these intermediates have a relatively small stromal pool and a large cytosolic pool, which are labeled slowly. Cytosolic pools may also explain the slight displacement of F6P and R5P below the dotted line (see Figure 4B). PEP, the C1–3 positions of malate and aspartate, pyruvate and alanine were labeled much more slowly than 3PGA (cluster III, Figure 1) and lay on a curve well below the dotted line (Figure 4D). This reflects the relatively slow exchange of C between these metabolites and newly fixed ^13^C in the CBC (see above) and the large amount of C in these 3-carbon backbones. Photorespiratory intermediates downstream of 2PG were labeled slowly (mainly clusters II–IV in Figure 1) and lay on curves below the dotted line, with the curve being increasingly displaced for glycine, serine, and glycerate, reflecting pathway sequence (Figure 4E). Further organic acids and amino acids including fumarate, 2OG, and glutamate showed an even larger displacement in the plots against 3PGA (Figure 4F). Overall, the patterns in the normalized plots of Figure 4 resemble those seen following *k*-means clustering (Figure 1), but further aid comparison between ML and LL.

If flux patterns and poising of reactions are similar in LL and ML, the corresponding data points (shown in Figure 4 as blue and orange, respectively) will be superimposed. This was largely the case for aspartate (Figure 4A), intermediates in the CBC (Figure 4B), sucrose and starch biosynthesis (Figure 4C), glutamate, 2OG, and succinate (Figure 4F). For other intermediates, however, the ML and LL data points diverged. This included the C4 position of malate (Figure 4A, an additional regression plot using mean values was also performed, indicated as Malate C4*). Despite experimental noise, enrichment relative to 3PGA rose faster in LL than in ML (see later for more analysis). There was a clear separation between ML and LL for PEP, the C1–3 position of malate, the C1–3 position of aspartate, pyruvate, and alanine, whose enrichment relative to 3PGA rose more quickly in LL than ML (Figure 4D). Enrichment in photorespiratory intermediates relative to 3PGA rose more quickly in LL than in ML (Figure 4E), with the separation being slight for 2PG, large for glycine and serine but not obvious for glycerate. The latter may be due to the much larger pool size of glycerate in LL (see Figure 2; Supplemental Figure S7E). An even more striking, separation was seen for fumarate whose enrichment, relative to 3PGA, rose much more quickly in LL than in ML (Figure 4F).

### Estimation of fluxes in ML and LL

To allow a more quantitative comparison of ML and LL, we used initial rates of ^13^C accumulation to estimate flux in different sectors of C_4_ photosynthesis (Table 1). We took this approach rather than a full flux model due to there being considerable redundancy in C_4_ photosynthesis, and because data were lacking for key parameters that will affect fluxes in intercellular shuttles, including the magnitude of intercellular concentration gradients, transport steps at the chloroplast envelope that may modify cytosolic concentrations, plasmodesmatal conductance, and Φ. The partial fluxes that we estimated included (1) H^13^CO_3_ assimilation by PEPC, (2) the contribution of malate and aspartate to the CCM, (3) ^13^CO_2_ fixation by Rubisco, (4) exchange of ^13^C from CBC intermediates into metabolites involved in the CO_2_ shuttle, and (5) flow of ^13^C into photorespiration. Such estimates were made by summing the amount of ^13^C in the relevant subset of metabolites (or relevant positions in metabolites) at each time point (Supplemental Data Set S10), plotting them against time, identifying the time interval in which ^13^C accumulation was fastest, determining the slope for this time interval, and correcting this for enrichment in the immediate precursor. As different sectors of metabolism label at different rates, we used different time ranges for the estimation of each flux (Table 1; Supplemental Data Set S10).

**Table 1:** Estimation of fluxes through PEPC, the C4 positions of malate and aspartate, Rubisco, the C carriers in CO_2_ shuttle, and photorespiration in ML and LL

		ML			LL	
Parameter	Value	Confidence interval (95%)	Time points used	Value	Confidence interval (95%)	Time points used
**Rate of photosynthesis (gas exchange),** nmol CO_2_ g^−1^ FW s^−1^	**122.9 ± 8.2**			**45.5 ± 3.9**		
**Estimated flux at PEPC** (Rate of ^13^C accumulation in all measured intermediates), nmol ^13^C g^−1^ FW s^−1^	121.3	± 51.26	0–5 s	62.8	± 13.3	0–10 s
Rate of ^13^C accumulation in C4 positions of malate and aspartate, nmol ^13^C g^−1^ FW s^−1^	86.8	± 37.3	0–5 s	52.6	± 6.38	0–10 s
Rate of ^13^C accumulation in all measured intermediates except C4 positions of malate and aspartate, nmol ^13^C g^−1^ FW s^−1^	34.5	± 16.28	0–5 s	10.2	± 11.42	0–10 s
**Rate of ^13^C accumulation in C4 position of malate,** nmol ^13^C g^−1^ FW s^−1^	**48.6**	± 37.92	0–5 s	**24.7**	± 6.97	0–10 s
**Rate of ^13^C accumulation in C4 position of aspartate,** nmol ^13^C g^−1^ FW s^−1^	**38.2**	± 7.36	0–5 s	**27.9**	± 9.25	0–10 s
Rate of ^13^C accumulation in all measured intermediates except C4 positions of malate and aspartate, nmol ^13^C g^−1^ FW s^−1^	61.9	± 4.9	0-30s	22.4	± 1.74	0–180 s
Estimated movement of ^13^C to starch and sucrose, nmol ^13^C g^−1^ FW s^−1^	2.9		0–30 s	2.8		0–180 s
Total estimated ^13^C accumulation, nmol ^13^C g^−1^ FW s^−1^	64.8		0–30 s	25.2		0–180 s
Average ^13^C enrichment in C4 positions of malate and aspartate, %	31.5–71		0–30 s	65.1–80.5		0–180 s
**Estimated flux at Rubisco,** nmol C g^−1^ FW s^−1^	**91.3–205.7**		030 s	**31.3–38.7**		0–180 s
Rate of ^13^C accumulation in C1–3 positions of malate and aspartate plus pyruvate, alanine, and PEP, nmol ^13^C g^−1^ FW s^−1^	13.5	± 1.1	15–300 s	6.9	± 0:8	30–600 s
Average ^13^C enrichment of 3PGA (%)	60.2		15–300 s	50.7		30–600 s
**Estimated flux between CBC and CCM,** nmol C g^−1^ FW s^−1^	**22.4**		15–300 s	**13.6**		30–600 s
Rate of ^13^C accumulation in alanine, nmol ^13^C g^−1^ FW s^−1^	2		15–300 s	0.64		30–600 s
Rate of ^13^C accumulation in pyruvate, nmol ^13^C g^−1^ FW s^−1^	2		15–300 s	0.52		30–600 s
Rate of ^13^C accumulation in photorespiratory intermediates, nmol ^13^C g^−1^ FW s^−1^	0.52	± 0.06	50–600 s	0.75	± 0.09	60–600 s
Estimated ^13^C released by glycine decarboxylase, nmol ^13^C g^−1^ FW s^−1^	0.11		50–600 s	0.18		60–600 s
Estimated ^13^C entering photorespiration, nmol ^13^C g^−1^ FW s^−1^	0.63		50–600 s	0.93		60–600 s
Average ^13^C enrichment in RuBP, %	70.1		50–600 s	50.3		60–600 s
**C entering photorespiration,** nmol CO_2_ g^−1^ FW s^−1^	**0.89**		50–600 s	**1.84**		60–600 s
**Rate of RuBP oxygenation,** nmol O_2_ g^−1^ FW s^−1^	**0.45**		50–600 s	**0.92**		60–600 s
**Relative rates of oxygenation and carboxylation, ratio**	**0.004**		50–600 s	**0.02**		60–600 s

The total ^13^C amounts in different metabolic sectors were calculated by summing ^13^C from the relevant subset of metabolites, or relevant positions in metabolites (Supplemental Data Set S10). The time intervals with the fastest rate of ^13^C accumulation were identified and used to determine the slope in this time period, giving estimates of ^13^C flux (nmol ^13^C g^−1^ FW s^−1^). Time intervals used are indicated, as well as confidence intervals (95%). The estimated flux at Rubisco was calculated by adding the ^13^C amounts in the end-products starch and sucrose to the estimated rate of ^13^C accumulation in all intermediates except C4 positions of malate and aspartate and correcting this sum by the weighted ^13^C enrichments in C4 positions of malate and aspartate. The estimated flux between CBC and CCM was obtained by correcting the rate of ^13^C accumulation in C1–3 positions of malate and aspartate plus pyruvate, alanine, and PEP by the weighted ^13^C enrichment in 3PGA. The rate of ^13^C accumulation in photorespiratory intermediates was corrected for the release of 25% of the label that moves beyond glycine decarboxylase by adding the estimate of ^13^C released by glycine decarboxylase (obtained by multiplying the rate of ^13^C accumulation in photorespiratory intermediates by 1/4 and by (serine and glycerate pool/total photorespiratory metabolite pool) using data from Supplemental Table S2). C entering photorespiration was obtained by correcting the estimated ^13^C entering photorespiration by the weighted ^13^C enrichment of RuBP. Each oxygenation reaction leads to two C atoms in 2PG, therefore C entering photorespiration was divided by two in order to obtain the rate of RuBP oxygenation. The rate of oxygenation and carboxylation was obtained by dividing the rate of RuBP oxygenation by the rate of photosynthesis obtained by gas exchange. The determination of an estimate of movement of ^13^C to starch and sucrose, and weighted ^13^C enrichments during selected time intervals are presented in Supplemental Data Set S11. The rate of photosynthesis determined by gas exchange is included in this table. Estimated fluxes are in bold, while the calculation steps are not. Estimated fluxes based on leaf area are presented in Supplemental Table S6. 3PGA, 3-phosphoglycerate; CBC, Calvin-Benson cycle; CCM, carbon concentration mechanism; LL, low light; ML, medium light; PEP, phosphoenolpyruvate; PEPC, phosphoenolpyruvate carboxylase; RuBP, ribulose 1,5-bisphosphate.

### Flux through PEPC and concentration of inorganic C into the BSC

We estimated flux through PEPC by summing the ^13^C that accumulated in all measured intermediates up to 5 s in ML and 10 s in LL (121.3 and 62.8 nmol ^13^C g^−1^ FW s^−1^ at ML and LL, respectively; Table 1). These will be underestimates as they do not include ^13^C that has been released as ^13^CO_2_ in the BSC but not yet assimilated by Rubisco. For comparison, the rates of photosynthesis in ML and in LL measured by gas exchange were 122.9 ± 8.2 and 45.5 ± 3.9 nmol CO_2_ g^−1^ FW s^−1^, respectively. Values for these and other estimated fluxes on a leaf area basis are provided in Supplemental Table S6.

We compared the amount of ^13^C en route to the BSC (i.e. in the combined ^13^C amounts at the C4 positions of malate and aspartate) with the amount of ^13^C in downstream intermediates formed from ^13^CO_2_ released in the BSC (Table 1). Comparing the 5-s pulse in ML and the 10-s pulse in LL, the initial rate of ^13^C accumulation in the combined C4 positions of malate and aspartate was approximately 1.5-fold higher in ML than in LL (86.8 and 52.6 nmol ^13^C g^−1^ FW s^−1^, respectively; confidence intervals were large for the estimate in ML), the rate of ^13^C accumulation in downstream metabolites was approximately three-fold higher in ML than in LL (34.5 and 10.2 nmol ^13^C g^−1^ FW s^−1^, respectively) and the C4 positions represented a smaller proportion of the total detected ^13^C in ML than in LL (71.5% and 84%, respectively). Thus, ^13^C moves through the combined C4 pool more quickly in ML than in LL, not only in absolute terms but also relative to flux through PEPC.

### Contributions of malate and aspartate to the CO_2_ shuttle

**Figure 5:**
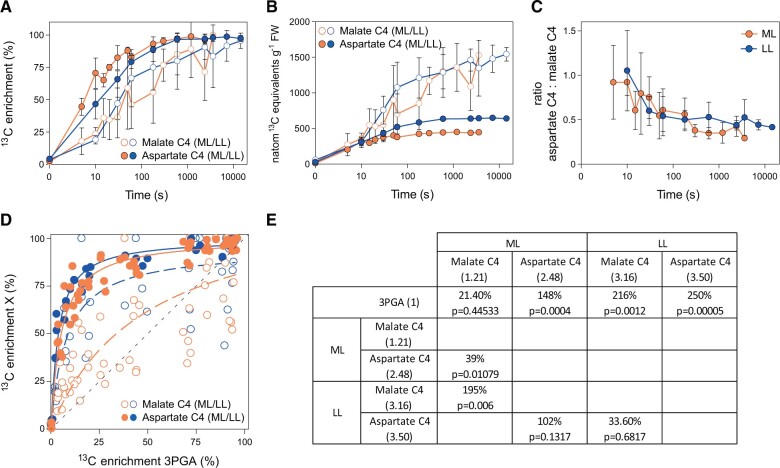
Labeling kinetics, ^13^C amounts in the C4 positions of malate and aspartate and ^13^C enrichment of 3PGA versus ^13^C enrichment in the C4 positions of malate and aspartate. A, ^13^C enrichment (%) of the C4 positions of malate and aspartate. B, ^13^C amounts (natoms ^13^C equivalents g^−1^ FW) in the C4 positions of malate and aspartate. C, Ratio of ^13^C amount in the C4 position of aspartate: ^13^C amount in the C4 position of malate. D, Regression plot of ^13^C enrichment of 3PGA (*x*-axis) versus ^13^C enrichment of the C4 positions of malate and aspartate (*y*-axis) in medium light (ML) and low light (LL). All individual samples are shown. Fitted curves are presented (dotted for C4 position of malate, solid for C4 position of aspartate). The black dotted line indicates a regression with a slope of 1. In (A), (B), and (D), C4 malate is shown as open symbol and C4 aspartate as filled circle. Time point zero was not included in (B). For (A), (B), and (C), mean ± sd are presented, with *n* = 3–6 and 2–6 replicates in ML and LL, respectively. Their *x*-axes correspond to the labeling time on a log scale. E, Comparison of slopes from the fitted curves shown in (D) and statistical analyses. Slopes (indicated in bracket) were estimated for all pulses in which 3PGA enrichment was <30%. The differences between slopes are expressed as percentages, and *P*-values are indicated. Data for (A), (D), and (E) are presented in Supplemental Table S3 and Supplemental Data Set S6. Data for (B) and (C) are presented in Supplemental Table S4.

To assess the contribution of NADP-ME compared to alternative routes involving NAD-ME or PEPCK, we first compared the labeling kinetics and pool sizes of the C4 positions of malate and aspartate. In both ML and LL, ^13^C enrichment rose more quickly in the C4 position of aspartate than in the C4 position of the active pool of malate (Figure 5A; Supplemental Figure S6A). The active malate pool was always much larger than the aspartate pool, the active malate pool had a similar size in ML and LL, and the aspartate pool was 30% smaller in ML than in LL (Figure 2A). Taking these pool sizes into account, the initial rate of ^13^C incorporation was faster for malate than aspartate in ML (48.6 compared to 38.2 nmol ^13^C g^−1^ FW s^−1^) and marginally faster for aspartate than malate in LL (27.9 compared to 24.7 nmol ^13^C g^−1^ FW s^−1^) (Figure 5B and Table 1). These initial labeling kinetics point to aspartate making a larger contribution to the CCM in LL than in ML. However, the estimates for the C4 position of malate in ML had a large confidence interval. They were complicated by corrections that were needed to exclude its inactive pool (see above). Furthermore, the relation between the amount of ^13^C in the C4 positions of malate and aspartate changed with time (Figure 5C). Whereas the ratio of ^13^C in aspartate and malate was close to 1 at the earliest times in ML (5–10 s) and LL (10 s), it declined (to ∼0.5 in LL and ∼0.4 in ML) in the next 60–150 s. These observations indicate that in both ML and LL there is faster initial accumulation of H^13^CO_3_ into the C4 position of aspartate than malate and faster turnover of the ^13^C in the C4 position of aspartate than of malate, which further complicates estimation of flux from the labeling kinetics.

To provide supporting evidence for our estimates of the labeling kinetic of the active malate pool, we explored whether labeling kinetics of fumarate could be used as a proxy for malate. Label is exchanged between malate and fumarate via a reversible reaction catalyzed by fumarase in the mitochondria. Importantly, the active fumarate pool was approximately 1,000-fold smaller than the active malate pool (Figure 2, A and G; Supplemental Table S2). Overall enrichment rose quite quickly in fumarate (Figures 1 and 4, F) to reach ∼80% enrichment at later time points (see also Supplemental Table S3 and Supplemental Figure S6F). We estimated labeling kinetics separately for the C4 and the C1–3 positions of fumarate (Supplemental Figure S8) using the procedure described above for malate and aspartate. In LL, estimated enrichment at the C4 position of fumarate and of malate were almost identical (Supplemental Figure S8, A and B), providing strong support for our estimates of ^13^C enrichment at the C4 position of malate. In ML, estimated enrichment at the C4 position rose more slowly in fumarate than in malate (Supplemental Figure S8, C and D). Enrichment in the C1–3 position of fumarate rose with a delay, resembling the response in malate and aspartate. When enrichment is compared in the C1–3 position of fumarate and malate, in LL enrichment rose in both in a similar manner and in ML enrichment rose more slowly in fumarate than in malate. This highlights the potential complexity of malate labeling kinetics in ML (see “Discussion”).

Together, these analyses pointed to the need for caution when comparing the initial labeling kinetics of malate and aspartate, especially in ML. Anyway, the rates of ^13^C accumulation in the C4 position of malate or aspartate do not, on their own, provide a reliable measure of flux. This will also depend on how quickly ^13^C is released by NADP-ME (for malate) or NAD-ME and PEPCK (for aspartate). Unfortunately, the three decarboxylation reactions release ^13^C into a shared C_BSC_ pool. This makes it impossible to distinguish their individual contributions to ^13^C that accumulates in metabolites downstream of the C_BSC_ pool.

These complications led us to explore an alternative approach to assess the contribution of malate- and aspartate-based decarboxylation routes, based on the relationship between ^13^C enrichment in the C4 positions of malate and aspartate and ^13^C enrichment in 3PGA (Figure 4A). At any given time, ^13^C enrichment in 3PGA will depend on ^13^C enrichment of C_BSC_ and ^13^C enrichment of RuBP (contributing one-sixth and five-sixth, respectively). Importantly, it is known that enrichment in C_BSC_ closely follows ^13^C enrichment in the precursor C4 positions, and that the C_BSC_ pool size is only 2%–5% of that of the C4 positions and therefore turns over rapidly (Hatch 1971; Furbank and Hatch, 1987). Due to the high contribution of the enrichment of RuBP to the enrichment in 3PGA (five-sixth) and, due to the rapid turnover of the CBC, enrichment of RuBP is very similar to that of 3PGA (Figures 1 and 4, B; Supplemental Figure S6B). This simplifies the following analysis because it means that enrichment in 3PGA will be very sensitive to enrichment in C_BSC_. As already mentioned, enrichment rises more slowly in 3PGA than in the C4 positions of malate and aspartate (Figures 1 and 4) and, by implication C_BSC_. This delay is due to the large pool of C in CBC metabolites (Figure 3, E and F; Supplemental Table S5). Enrichment of C_BSC_ will depend on four factors: (1) ^13^C enrichment in the C4 position of malate; (2) ^13^C enrichment in the C4 position of aspartate; (3) relative flux through malate- and aspartate-based decarboxylation routes; and (4) relative flux of CO_2_ into the BSC from unlabeled pools, for example, photorespiration or mitochondrial respiration. In Figure 5, D and E the relationship between ^13^C enrichment in the individual C4 positions of malate and aspartate and ^13^C enrichment in 3PGA is analyzed; fitted curves are superimposed to visualize the main trends, and identical enrichment to 3PGA is indicated as a dotted gray line (slope = 1). Estimation of slopes and testing for statistical significance were done for all time points at which 3PGA enrichment was <30%. Compared to enrichment of 3PGA, in LL enrichment was much higher for the C4 positions of both malate (3.16) and aspartate (3.5). Compared to LL, enrichment in ML was significantly decreased for the C4 position of malate (1.21) and nonsignificantly decreased for the C4 position of aspartate (2.48). This is consistent with a smaller relative influx of unlabeled CO_2_ from internal pools in ML than in LL. Independent of such effects, light intensity modified the relationship between enrichment in 3PGA and enrichment in the C4 positions of malate and aspartate. In ML, enrichment in the C4 position of malate was weakly (21.4%) and nonsignificantly displaced above the dotted line that indicates isotopic equilibrium with 3PGA, enrichment in the C4 position of aspartate was strongly (148%) and significantly displayed from the dotted line, and the difference between the response of the C4 positions of malate and aspartate was significant. This is the pattern expected if malate contributes most of the ^13^C entering the C_BSC_ pool. In LL, the C4 positions of malate and aspartate were strongly and significantly displaced from the dotted line (by 216% and 250%, respectively) and the difference between the response of the C4 positions of malate and aspartate was much smaller and nonsignificant, pointing to a larger relative contribution by aspartate-based routes in LL.

### Rate of ^13^CO_2_ assimilation in the CBC

Minimum rates of ^13^C assimilation by Rubisco were estimated from ^13^C incorporation in all measured metabolites except the C4 positions of malate and aspartate during the time intervals 0–30 s in ML and 0–180 s in LL (61.9 and 22.4 nmol ^13^C g^−1^ FW s^−1^ in ML and LL, respectively, Table 1). It is important to note that these might be underestimates as even in this short time interval some ^13^C will have moved into end-products that our analyses did not detect. The magnitude of this underestimation was assessed by estimating the likely flux of C to the main end-products, starch, and sucrose, using ^13^C enrichments in immediate precursors (see legend of Table 1 and Supplemental Data Set S11). Due to the very low ^13^C enrichment in the precursors in the initial phase of labeling (Supplemental Figure S6), estimated movement of ^13^C into starch and sucrose was very low (2.9 and 2.8 nmol ^13^C g^−1^ FW s^−1^ in ML and LL, respectively). These values were added to those for ^13^C in analyzed metabolites to give an estimated rate of ^13^C incorporation by Rubisco of 64.8 and 25.2 nmol ^13^C g^−1^ FW s^−1^ in ML and LL, respectively. At these early time points, ^13^C incorporation will underestimate the rate of CO_2_ fixation because enrichment in the C_BSC_ pool will not have reached 100%. To correct for this effect, we assumed (for support, see Hatch, 1971; Furbank and Hatch, 1987) that average ^13^C enrichment in the C_BSC_ pool would resemble enrichment in the C4 positions of malate and aspartate. The average value in ML and LL during the relevant time interval was 31% and 71% for malate, and 65% and 80% for aspartate, respectively (Supplemental Data Set S11). Depending on the relative contribution of malate and aspartate to C transfer, the rate of C fixation at Rubisco was estimated to be the range of 91.3–205.7 nmol C g^−1^ FW s^−1^ in ML and 31.3–38.7 nmol C g^−1^ FW s^−1^ in LL, with the higher value being the case that only malate contributed and the lower value being the unlikely case that only aspartate contributed to CO_2_ concentration. The precision of the estimate for ML is affected by errors in assessing enrichment in the C4 position of malate in short pulses. This error is likely to be small in LL, where the enrichment in the C4 position of malate is supported by a similar value for fumarate (see above and Supplemental Figure S8). Fluxes will also be underestimated if unlabeled CO_2_ from internal sources enters C_BSC_ and decreases its ^13^C enrichment compared to the precursor C4 positions.

In conclusion, in ML, estimated fluxes at PEPC and Rubisco were of the same order, if it is assumed that the large range for Rubisco value was due to uncertainties in estimating enrichment in the C4 position of malate, and that the actual value for flux at Rubisco was in the lower part of the range. In this case, estimated fluxes at PEPC and Rubisco resembled the net rate of CO_2_ fixation measured by gas exchange. In LL, estimated flux at PEPC was higher than estimated flux at Rubisco and higher than the net rate of CO_2_ fixation measured by gas exchange (Table 1).

### Exchange of C between CBC and CCM metabolites

Part of the ^13^C that is fixed in the CBC moves from 3PGA to PEP and from there into the C1–3 positions of malate and aspartate, and then into pyruvate and alanine. We term this “movement of ^13^C from the CBC to the CCM”. The rise in enrichment in PEP was indeed closely followed by a rise in enrichment in other CCM metabolites, both in ML and in LL (Figures 1 and 4, D; Supplemental Figures S7, D and S9, A and B). Inspection of enrichment kinetics revealed that PEP was closer to isotopic equilibrium with 3PGA in LL than in ML (Figure 4D; see also Supplemental Figure S10). This indicates that C derived from the CBC accounts for a larger proportion of the C entering the PEP pool in LL than ML, and that the contribution from the CO_2_ shuttle increases in ML.

To estimate flux between the CBC and PEP, we summed ^13^C in PEP, the C1–3 positions of malate and aspartate, and pyruvate and alanine between 15 and 300 s in ML and between 30 and 600 s in LL (13.5 and 6.9 nmol ^13^C g^−1^ FW s^−1^, respectively) and corrected it for average enrichment in 3PGA over this time interval (∼60% in ML and ∼51% in LL; Supplemental Data Set S11). This calculation yielded an estimated flux of 22.4 and 13.6 nmol C g^−1^ FW s^−1^ in ML and LL, respectively (Table 1). At steady state, movement of C from 3PGA to PEP will be balanced by movement of C from PEP to 3PGA, with phosphoglycerate mutase and enolase catalyzing C exchange but no net flux (see “Discussion”). When active metabolite pools sizes are inspected, the PEP:3PGA ratio (0.37 and 0.39 in ML and LL, respectively, Figure 2; Supplemental Table S4) was indeed close to that expected at thermodynamic equilibrium.

We compared the rate of C exchange between the CBC and the CCM (Table 1) with the total pool of CBC and the CCM metabolites (Supplemental Table S5). The estimated rate of C exchange between the CBC and the CCM was equivalent to 0.26% and 0.17% of summed C in PEP, the C1–3 positions of malate and aspartate, and pyruvate and alanine in ML and LL, respectively and 0.36% and 0.26% of summed C in 3PGA, triose-P, and other CBC intermediates in ML and LL, respectively. This reflects the large pools of metabolites in intercellular shuttles. Exchange of C between the CBC and the CCM is nevertheless quite fast compared to the rate of photosynthesis (about 18% and 30% of the rate of CO_2_ fixation in ML and LL, respectively; estimated using values from gas exchange). The faster exchange relative to the rate of photosynthesis in LL was reflected in clusters I and III being closer in LL than in ML (Figure 1) and the smaller lag in the labeling kinetics of PEP, the C1–3 positions of malate and aspartate, and pyruvate and alanine in LL compared to ML (Figure 3D). The absolute rate of exchange was ∼65% higher in ML compared to LL.

To inspect the flow of label downstream of PEP independently of changes in rate of ^13^C assimilation, we plotted enrichment in PEP against enrichment in the C1–3 positions of malate and aspartate, and pyruvate and alanine (Supplemental Figure S9C). The relationship was similar in ML and LL and exhibited two notable features. First, relative to PEP, enrichment rose more slowly in the C1–3 position of malate than in the C1–3 position of aspartate or pyruvate. This is curious because pyruvate lies downstream of malate in the NADP-ME decarboxylation route. The slow rise of enrichment in malate might reflect errors in estimating enrichment in the malate active pool. However, enrichment in the C1–3 position of malate rose in parallel with that of fumarate in LL, but more rapidly than in fumarate in ML (Supplemental Figure S8, B and D). These observations might indicate that the active malate pool in ML actually contains two pools (as also indicated by analysis of labeling of the C4 position of malate, see above) with one of them being labeled somewhat more slowly, closer to equilibrium with fumarate, not equilibrating rapidly with aspartate and not being directly converted to pyruvate (see also “Discussion”). Second, relative to PEP, enrichment rose more slowly in alanine than in pyruvate. This is at least partly explained by the two-fold small pool size of pyruvate than alanine (Figure 2A; Supplemental Figure S7D). The rate of label accumulation in alanine and pyruvate (Table 1) was similar in ML (2 nmol ^13^C g^−1^ FW s^−1^), and slightly (23%) faster in alanine than pyruvate in LL (0.64 and 0.52 nmol ^13^C g^−1^ FW s^−1^). It is surprising that ^13^C accumulates rapidly in alanine in a nominally NADP-ME subtype plant (see “Discussion”).

### Photorespiration

Movement of ^13^C into photorespiration was estimated as the rate of accumulation of ^13^C in 2PG, glycine, serine, and glycerate between 50 and 600 s in ML and 60 and 600 s in LL, corrected for the release of 25% of the label that moves beyond glycine decarboxylase as ^13^CO_2_, giving estimates of 0.63 and 0.93 nmol ^13^C g^−1^ FW s^−1^ in ML and LL, respectively. These may be slight underestimates, because they were not corrected for ^13^C that had exited the glycerate pool, but the underestimate is likely to be small as glycerate enrichment was still relatively low (averaging 10.4% and 6% in ML and LL, Supplemental Data Set S11) in the time range used for our estimates. They may also be underestimates if some glycine, serine, or glycerate is exported out of the photorespiratory pathway. This has been shown to occur in C_3_ plants, especially for serine (Busch et al., 2018). As the enrichment in glycine and serine is already quite high in the time range used for our estimates (45.8% and 38.5% for glycine and 25.2% and 24.9% for serine in ML and LL, respectively; Supplemental Data Set S11), it is possible that some ^13^C exits the photorespiration pathway in glycine or serine. However, on its own, this would not lead to an underestimation of the flow of ^13^C into the photorespiration pathway. This would only be the case if glycine or serine were converted into other metabolites or were exported out of the area of the leaf that was labeled and harvested for analysis.

Average enrichment in RuBP over this time interval was 70.1% in ML and 50.3% in LL (Supplemental Data Set S11). Combined with the rate of accumulation of ^13^C in 2PG, glycine, serine and glycerate, this gives a minimum estimated flux of C into photorespiration of 0.89 and 1.84 nmol C g^−1^ FW s^−1^, in ML and LL, respectively. This is equivalent to a rate of RuBP oxygenation of 0.45 and 0.92 nmol O_2_ g^−1^ FW s^−1^ of net CO_2_ fixation in ML and LL, respectively. Thus, the rate of RuBP oxygenation and photorespiration is faster in LL than in ML, not only relative to the rate of photosynthesis but also in absolute terms. This may explain why the pools of glycine, serine, and especially glycerate are larger in LL than in ML (Figure 4H; Supplemental Figure S7E).

## Discussion

### Changes in flux patterns between ML and LL point to decreased effectiveness of the CCM in LL

Our main aim was to investigate if C_4_ photosynthesis becomes less efficient in low irradiance. To do this, we grew maize at moderate irradiance (550 µmol m^−2^ s^−1^) and performed ^13^CO_2_ pulse labeling at this irradiance (ML) or after several hours in low irradiance (160 µmol m^−2^ s^−1^, LL). A redesigned labeling set-up allowed precise and very short pulses, resulting in labeling kinetics that were superior to those of Arrivault et al. (2017). Many aspects of the labeling kinetics, shared between ML and LL, confirmed and extended previous ideas about the operation of C_4_ photosynthesis (see Supplemental Text S2). However, detailed comparison of pool sizes (Figures 2, 3, and 5), labeling kinetics (Figures 1 and 4), and estimated fluxes (Table 1) revealed important differences between ML and LL. These pointed to photosynthetic efficiency being decreased in LL and revealed that this occurs for several interlocking reasons; less effective use of metabolite pools to drive intercellular metabolite exchange, less effective decarboxylation by NADP-ME, increased operation of alternative and possibly less effective decarboxylation routes, increased photorespiration and increased back-leakage of CO_2_. The evidence and possible consequences are discussed in the following sections.

### Intercellular movement and decarboxylation of 4-carbon CCM metabolites is impaired in LL

Earlier studies of changes in metabolite levels in maize leaves found that, after illumination, RuBP levels transiently rose to a high level then declined, with the latter coinciding with a rise in the levels of CCM metabolites like malate, aspartate, and pyruvate (Usuda, 1985). They also found that steady-state RuBP levels are already high in low irradiance and, with increasing irradiance, remain unaltered (Usuda, 1987) or rise only slightly (Leegood and von Caemmerer, 1989), whereas the levels of several CCM metabolites showed a large increase. These studies indicated that CCM operation may be restricted in LL. One explanation might be that, in LL, small pools constrain the size of intercellular concentration gradients and the rate with which metabolites diffuse between cells.

Using ^13^C labeling, we can estimate the size of pools that are directly involved in the CCM. Whereas the net rate of photosynthesis was almost three-fold lower in LL than in ML, the active pool of malate had a similar size in LL and ML, the aspartate pool was slightly larger in LL than in ML (Figures 2, A and 5, B) and, therefore, the combined active pool of malate and aspartate was slightly larger in LL than in ML. These observations imply that in LL intercellular movement is not constrained by pool size but, rather, by less efficient use of these pools to generate a concentration gradient in LL than in ML (i.e. that a given total pool generates a larger concentration gradient in ML than in LL).

This conclusion is supported by two features of the temporal labeling kinetics. First, the estimated rate of ^13^C accumulation in the C4 positions of malate and aspartate at the earliest time points in the labeling kinetic was only 65% larger in ML than in LL (86.8 and 52.6 nmol g^−1^ FW s^−1^, respectively) (Table 1; see also Figure 3A), which is much smaller than the 158% increase in the net rate of photosynthesis (Table 1). Second, the rate of accumulation of ^13^C in metabolites downstream of the C4 positions of malate and aspartate represented only 16.2% of the overall rate of accumulation of ^13^C in all analyzed metabolites after a 10-s pulse in LL compared to 28.4% after a 5-s pulse in ML (see Table 1) indicating that, relative to the initial rate of ^13^C incorporation, ^13^C moves more slowly through the C4 pools in LL than in ML.

### Aspartate-based decarboxylation routes play a larger role in LL

CO_2_ can be concentrated into the BSC via movement of malate and decarboxylation by NADP-ME, or via movement of aspartate and decarboxylation by NAD-ME or PEPCK (see “Introduction”). Total aspartate was previously reported earlier to be higher in low than high irradiance (Usuda, 1987; Doncaster et al., 1989; Ubierna et al*.*, 2013) although not in Leegood and von Caemmerer (1989). Our results show the pool of aspartate increases in LL, relative both to the aspartate pool in ML and to the active malate pool in LL and ML (Figure 2A). These observations are consistent with aspartate-based decarboxylation routes making a larger contribution in LL. This interpretation, however, assumes that flux is dependent on pool size and requires independent support from estimates of flux.

We first inspected the initial labeling kinetics of the C4 positions of malate and aspartate. At very early time points, ^13^C enrichment in the C4 position rose faster in aspartate than in malate, with the difference being larger in ML than in LL (Figure 5A). When the larger pool of malate is taken into account, the rate of ^13^C accumulation was slightly faster in the C4 position of aspartate than that of malate in LL, and faster in the C4 position of malate than that of aspartate in ML (Table 1 and Figure 5B). Interpretation is further complicated because the amount of label in the C4 position of aspartate relative to that in malate decreased with time (Figure 5C, see below for further discussion). Irrespective of these uncertainties, the rate of ^13^C accumulation in the C4 positions of malate or aspartate does not provide reliable information about flux. This requires information about ^13^C accumulation in metabolites downstream of the C4 positions of malate and aspartate. This information is not provided by our data because the decarboxylase reactions release ^13^C into a shared pool of CO_2_ in the BSC. We therefore explored other approaches to estimate flux through malate and aspartate.

One approach was based on consideration of nitrogen (N) stoichiometry. To maintain N stoichiometry, movement of aspartate to the BSC must be coupled with movement of an amino group back to the MC. We asked if the labeling kinetics of alanine and pyruvate point to an increased contribution of alanine in LL. The active pool of alanine was about two-fold larger than that of pyruvate and both decreased in a similar manner between ML and LL (by 42% and 46%, respectively, Figure 2A). This differed incidentally from Leegood and von Caemmerer (1989) and Ubierna et al. (2013) where the total alanine pool did not show a consistent change with decreasing irradiance. ^13^C enrichment in alanine and pyruvate rose in a similar manner in ML and LL, especially after taking the differing rates of photosynthesis into account (Figure 4D; Supplemental Figure S9C). Inspection of rates (Table 1) reveals that ^13^C accumulated in alanine at about the same rate as in pyruvate both in ML (2 and 2 nmol ^13^C g^−1^ FW s^−1^) and in LL (0.64 and 0.52 nmol ^13^C g^−1^ FW s^−1^). This finding is unexpected for two reasons. First, there is no evidence that the shift to labeling of aspartate in LL is accompanied by increased labeling of alanine relative to pyruvate. Even more surprisingly, alanine is labeled as quickly as pyruvate, even though the main route for decarboxylation in maize via NADP-ME requires exchange of malate with pyruvate rather than alanine.

A possible explanation for these unexpected observations is that the pool sizes and labeling kinetics of pyruvate and alanine do not provide unambiguous information about the contribution of malate and aspartate to the CCM. This would only be the case if the primary shuttles that transfer CO_2_ to the BSC are restricted to a malate/pyruvate and an aspartate/alanine exchange. It is however possible that N stoichiometry is maintained by secondary shuttles; for example, a shuttle involving glutamate and 2OG. These are both present at high levels in maize leaves (Figure 2F; Supplemental Table S2, see also Arrivault et al., 2017). They might allow further primary shuttles, with a malate/alanine exchange linked to NADP-ME, an aspartate/pyruvate shuttle linked to NAD-ME, or a shuttle in which aspartate moves to the BSC, is decarboxylated by PEPCK and PEP, 3PGA (see below) or pyruvate (see Bräutigam et al., 2018) move back to the MC. Secondary shuttles could operate without rapid labeling of the organic acid backbone by ^13^C, making them difficult to detect directly by ^13^C labeling. The unexpectedly rapid accumulation of label in alanine compared to pyruvate is, however, consistent with their operation. Such secondary shuttles would introduce further flexibility into C_4_ photosynthesis.

As an alternative approach to provide information about contributions of the C4 positions of malate and aspartate, we compared their estimated ^13^C enrichment with ^13^C enrichment of 3PGA (see Figures 4, A and 5, D and E). As explained in the “Results”, ^13^C enrichment in 3PGA will depend on ^13^C enrichment of C_BSC_, which itself depends on ^13^C enrichment in the C4 positions of malate and aspartate, and on the source of the CO_2_ entering the C_BSC_, that is the relative contributions of malate- and aspartate-based decarboxylation routes and unlabeled CO_2_ released from internal pools. ^13^C enrichment in 3PGA relative to the C4 positions of malate and aspartate was lower at early times in LL than in ML, which is consistent with a larger contribution of unlabeled CO_2_ from photorespiration or mitochondrial respiration in LL (see also Sage, 2014; Ubierna et al., 2013; Bellasio and Griffiths, 2014b). In ML, ^13^C enrichment in the C4 position of malate was quite close to ^13^C enrichment in the downstream metabolite 3PGA, whereas ^13^C enrichment in the C4 position of aspartate was much higher. This agrees with malate-based decarboxylation routes playing the main role in ML. The difference was much smaller in LL, pointing to a larger role of aspartate-based routes in LL. More precise quantification of the shift between malate and aspartate is difficult because the estimates of enrichment in the C4 position of malate are noisy, because it would require information about the rate of influx of unlabeled CO_2_ to the BSC and (see next section) because there may be more than one malate pool.

### Complex interactions between the malate and aspartate pools that concentrate CO_2_ into the BSC

The labeling kinetics of the C4 positions of malate and aspartate raised several questions: why in a predominantly NADP-ME-type plant does ^13^C enrichment initially rise faster in the C4 position of aspartate than that of malate (Figure 5A)? Why do rather similar amounts of ^13^C accumulate in the C4 positions of aspartate and malate at the earliest times in the labeling kinetic (Figure 5B)? Also, why is there a decline in labeling of the C4 position of aspartate relative to that of malate between the earliest times (5–10 s) and 60 s (Figure 5C)? Faster initial labeling of aspartate was seen in independent experiments in ML and LL and, with hindsight, is discernible in the data set of Arrivault et al. (2017). There may be several explanations for these unexpected observations.

First, the C4 position in aspartate may turn over more quickly than that of malate. This would be a direct consequence of the small pool size of aspartate, compared to malate (Figure 2A). Further, Arrivault et al. (2017) noted for plants illuminated at 480 µmol photons m^−2^ s^−1^ that the concentration gradient between the MC and BSC was equivalent to about 80% of the total aspartate pool but only 40%–50% of the active malate pool. This implies that, for a given total pool size, diffusion to the BSC may be faster for aspartate than malate.

Second, the labeling kinetics of the C4 position of malate might be complicated by release of unlabeled CO_2_ into the C_BSC_ by photorespiration and mitochondrial respiration. The reaction catalyzed by PEPCK is strictly irreversible but the reaction catalyzed by NADP-ME is closer to equilibrium (Bräutigam et al., 2018; Zhao et al., 2022), making it possible that at early time points some unlabeled CO_2_ is exchanged back into the C4 position of malate in the BSC. This would require quite high fluxes of unlabeled CO_2_ into the C_BSC_ and is unlikely to be a major factor in ML but may contribute in LL when influxes from photorespiration and mitochondrial respiration are probably higher relative to the influx of ^13^CO_2_ from CCMs (see above). The difference in labeling kinetics between the C4 positions of aspartate and malate was, however, large or even larger in ML than in LL (Figure 5, A, B, and D). This explanation therefore is unlikely to be a major factor.

Third, we have defined the active pool of malate as the amount of ^13^C in malate when labeling plateaus after a long pulse. However, it is possible that there are sub-pools; for example, one sub-pool that is directly involved in the CCM and one or more sub-pools that are partly and slowly equilibrated with the first sub-pool but with lower ^13^C enrichment at early time points. The finding (Supplemental Figure S9C) that ^13^C enrichment in C1–3 position of malate rises more slowly than enrichment in pyruvate, which lies downstream of NADP-ME, is consistent with the malate active pool containing sub-pools with slightly differing labeling characteristics. This might speculatively involve pools in different subcellular compartments, for example, in different subcellular compartments, or pools in other cell types than the MC and BSC.

Comparison of the labeling kinetics of malate and fumarate also pointed to the need for caution in interpreting malate labeling kinetics, especially in ML. Malate and fumarate are interconverted in a reversible reaction catalyzed by fumarase in the mitochondria. This is likely to occur both in the MC and in the BSC. This reaction was probably a major source of the label in fumarate, given that fumarate labeled much more quickly than other tricarboxylic acid intermediates like 2OG and succinate (Figures 1 and 4, F; Supplemental Figure S6F) and, like the active malate pool, had faster estimating labeling of the C4 than the C1–3 positions (Supplemental Figure S8). However, whereas in LL the labeling kinetics of the C4 and C1–3 positions of the active malate pool resembled those estimated for the corresponding positions in fumarate (Supplemental Figure S7, A and B), in ML labeling was faster for malate than for fumarate (Supplemental Figure S7, C and D). This could point to the presence of rapid-labeling and slow-labeling sub-pools of malate in ML, with the latter being closer to isotopic equilibrium with fumarate. An alternative explanation would be that isotopic equilibration between malate and fumarate is less complete in ML than in LL This is likely because the rate of label equilibration will be constrained by fumarase activity and speed of exchange between the cytosol and mitochondria, and this constraint may be more marked when photosynthetic flux is higher. Incidentally, because fumarate is a symmetrical molecule, ^13^C will be randomized between the C4 and C1 positions of the malate molecules that are formed from fumarate. This is, however, unlikely to lead to a major decrease in enrichment in the C4 position of malate because the fumarate pool is 1,000-fold smaller than the active malate pool.

Overall, these considerations also indicate that enrichment estimated at the C4 position of the active malate pool may underestimate enrichment in the pool that is utilized by NADP-ME. It should be noted that Hatch (1971) reported faster ^14^CO_2_ labeling of the C4 position of malate than the C4 position of aspartate in a pulse, and faster loss of label from malate than aspartate during a chase. This presumably reflects differing contributions of aspartate-based routes in maize grown and analyzed in different conditions. Overall, our analyses underline that the labeling kinetics of malate and aspartate, on their own, may not provide quantitative information about the contribution of NADP-ME and other decarboxylation pathways.

### Possible reasons why NADP-ME activity is restricted in LL

Nevertheless, our analyses do point to NADP-ME making a smaller contribution and aspartate-based decarboxylation routes making a larger contribution in LL than in ML. This shift may be due to several factors, operating both in the MC and in the BSC.

In the MC, the OAA formed by PEPC is reduced to malate by NADP-malate dehydrogenase in a reversible reaction in which, due to its thermodynamic equilibrium, there is a large excess of malate over OAA. At low irradiance, the NADPH/NADP^+^ ratio may fall, leading by mass action to lower malate and higher OAA levels. This will favor conversion of OAA to aspartate, providing one explanation why the aspartate pool is larger in LL than in ML. Aspartate increases in maize leaves of maize and other C_4_ species after a large decrease in irradiance (Doncaster et al., 1989) or darkening (Leegood and Furbank, 1984; Usuda, 1985) and is also higher in the dark than in the light in leaves of C_3_ plants (Igarashi et al., 2006; Ishihara et al., 2015) even in the absence of a C_4_ cycle. These light-dependent changes are most plausibly explained by a change in redox state. A shift in partitioning of OAA toward aspartate and away from malate will promote generation of a concentration gradient and speed up diffusion of aspartate to the BSC, where it may drive flux at PEPCK or NAD-ME. Any such shift will also hamper the generation of a concentration gradient for malate and restrict the substrate supply for NADP-ME in LL.

In the BSC in LL, NADP-ME activity might be additionally restricted by lack of demand for its products. Hatch and Kagawa (1976) demonstrated that malate decarboxylation is totally light-dependent in isolated BSC from NADP-ME-type species, but is only partially light-dependent in isolated BSC from NAD-ME or PEPCK species. Several factors might contribute to this strong light-dependence of NADP-ME activity. First, in LL there will be decreased NADPH consumption because the CBC is restricted by a low supply of ATP or by incomplete activation of CBC enzymes (see Bräutigam et al., 2019). Second, in contrast to the other CCM metabolites, there is no intercellular concentration gradient for pyruvate (Stitt and Heldt, 1985a; Arrivault et al., 2017) implying that pyruvate movement depends on active transport, although the responsible transport protein(s) in maize still need to be identified. It is possible that removal of pyruvate from the BSC chloroplast stroma is less effective in LL, placing an additional restriction on decarboxylation by NADP-ME. In agreement, as irradiance is increased, overall pyruvate levels in maize leaves increase up to an intensity of about 500 µmol photons m^−2^ s^−1^ and then plateau (Usuda, 1987) or even decrease (Leegood and von Caemmerer, 1989), indicating that active transport of pyruvate is promoted at higher irradiance. Incidentally, restriction of NADP-ME provides a plausible explanation why the active malate pool is as large in LL as in ML. Confirmation of the ideas in this and the previous paragraph will require more information about NADPH and ATP levels in the MC and BSC, and information about the activation state of CBC enzymes in low irradiance. It will also be interesting to investigate the irradiance-dependence of the post-translational inactivation of maize NADP-ME by Ser^419^ phosphorylation (Bovdilova et al., 2019).

Factors related to pathway topology may also increase flux over PEPCK relative to NADP-ME in LL. In the canonical PEPCK pathway, PEP returns from the BSC to the MC. However, compared to other metabolites that are involved in intercellular shuttles, PEP levels are rather low and this may impose an upper limit on how rapidly it can move back to the MC. This restriction may have less impact in LL, when fluxes are lower. PEP is equilibrated with 3PGA via the reversible enolase and phosphoglycerate reactions. Due to their thermodynamic equilibrium, 3PGA levels are about 2.5-fold higher than PEP levels (Figure 2, also Leegood and von Caemmerer, 1989; Ubierna et al., 2013). 3PGA would therefore diffuse more rapidly than PEP to the MC, where it can be converted back to PEP. Indirect support for this idea is provided by our observation that PEP is closer to isotopic equilibrium with 3PGA in LL than in ML (see below for more discussion). It might be noted, however, that decarboxylation by PEPCK coupled to an aspartate/3PGA shuttle would compete with use of the available 3PGA concentration gradient (see Stitt and Heldt, 1985a; Arrivault et al., 2017) for transfer of energy from the MC to the BSC, and might further impair photosynthesis in LL.

The question arises, if there is an advantage in increasing the contribution of an aspartate-based route in LL. In principle, use of PEPCK might be advantageous in LL because this route has a lower energy requirement (see “Introduction”). However, the canonical PEPCK pathway was recently questioned and an alternative pathway proposed with conversion of PEP to pyruvate in the BSC, which would require more energy than a NADP-ME or NAD-ME route (Bräutigam et al., 2018). It is not known if this modified pathway operates in species where PEPCK is only a minor component. It has been argued that simultaneous operation of alternative decarboxylase routes decreases the size of the pools and concentration gradients that are required for individual metabolites (Furbank, 2011; Wang et al., 2014). However, in LL the size of the pools may be less of an issue than the ability to use them to generate concentration gradients (see below). It has also been suggested that multiple decarboxylation routes provide increased flexibility to deal with different light regimes by altering NADPH and ATP demand in the MC and BSC (Bellasio and Griffiths 2014b). On the other hand, the NADP-ME route is the only CCM that transfers energy from the MC to the BSC, which is beneficial because only a low proportion of total chlorophyll is present in the BSC compared to that in the MC (Wang et al., 2014). Transfer of energy from the MC to the BSC may be as important or even more important in LL than high light. Seen in this perspective, the increased contribution of an aspartate-based CCM in LL might be detrimental but occur nonetheless due to the impact of low energy conditions on allocation of OAA between malate and aspartate, and on the operation of different decarboxylases.

### Use of metabolite pools to drive intercellular movement is less efficient in LL

As already discussed in the previous sections, the pools of malate and aspartate are not exploited efficiently to drive intercellular shuttles in LL. Although less extreme, a similar picture also emerges for the 3-carbon metabolites that return from the BSC to the MC, as well as for metabolites that shuttle energy from the MC to the BSC. Whereas the rate of photosynthesis falls about three-fold between ML and LL, the combined active pools of pyruvate and alanine only fell by approximately 50% (Figure 3C) and those of 3PGA and triose-P by only approximately 20% (Figure 3E). Presumably, light energy is directly or indirectly required to create the concentration gradients that drive intercellular diffusion, and this occurs less effectively in LL than in ML.

In the energy shuttle, under LL the reduction of 3PGA to triose-P in the MC may be restricted leading to accumulation of 3PGA that hinders its diffusion from the BSC, and to a lower level of triose-P that restricts its diffusion back to the BSC. Usuda (1987) and Leegood and von Caemmerer (1989) observed a small increase in the 3PGA:triose-P ratio in the light range used in our study. In our study, the 3PGA:triose-P ratio was similar in ML and LL (1.4 and 1.5, respectively) indicating that additional factors are also involved. These might include less consumption of triose-P in the CBC, possibly linked to incomplete activation of FBPase and other CBC enzymes. The marked transient rise of FBP at irradiances below 500 µmol m^−2^ s^−1^ in the study of Leegood and von Caemmerer (1989) and tendential peak in Usuda (1987) are consistent with this idea.

In the CCM shuttle, any restriction of malate decarboxylation in LL (see last section) will lead to a back-up of malate in the BSC and slow down diffusion of malate from the MC to the BSC. Several factors might restrict pyruvate movement back from the BSC to the MC in LL. As already mentioned, intercellular movement of pyruvate includes active transport and this might be restricted in LL. As already mentioned, pyruvate levels are relatively high in low irradiance and plateau (Usuda, 1987) or even decrease at higher irradiance (Leegood and von Caemmerer, 1989), which is consistent with the idea that active transport of pyruvate is restricted in LL and becomes increasingly effective as irradiance increases. Incidentally, any restriction on export of pyruvate for the BSC chloroplast might contribute to the restriction of flux over NADP-ME in LL conditions. The high pyruvate in low irradiance might also reflect incomplete activation of PPDK. In addition, as discussed later, back-leakage of CO_2_ may increase in LL. This would result in less efficient use of the intercellular pools of CCM intermediates in the sense that excess flux would be needed in the CCM to support a given net rate of CO_2_ transfer to the BSC.

### Photorespiration is faster in LL than in ML

Our results provide two lines of evidence that photorespiration is faster in LL than in ML, not only relative to the rate of photosynthesis but also in absolute terms. First, the pools of glycine, serine, and especially glycerate were larger in LL than in ML (Figure 2E; Supplemental Figure S7E). Second, based on ^13^C labeling kinetics, minimum flux to 2PG was estimated to be 0.89 and 1.84 nmol C g^−1^ FW s^−1^ in ML and LL, respectively. This is equivalent to a rate of RuBP oxygenation of about 0.45% and 0.92% of net CO_2_ fixation in ML and LL, respectively (Table 1).

The increased rate of photorespiration in LL implies that C_BSC_ is substantially lower in LL than in ML. A decrease of C_BSC_ in low irradiance was previously shown by direct measurements in the PEPCK subtype *Urochloa panicoides* (Furbank and Hatch, 1987) and was predicted for several C_4_ species from Δ^13^C fractionation data (Henderson et al. 1992; Tazoe et al. 2008; Kromdijk et al. 2010; Ubierna et al. 2013). This might serve to balance CO_2_ concentration by the CCM with slower CO_2_ utilization in the CBC. However, we found a two-fold higher absolute rate of photorespiration in LL compared to ML, and a two-fold increase in photorespiration relative to CO_2_ assimilation. These observations imply that C_BSC_ falls so far in LL that it negatively impacts CBC operation. They also add to the evidence that operation of the CO_2_ shuttle is impaired in LL conditions.

Earlier studies, mainly of the response of CO_2_ assimilation to low O_2_, estimated that photorespiration was 2%–7% of the rate of CO_2_ assimilation in C_4_ species (Volk and Jackson, 1972; de Veau and Burris, 1989; Laisk and Edwards, 1998). Even higher rates were reported immediately after transfer to LL (Henderson et al., 1992; Kromdijk et al., 2010). In our LL treatment plants were allowed to acclimate for several hours before starting the experiment. Our rates are nevertheless at the lower range of what has been previously reported.

### Back-leakage of CO_2_ from the BSC to the MC

CO_2_ back-leakage, Φ, is defined as the rate of CO_2_ movement back from the BSC to the MC relative to the rate at which HCO_3_ is assimilated by PEPC and moved by the CCM to the BSC. Φ usually lies in the range of 0.2–0.3 (Hatch et al., 1995; Kromdijk et al., 2014) and may be higher in LL (Evans et al., 1986; Ubierna et al., 2013; Bellasio and Griffiths, 2014a). Some of the CO_2_ leaking from the BSC to the MC may derive from photorespiration or from mitochondrial respiration in the BSC, rather than C that is assimilated by PEPC and transferred to the BSC (Kromdijk et al., 2010, 2014; Bellasio and Griffiths, 2014a). This will result in higher Φ in LL unless flux at PEPC is downregulated strongly enough to fully compensate for the larger contribution of internally released CO_2_.

Current methods for estimating Φ are complex and require many assumptions (see Ubierna et al., 2013; Kromdijk et al., 2014 and “Introduction”). We asked whether the fluxes estimated in Table 1 provide information about the extent of back-leakage. In ML, estimated flux at PEPC and flux at Rubisco were of the same order (121.3 ± 51.3 nmol ^13^C g^−1^ FW s^−1^ for PEPC compared to flux at Rubisco of 122.9 ± 8.2 nmol CO_2_ g^−1^ FW s^−1^ from gas exchange, and 91.3–205.7 nmol C g^−1^ FW s^−1^ from analysis of the ^13^C labeling kinetics (as discussed above, the estimates in ML have larger errors, and the very high rate based on the rather low enrichment estimated for the overall malate pool may be an overestimate). In LL, estimated flux at PEPC exceeded flux at Rubisco (62.8 ± 13.3 nmol ^13^C g^−1^ FW s^−1^ for PEPC, compared to flux at Rubisco of 45.5 ± 3.9 nmol CO_2_ g^−1^ FW s^−1^ from gas exchange and 31.3–38.7 nmol C g^−1^ FW s^−1^ from analysis of ^13^C labeling kinetics). This discrepancy largely reflects the two-fold decrease in estimated flux at PEPC between ML and LL, whereas the rate of photosynthesis decreased by almost three-fold. This analysis points to a larger excess of CO_2_ influx to the BSC in LL than in ML, providing independent support for the idea that Φ increases in LL.

As outlined above, it has been proposed that increased flux of unlabeled C into C_BSC_ can contribute to an increase in Φ in low irradiance. This idea is supported by the analysis of our labeling kinetics in Figure 5, D and E (see above for discussion). Furthermore, our flux estimates confirm that the absolute rate of CO_2_ release by photorespiration is higher in LL than in ML (see Table 1 and previous section). However, at least in the conditions of our study, photorespiration does not make a substantial contribution to total flux of CO_2_ into C_BSC_ (1.84 compared to 62.8 and 1.3 nmol C g^−1^ FW s^−1^ from the CCM and mitochondrial respiration, see above). This points to a contribution from mitochondrial respiration, which would be in agreement with the observation that Φ rose in LL even under 2% O_2_ (Kromdijk et al., 2010).

There is an apparent contradiction between the increase of Φ in LL, and the decrease of C_BSC_ and increased rate of photorespiration in LL (see also Bellasio and Griffiths, 2014a; Sage, 2014). Although Sage (2014) suggested that this contradiction could be explained by faster photorespiration in LL, in our conditions the estimated rate of recycling of CO_2_ by photorespiration is too slow to have a major impact on Φ. This raises the question whether further factors lead to increased back-leakage in low irradiance. One possible explanation might be changes in plasmodesmatal conductivity (Sowinski et al., 2008). Another might be the site of CO_2_ release in the BSC (von Caemmerer and Furbank, 2003), with a higher probability of back-leakage when CO_2_ is released in the mitochondria by NAD-ME or in the cytosol by PEPCK than when CO_2_ is released by NADP-ME in the plastid in the immediate vicinity of Rubisco. Furbank and Hatch (1987) pointed out that the inorganic C in the BSC is probably mainly present as CO_2_, and that back-leakage would increase if more were converted to HCO_3_^–^^.^ There may be more conversion if diffusional distances and half-times are increased by releasing CO_2_ in the mitochondria or cytosol. It has already been discussed that sub-pools of CO_2_ in different subcellular compartments might affect interpretation of Δ^13^C discrimination (Kromdijk et al., 2014). An increased probability of back-leakage of CO_2_ released by NAD-ME or PEPCK might also explain the high initial rates of ^13^C incorporation into aspartate and rapid movement of ^13^C through aspartate compared to malate at early times in the labeling kinetic (see above).

### Rapid C exchange between CBC and CCM allows rapid balancing of fluxes in both LL and ML

Irrespective of the light intensity, C_4_ photosynthesis requires coordination of fluxes in the CBC and CCM (Furbank and Hatch, 1987; Jenkins et al., 1989; von Caemmerer, 2000; Sage, 2014). As already mentioned, these fluxes will be partly determined by concentrations of metabolites. In steady-state conditions, the CCM and CBC operate separately, with no shared metabolites except for CO_2_ in the BSC, which is generated by the CCM and assimilated by the CBC. However, they are linked by two reversible reactions catalyzed by phosphoglycerate mutase and enolase, which interconvert 3PGA and PEP. Indeed, the 3PGA:PEP ratio in maize leaves is close to the expected equilibrium constant (Leegood and von Caemmerer, 1989; Ubierna et al., 2013; see also Figure 2). These reversible reactions could facilitate carbon exchange between the CBC and the CCM and play an important role in balancing flux in these two interdependent pathways (Leegood and von Caemmerer, 1988, 1989; von Caemmerer, 2000; Stitt and Zhu, 2014).

Our flux estimates reveal that C is exchanged between the CBC and the CCM at rates that would move 10% of the C in the CBC to the CO_2_ shuttle in about 25 s, or 10% of the C in the CO_2_ shuttle to the CBC in about 36 s in ML, with slightly longer times being needed in LL (see Supplemental Text S3). This capacity to rapidly move C between the CBC and CCM will allow fast responses to rebalance C_4_ photosynthesis after sudden changes in the environment.

### Large pools of photorespiratory intermediates in low irradiance may provide a C reserve during a transition to higher irradiance

Adjustment of C_4_ photosynthesis to changes in irradiance may be aided by transfer to C between external C pools and pools that are directly involved in photosynthesis (i.e. the CCM and CBC). The large pools of photorespiratory metabolites in LL (Figure 2E; Supplemental Figure S7E) provide a substantial C reservoir to build up CBC and CCM pools if irradiance were to increase. A similar scenario might also be relevant during recovery of photosynthesis after a period of stomatal closure. The pool of photorespiratory metabolites in LL is far larger than the increase in pool size of CBC or CO_2_ shuttle metabolites between LL and ML (see Figures 2 and 3; Supplemental Text S2).This accumulation might be supported by regulation of glycerate kinase that in C_4_ NADP-ME plants like maize, unlike most other species, is subject to light-dependent regulation by thioredoxin (Kleczkowski and Randall 1986; Bartsch et al., 2010). As glycerate kinase is located in the MC (Bräutigam et al., 2008; Pick et al., 2013), activation of glycerate kinase will generate 3PGA that can be reduced to triose-P in the MC and then move to the BSC to support build-up of metabolite pools. The NADPH and ATP that could be transferred as triose-P to the BSC exceeds that transferred by a 3PGA/triose-P exchange in 10 s of photosynthesis in ML, providing a substantial one-time boost in energy transfer to the BSC (see Supplemental Text S4).

In conclusion, photosynthesis in the C_4_ species maize is compromised in low irradiance, not only due to the energy demand of the CCM but also because operation of the CCM is impaired, photorespiration rates are higher and back-leakage of CO_2_ is increased in low irradiance. At the same time, there is considerable flexibility in C_4_ metabolism allowing rapid adjustment of pools sizes and fluxes in the CO_2_ shuttle, the energy shuttle, and the CBC. Future studies are needed in other species, and also in leaf material that has become acclimated to low irradiance. A better mechanistic understanding of the impact of low irradiance on C_4_ photosynthesis has implications for improving the effectiveness of C_4_ photosynthesis in the field after the canopy has closed and leaves experience low irradiance (Sales et al*.*, 2021), for understanding which factors decrease the efficiency of C_4_ photosynthesis in fluctuating light (Kubasek et al., 2013; Li et al., 2021), as well as for ecological questions related to why C_4_ photosynthesis did not evolve often in shrubs or trees with bushy canopies (Sage, 2014).

## Materials and methods

### Chemicals

Isotopically labeled carbon dioxide (^13^CO_2_, 99.0 atom % ^13^C) was from Sigma-Aldrich (St Louis, MO, USA). Nitrogen (N_2_), oxygen (O_2_), and unlabeled carbon dioxide (CO_2_) were from Air Liquide (Germany). Chemicals were from Sigma-Aldrich, Roche (Basel, Switzerland), or Merck (Darmstadt, Germany).

### Plant growth

Maize (*Z.mays* L. cv. B73) seeds were germinated in darkness in petri dishes on moistened filter paper (3 days, 28°C), transferred to soil in 10-cm diameter pots, grown for 5 days under 16/8-h day/night cycles (irradiance 105 µmol photons m^−2^ s^−1^, 22°C/18°C, 70% relative humidity) and then under 14/10-h day/night cycles (irradiance 550 µmol photons m^−2^ s^−1^, 29°C/22°C, 65% relative humidity). This growth irradiance is a moderate/medium irradiance and is referred as ML through this study. The fourth fully elongated leaves of 3-week-old plants were used for ^13^CO_2_ labeling and gas exchange. The plants used to perform experiments in LL were transferred before the offset of lights the day prior to the experiments to a covered area in the growth chamber with an irradiance of 160 µmol photons m^−2^ s^−1^. Experiments in LL were done under this covered area.

### ^13^CO_2_ labeling set-up and quenching procedure

^13^CO_2_ labeling was performed with the labeling set-up essentially as described in Ermakova et al. (2021). During experiments, both unlabeled and ^13^C-labeled gas mixtures [79% (v/v) N_2_, 21% (v/v) O_2_ and 400 ppm ^12^CO_2_/^13^CO_2_] were continuously running (flow rate of 10 L min^−1^), directed either to the labeling chamber or to a CO_2_ trap (soda lime) to capture ^12^CO_2_/^13^CO_2_ (Supplemental Figure S1, A and B). A stainless steel 1-piece 40 series four-way ball valve (Swagelok, Solon, OH, USA) was used to control which gas mixture entered the labeling chamber. The custom-designed labeling chamber was made of two transparent Plexiglas plates (Supplemental Figure S1C) and absorbed about 10% of the incident light. Inspection of light saturation responses for maize grown in the same conditions as the current study reveals (Arrivault et al., 2019) that the resulting decrease in photosynthetic rate would be within the standard deviation of the current measurements.

Labeling started a minimum of 3 h after the start of the light period to ensure metabolic steady state was reached and was performed over the course of 4 days in ML and also 4 days in LL, 1 week later using a separately grown batch of plants. Two fourth fully expanded leaves from two different plants were placed in the labeling chamber, the chamber fastened by clamps (Supplemental Figure S1D); and supplied with a continuous flow of unlabeled gas mixture (Supplemental Figure S1A). After 1 min, the unlabeled gas mixture was switched to the ^13^C-labeled gas mixture using the four-way valve (Supplemental Figure S1B). For labeling at ML, ^13^CO_2_ pulses were performed for duration of 5, 10, 15, 20, 30, 50 s and 1, 3, 5, 10, 20, 40, or 60 min. For labeling at LL, pulse durations were 10, 30 s and 1, 3, 10, 40, 60 min, and 2 or 4 h. In both cases, unlabeled samples (*t* = 0) were collected after supplying the chamber 1 min with the unlabeled gas mixture. Pulses of different durations were performed in a random manner both within and between days. The temperature in the chamber and leaf temperature were both 30°C.

Plant material was rapidly quenched by pouring liquid N_2_ into the labeling chamber via a funnel (Supplemental Figure S1E). As soon as the plant material was quenched (Supplemental Figure S1F), the ^13^C-labeled gas mixture was re-directed to the CO_2_ trap by switching the four-way valve (Supplemental Figure S1A). The two ^13^CO_2_-labeled leaf sections were removed from the chamber (Supplemental Figure S1G), and separately stored at −80°C.

### Metabolite analyses and calculation of total pool size, enrichment, isotopomer distributions, positional enrichment, and ^13^C amounts

Maize material was ground to fine powder using a ball mill (Tesch, Haan, Germany) at liquid N_2_ temperature and stored at –80°C. Samples were analyzed by LC–MS/MS and GC–MS with authentic standards for accurate metabolite quantification as in Arrivault et al. (2017). The total amounts of PEP, pyruvate, and 3PGA were determined enzymatically in freshly prepared trichloroacetic acid extracts as described in Merlo et al. (1993) using a spectrophotometer (Shimadzu, Kyoto, Japan). The ^13^C enrichments, relative isotopomer distributions, active and inactive pools, positional ^13^C enrichments (C4 and C1–3 positions) of malate and aspartate, and ^13^C amounts (natom ^13^C equivalents g^−1^ FW) in metabolites were calculated as in Arrivault et al*.* (2017).

### Gas exchange

The net CO_2_ assimilation was measured with an open-flow infrared gas exchange analyzer system (LI-6400XT, LI-COR Inc., Lincoln, NE, USA; www.licor.com) equipped with an integrated fluorescence chamber head (LI-6400-40, 2 cm^2^ leaf chamber; LI-COR Inc., Lincoln, NE, USA). CO_2_ was kept at 400 µmol mol^−1^, leaf temperature at 29°C, and relative humidity at 65%. The photosynthetically active photon flux density (PPFD) inside the chamber was kept at 550 µmol m^−2^ s^−1^ (ML) or 160 µmol m^−2^ s^−1^ (LL). The amount of blue light was set to 10% PPFD to optimize stomatal aperture. The net CO_2_ assimilation was recorded every 6 s in two sets of measurements on the same set of plants. In one, measurements were performed in ML or LL 4 h after the onset of light in the growth chamber and with five plants per irradiance. In the other, these plants were switched from ML to LL or from LL to ML, and CO_2_ assimilation was measured for 800 s after 15 min in the new condition, by which time steady-state rates had been achieved. Means ± sd (µmol m^−2^ s^−1^ or nmol g^−1^ s^−1^ using leaf area of 0.005484 m^2^ g^−1^) were calculated averaging both measurements.

### Statistical analyses

One-way analysis of variance (ANOVA) was performed using SigmaPlot vers. 14.0 (Systat Software, Inc.). *k*-means (https://www.ncbi.nlm.nih.gov/pmc/articles/PMC8786664/-CR91) clustering was performed in R Studio vers. 1.2.5033, the number of clusters being estimated as in Mardia et al. (1979).

## Data availability

All data obtained for this study are presented within the Supplemental Data Sets.

## Supplemental data

The following materials are available in the online version of this article.

**Supplemental Text S1.** Summary of differences between the three C_4_ subtypes.

**Supplemental Text S2.** Time resolved labelling patterns reflect the topology of C_4_ photosynthesis.

**Supplemental Text S3.** Rapid C exchange between CBC and CCM in both LL and ML.

**Supplemental Text S4.** Large pools of photorespiration intermediate in low irradiance may provide a C reserve during transitions to higher irradiance.

**Supplemental Figure S1.** Set-up for providing short ^13^CO_2_ pulses and quenching procedures.

**Supplemental Figure S2.** Comparison of metabolite pool sizes at different days and times during the harvesting period.

**Supplemental Figure S3.** Overview of measured ^13^C enrichment kinetics by *k*-means.

**Supplemental Figure S4.** Detailed labelling kinetics of metabolites presenting two or more pools, of which only one is labelled by newly fixed ^13^C.

**Supplemental Figure S5**. Regression plots of ^13^C amounts and positional ^13^C amounts in malate and aspartate calculated with two approaches.

**Supplemental Figure S6.** ^13^C enrichments (%) of individual metabolites in medium and low light.

**Supplemental Figure S7.** ^13^C amounts (natom ^13^C equivalents g^−1^ FW) of individual metabolites in medium and low light.

**Supplemental Figure S8.** Comparison of the positional labelling kinetics of fumarate and malate.

**Supplemental Figure S9.** Labelling kinetics of PEP, C1-3 positions of malate and aspartate, pyruvate and alanine.

**Supplemental Figure S10.** Labelling kinetics of 3PGA and PEP.

**Supplemental Table S1.** Information about analyzed metabolites.

**Supplemental Table S2.** Metabolite active pool sizes in medium and low light.

**Supplemental Table S3.** ^13^C enrichment kinetics for individual metabolites in medium and low light.

**Supplemental Table S4.** ^13^C amounts for individual metabolites in medium and low light.

**Supplemental Table S5.** Summed ^13^C amounts in the C4 and C1-3 positions of malate and aspartate, and summed ^13^C amounts in other sets of metabolites in medium and low light.

**Supplemental Table S6.** Estimation of fluxes based on leaf area through PEPC, the C4 positions of malate and aspartate, Rubisco, the C carriers in CO_2_ shuttle, and photorespiration in medium and low light.

**Supplemental Data Set S1.** Amounts of unlabelled form and ^13^C-isotopomers for each metabolite in ML.

**Supplemental Data Set S2.** Relative isotopomer abundance (%) in ML.

**Supplemental Data Set S3.** Amounts of unlabelled form and ^13^C-isotopomers for each metabolite in LL.

**Supplemental Data Set S4.** Relative isotopomer abundance (%) in LL.

**Supplemental Data Set S5.** Metabolic content in ML and LL.

**Supplemental Data Set S6.** ^13^C enrichment (%) of metabolites in ML and LL.

**Supplemental Data Set S7.** Estimation of ^13^C amounts in the CO_2_ shuttle, CBC and first intermediates of starch and sugar intermediates, photorespiratory intermediates and additional metabolites in ML.

**Supplemental Data Set S8.** Estimation of ^13^C amounts in the CO_2_ shuttle, CBC and first intermediates of starch and sugar intermediates, photorespiratory intermediates and additional metabolites in LL.

**Supplemental Data Set S9.** Calculation of carbon-dependent ^13^C enrichment in the C4 position and in C1-3 positions of malate, aspartate and fumarate in ML and LL.

**Supplemental Data Set S10.** ^13^C amounts in various metabolic sectors in ML and LL.

**Supplemental Data Set S11.** Additional data used for estimation of fluxes (Table 1).

## Funding

This work was financially supported by the Max Planck Society (H.I., M.G., L.R.D.), by C_4_ Rice Project grants from Bill & Melinda Gates Foundation to the University of Oxford (2015–2019; OPP1129902; 2019–2024 INV-002870 (H.I., M.S., S.A.), and the German Federal Ministry of Education and Research (BMBF grant 031B0205C to D.B.M., A.R.F., M.S., S.A.).

*Conflict of interest statement*. None declared.

## Supplementary Material

kiac306_Supplementary_Data

## Notes

D.B.M., A.R.F., M.S., and S.A. conceived and planned the experiments. D.B.M. grew plants and performed ^13^C labeling with help from H.I. and L.R.S. D.B.M., M.G., and S.A. extracted the samples for downstream analyses. M.G. and S.A. processed and analyzed LC-MS/MS and enzymatically assayed samples. D.B.M. processed and analyzed GC–MS samples. H.I. performed statistical analyses. D.B.M., A.R.F., M.S., and S.A. wrote the manuscript. A.R.F. and M.S. supervised the project. All authors approved the final version.

The author responsible for distribution of materials integral to the findings presented in this article in accordance with the policy described in the Instructions for Authors (https://academic.oup.com/plphys/pages/general-instructions) is Stéphanie Arrivault (arrivault@mpimp-golm.mpg.de).
