# Supplementary material for: 13CO2 labeling kinetics in maize reveal impaired efficiency of C4 photosynthesis under low irradiance
Source: Plant Physiol. 2022 Jun 25;190(1):280–304. doi: 10.1093/plphys/kiac306 (PMC9434203; doi:10.1093/plphys/kiac306)
Supplement: kiac306_Supplementary_Data [file kiac306_supplementary_data.zip › Medeiros et al_ Supplemental Texts Figures Tables.pdf]

Supplemental Data. Medeiros *et al*. (2022) $^{13}CO_2$ Labelling kinetics in maize reveal impaired efficiency of C_4 photosynthesis under low irradiance.

Supplemental Text S1. Summary of differences between the three C_4 subtypes.

In the NADP-ME subtype, the oxaloacetate (OAA) that is produced by PEPC is reduced to malate in the MC. Malate diffuses to the BSC where it is decarboxylated in the chloroplast to release CO_2 and NADPH, which are used in the CBC, and pyruvate that moves back to the MC. This subtype often exhibits partial or near-complete loss of photosystem II (PSII) in the BSC chloroplasts, linked with the operation of a intercellular energy-transfecting shuttle in which up to half of the 3-phosphoglycerate (3PGA) formed in the BSC moves to the MC where it is reduced and moves back as triose-phosphate (triose-P) to the BSC (Woo *et al*., 1970; Andersen *et al*., 1972; Leegood, 1985; Stitt and Heldt 1985a; Munekage, 2016). In the NAD-ME subtype, OAA is converted in the MC to aspartate, which diffuses to the BSC where it is converted back to OAA and reduced to malate in the mitochondria using the NADH generated by NAD-ME (Bräutigam *et al.*, 2018). The pyruvate formed by NAD-ME and the amino group from aspartate move back to the MC as alanine, which is then converted to pyruvate. In both NADP-ME and NAD-ME subtypes, pyruvate is converted to PEP by pyruvate phosphate dikinase (PPDK) in the MC chloroplasts, consuming two molecules of ATP (Bräutigam *et al*., 2018). In the PEPCK subtype, OAA is converted to aspartate, which diffuses to the BSC where it is converted back to OAA and decarboxylated in the cytosol in an ATP-dependent manner leading to formation of PEP. The energy demand of the PEPCK pathway is less than that of the NADP-ME or NAD-ME pathway; per CO_2 transferred, PEPCK-based transfer will consume one ATP in the BSC whereas NADP-ME-based transfer consumes two ATP equivalents and one NADPH in the MC and releases one NADPH in the BSC (see Kromdijk *et al*., 2014). However, there are open questions about how phosphate stoichiometry is maintained between the MC and BSC, and whether PEP concentrations are high enough to drive diffusion back to the MC. Bräutigam *et al*. (2018) have proposed that predominantly PEPCK-based CCM species use enhanced PEP phosphatase activity in the BSC to convert PEP to pyruvate, which moves to the MC where it is converted back to PEP by PPDK.

Woo KC, Anderson JM, Boardman NK, Downton WJS, Osmond CB, Thorne SW (1970) Deficient photosystem II in agranal bundle sheath chloroplasts of C_4 plants. PNAS **67**: 18-25

Andersen KS, Bain JM, Bishop DG, Smillie RM (1972) Photosystem II activity in agranal bundle sheath chloroplasts from *Zea mays*. Plant Physiol **49**: 461-466

Munekage NY (2016) Light harvesting and chloroplast electron transport in NADP-malic enzyme type C_4 plants. Curr Opin iPlant Biol **31**: 9-15

Supplemental Text S2. Time resolved labelling patterns reflect the topology of C_4 photosynthesis

Our results confirm and extend conclusions about the operation of C_4 photosynthesis reached in earlier $^{14}CO_2$ (Hatch, 2002; Furbank, 2016) and $^{13}CO_2$ labelling studies in maize (Weismann *et al*., 2016; Arrivault *et al*., 2017). There is (i) extremely fast labelling of the C4 positions of malate, (ii) slightly slower and synchronous labelling of all CBC intermediates including 3PGA and triose-P, which are present at high levels and are involved in a shuttle that transfers NADPH and ATP from the MC to the BSC, and (iii) a delay before label moves into PEP, and the C1-3 positions of malate and pyruvate. Although there is substantial C exchange between CBC intermediates and metabolites involved in the CO_2 shuttle, this does not result in a rapid rise in enrichment of ^{13}C in PEP because C fluxes into PEP are dominated by the CO_2 shuttle, which at early labelling times recycles unlabelled C into PEP. These labelling patterns reflect the known topology of the NADP-ME subtype of C_4 photosynthesis. Our results (iv) confirm rapid labelling of aspartate, with similar or even faster kinetics than those of the C4 position of malate, and labelling of alanine with similar kinetics to those of PEP, the C1-3 positions of malate and aspartate, and pyruvate. These observations show that maize operates a minor component involving NAD-ME or PEPCK (see also Weissmann *et al*., 2016, Arrivault *et al*., 2017, Arp *et al.,* 2021). Furthermore, our results (v) confirm the occurrence of photorespiration, with progressive labelling of 2PG, glycine, serine and glycerate.

The relative amount of C in the backbone of metabolites in the CO_2 shuttle and in 3PGA and triose-P is consistent with the topology of C_4 photosynthesis in maize. Combined C in 3PGA and triose-P was equivalent to about 39 and 35% of the combined C in backbone of the CO_2 shuttle metabolites in ML and LL, respectively (Fig. 3). Making the simplifying assumptions that all decarboxylation occurs via NADP-ME, that no NADPH is delivered by photosystem II (PSII) in the BSC and that the main product of photosynthesis is sucrose that is formed in the MC (Furbank *et al*., 1985; Stitt and Heldt, 1985b; Furbank and Kelly, 2021), 50% of the 3PGA formed by Rubisco will need to move to the MC and at least 33% will need to return as triose-P to the BSC. The value will be higher if substantial amounts of the fixed C are used to make end-products in the BSC like starch, and if NAD-ME or PEPCK contribute to the CCM because these decarboxylation routes do not supply NADPH to the BSC. The value will be lower if there is photorespiration or if there is back-leakage of CO_2 from the BSC to the MC (see Introduction). The relative amounts of C in the CO_2 shuttle intermediates and in the energy shuttle intermediates are roughly proportionate to required fluxes, as expected if intercellular movement is largely by diffusion, whose rate is constrained by the size of the pools that are available to generate concentration gradients. Incidentally, the larger pool of 3PGA compared to triose-P (42% and 53% larger, respectively, in ML than LL, Supplemental Table S2) is consistent with the stoichiometry outlined above.

Furbank RT, Foyer C, Stitt, M (1985) The localization of sucrose synthesis in maize leaves. Planta **164**: 172-178
Furbank RT (2016) Walking the C_4 pathway: past, present, and future. J Exp Bot **67**: 4057-4066
Furbank RT, Kelly S (2021) Finding the C_4 sweet spot: cellular compartmentation of carbohydrate metabolism in C_4 photosynthesis. J Exp Bot **72**: 6018-6026

Supplemental Text S3. Rapid C exchange between CBC and CCM in both LL and ML

To function efficiently, C_4 photosynthesis requires close coordination of the CBC and CCM (Furbank and Hatch, 1987; Jenkins *et al*., 1989; von Caemmerer, 2000; Sage 2014). This is necessary across a wide range of steady state conditions. Furthermore, in fluctuating environment, photosynthetic efficiency will be decreased unless this balance can be re-established. Indeed, it appears that photosynthetic efficiency under fluctuating light is particularly affected in C_4 species (Kubasek *et al*., 2013).

As discussed in the main text, flux in the CCM and CBC will be partly determined by concentrations of metabolites. In steady state conditions, the CCM and CBC operate separately, with no shared metabolites except for CO_2 in the BSC, which is generated by the CCM and assimilated by the CBC. However, they are linked by two reversible reactions catalysed by phosphoglycerate mutase and enolase, which interconvert 3PGA and PEP. Indeed, the 3PGA:PEP ratio in maize leaves is rather constant and close to the expected equilibrium constant across a wide range of irradiance and CO_2 concentration (Leegood and von Caemmerer, 1989; Ubierna *et al*., 2013; see also Fig. 2). These reversible reactions could facilitate carbon exchange between the CBC and the CCM, and play an important role in balancing flux in these two interdependent pathways, especially in fluctuating conditions (Leegood and von Caemmerer, 1988, 1989; von Caemmerer, 2000; Stitt and Zhu, 2014).

Our flux estimates reveal that C is exchanged between the CBC and the CCM at rates of 22.4 and 13.6 nmol C g^{-1} FW s^{-1} in ML and LL, respectively (Table 1), equivalent to about ~18% and ~22% of the rate of CO_2 fixation measured in gas exchange in ML and LL. In steady state, this involves C exchange with no net flux. However, if the relation between 3PGA and PEP levels were dramatically changed and flux became temporarily unidirectional, these rates would suffice to move 10% of the C in the CBC to the CO_2 shuttle in about 25 s, or 10% of the C in the CO_2 shuttle to the CBC in ~36 s in ML, with slightly longer times being needed in LL (estimated for a total pool size in the CO_2 shuttle and the CBC (including 3PGA and triose-P) of about 8200 and 6250 nmol ^{13}C g^{-1} FW in ML, and 7900 and 5200 nmol ^{13}C g^{-1} FW in LL, see Figs. 3D and 3E-F). This capacity to rapidly move C between the CBC and CCM will allow fast responses to rebalance C_4 photosynthesis after sudden changes in the environment.

The almost 2-fold higher rate of C exchange relative to C fixation in LL compared to ML is consistent with equilibration of PEP and 3PGA being restricted by the enzymatic capacity of phosphoglycerate mutase and enolase; assuming that there are no major changes in the enzymatic capacity during the few hours acclimation in LL, enzymatic capacity will be higher relative to the rate of photosynthesis in LL than in ML. The absolute rate of exchange was ~64% higher in ML compared to LL. This is partly but not fully explained by slightly higher levels of 3PGA and PEP in ML (20 and 17%, see Fig. 2, Supplemental Table S2), which will lead to higher substrate saturation and slightly faster catalysis per unit of enzyme capacity in ML than in LL.

A contribution of PEPCK to decarboxylation adds a further facet to interactions between the CBC and the CCM. Decarboxylation by PEPCK produces PEP, which is likely to equilibrate with 3PGA in the BSC. As discussed in the main text, due to the equilibrium constants of enolase and phosphoglycerate mutase there will be a larger absolute concentration gradient for 3PGA than PEP. Much of the PEP produced by PEPCK may therefore return as 3PGA to the MC where it is converted to PEP. The consequence would be that when PEPCK makes a larger contribution to the CCM, fluxes in the CBC and the CCM will become more closely connected. A larger relative contribution from PEPCK would provide a further explanation why, relative to the rate of CO_2 fixation, ^{13}C is transferred more rapidly from the CBC to the CCM in LL than in ML (see above) but might also be one of the factors that decreases photosynthetic efficiency in LL (see main text).

Kubásek J, Urban O, Santrucek J (2013) C_4 plants use fluctuating light less efficiently than do C_3 plants: a study of growth, photosynthesis and carbon isotope discrimination. Physiol Plant **149**: 528-539

Supplemental Text S4. Large pools of photorespiration intermediate in low irradiance may provide a C reserve during transitions to higher irradiance

As already discussed by Leegood and von Caemmerer (1988, 1989) and Usuda (1985, 1987) the build-up of CBC and CCM intermediate pools in high light is probably driven not only by newly fixed C but also by inflow of C from other metabolite pools that act as a C reserve, Similarly, when light intensity falls, CBC and CCM pools may decline not only due to a transiently excess of end product synthesis over the rate of photosynthesis, but also by movement of C into reserve pools.

Our results show that photorespiration may provide an important C reserve during adjustment between different light intensities. The large pools of photorespiratory metabolites in LL (Fig. 2E, Supplemental Fig. S7E) provide a substantial C reservoir to build up CBC and CCM pools if irradiance were to increase. A similar scenario might also be relevant during recovery of photosynthesis after a period of stomatal closure.

In maize and most other C_4 plant species, photorespiration is distributed over two cell types, with all enzymes of the pathway being localized in BSC, with the exception of the glycerate transporter and glycerate kinase that are located in the MC chloroplasts (Bräutigam *et al*., 2008; Pick *et al*., 2013). Location of glycerate kinase in the MC means that the resulting 3PGA could be immediately converted to PEP and used by PEPC to build up pools in the CCM or could move to the BSC to build up CBC pools. In the latter case, NADP and ATP would also be transferred from the MC to the BSC (see also Kromdijk *et al*., 2014). The glycerate pool (about 1000 nmol g^{-1} FW or 1500 nmol ^{13}C g^{-1} FW) and the combined pool of photorespiratory metabolites (~1300 nmol g^{-1} FW or ~2000 nmol ^{13}C g^{-1} FW) in LL are far larger than the increase in pool size of the CBC intermediates (250 nmol g^{-1} FW or 1000 nmol ^{13}C g^{-1} FW) or CO_2 shuttle metabolites (340 nmol g^{-1} FW or 400 nmol ^{13}C g^{-1} FW) between LL and ML (see Figs. 2 and 3). The NADPH and ATP that could be transferred as triose-P to the BSC exceeds that transferred by a 3PGA/triose-P exchange in 10 s of photosynthesis in ML, providing a substantial one-time boost in energy transfer to the BSC that is independent of movement of 3PGA from the BSC to the MC.

Use of photorespiratory metabolites as a C reservoir requires regulation of the conversion of glycerate to 3PGA. The large accumulation of glycerate in LL (Fig. 2E, Supplemental Fig. S7E) is consistent with partial inhibition of glycerate kinase. In contrast to C_3-type glycerate kinase, maize glycerate kinase is redox-activated by thioredoxin (Kleczkowski and Randall, 1985; Kleczkowski and Randall, 1986) due to containing an unusual autoinhibitory domain (Bartch *et al*., 2010). This might restrict activity in low irradiance, favoring accumulation of glycerate in LL and promoting recycling when irradiance increases. In addition, maize glycerate kinase is competitively inhibited by 3PGA, indicating that its activity may be restricted when 3PGA reduction is restricted by low NADPH or ATP (Kleczkowski and Randall, 1988).

Kleczkowski LA, Randall DD (1985) Light and thiol activation of maize leaf glycerate kinase. The stimulating effect of reduced thioredoxins and ATP. Plant Physiol **79**: 274-277

Kleczkowski LA, Randall DD (1986) Thiol-dependent regulation of glycerate metabolism in leaf extracts. The role of glycerate kinase in C_4 plants. Plant Physiol **81**: 656-662

Kleczkowski LA, Randall DD (1988) Purification and characterization of D-glycerate-3-kinase from maize leaves. Planta **173**, 221-229

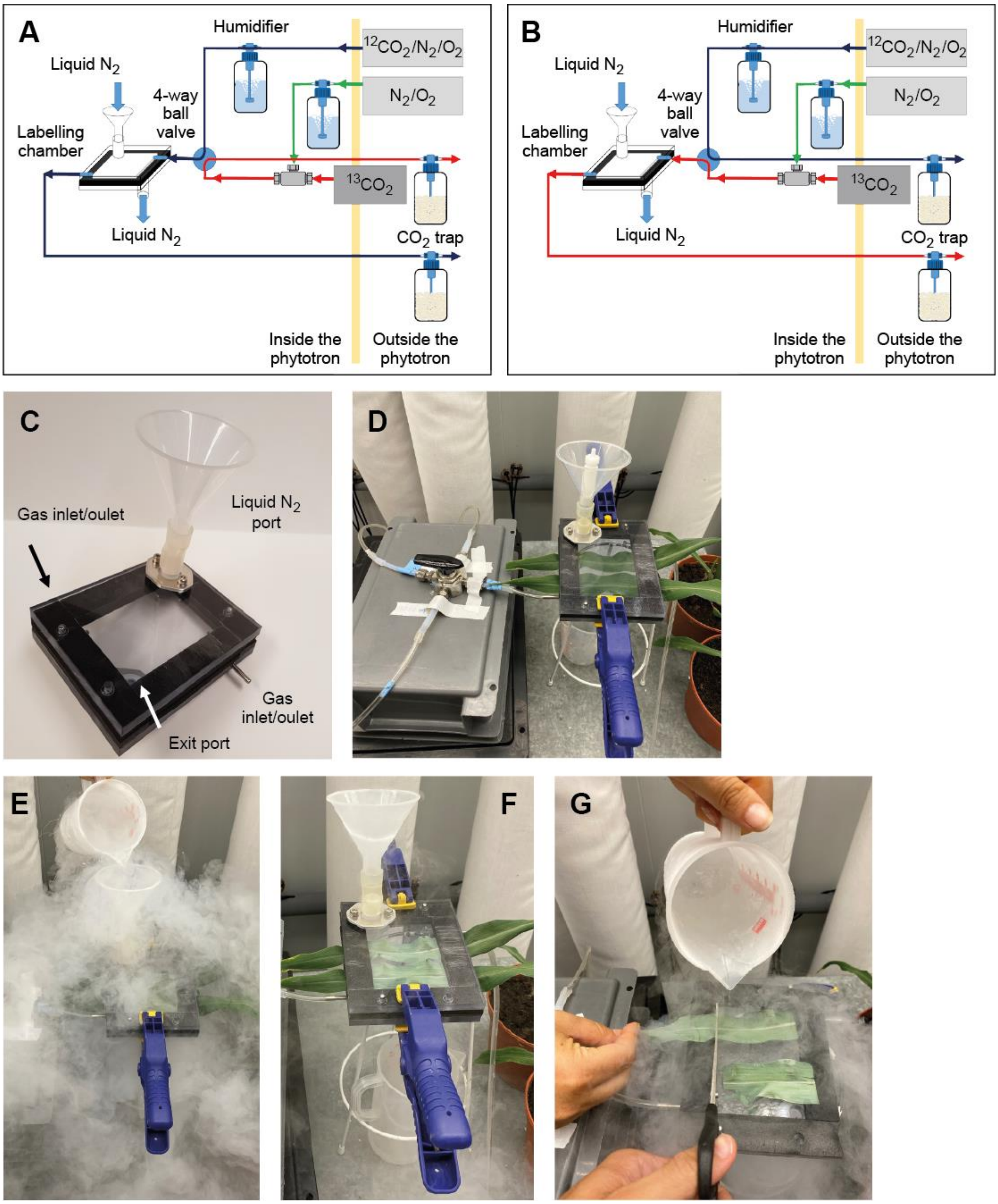

Supplemental Figure S1. Set-up for providing short $^{13}CO_2$ pulses and quenching procedures. (A and B) Schematic representation of the labelling apparatus and gas supplies. Artificial air mixtures were prepared from separate N_2, O_2 and CO_2 gas cylinders using stationary WMR 4008 gas mixers (Westphal Mess-und Regeltechnik GmbH, Ottobrunn, Germany). One mixer was used to prepare an unlabelled gas mixture containing 79% (v/v) N_2 and 21% (v/v) O_2 and 400 ppm $^{12}CO_2$. The second was used to prepare a CO_2-free gas mixture containing 79% (v/v) N_2 and 21% (v/v) O_2. A GC40 mass flow controller (Brooks Instrument, Hatfield, PA, USA) connected to a 10-L cylinder of $^{13}CO_2$ was used to supply $^{13}CO_2$ which was mixed with the humidified CO_2-free air mixture, using a custom-made Tee gas mixer (Bronkhorst, Ruurlo, the Netherlands), to give a final concentration of 400 ppm $^{13}CO_2$. For both gas mixtures, the flow rate was 10 L min^{-1} and the gas mixture was bubbled through purified water at 30°C

water to give a relative humidity of 65%. Gas mixtures were continuously running, either directed to the labelling chamber or to CO2 trap. A 4-way ball valve was used to control which gas mixture entered the labelling chamber. This valve was connected to the labelling chamber by a short tube (15 cm long, Ø 0.125 inch), minimizing dead-volume between the valve and chamber. **(C)** Custom-made labelling chamber. The internal faces of the two Plexiglas plates were sealed at the edges by soft rubber gaskets. The internal dimensions of the closed chamber were 7.1 cm x 9.8 cm x 1.2 cm (volume 83.5 mL). At the flow rate of 10 L min^{-1}, used during our experiment, gas half-time was 0.35 sec (calculated as described in Szecowka *et al.*, 2013). The upper plate (lid) had a liquid N_2 port connected to a plastic funnel via a short PVC tube, which during labelling was closed using a removable transparent plastic plug. The bottom part of the chamber had an exit port to release liquid N_2, also fitted with a removable plastic plug. Two openings in the side of the chamber walls were fitted with metal connectors for attachment of PVC tubing to serve as gas inlet and outlet ports. **(D)** Labelling set- up showing the 4-way ball valve and labelling chamber containing two maize leaves. **(E and F)** quenching of the leaves in the chamber with liquid N_2. removing The plugs from the entry and exit ports were removed and approx. 300 mL of liquid N_2 were immediately poured into the entry port, with the liquid nitrogen flowing out through the exit port into a large plastic beaker. **(G)** Removal of non-illuminated parts of the leaves that were shaded by the rubber gasket while remaining tissue was kept frozen by flooding with liquid N_2.

A

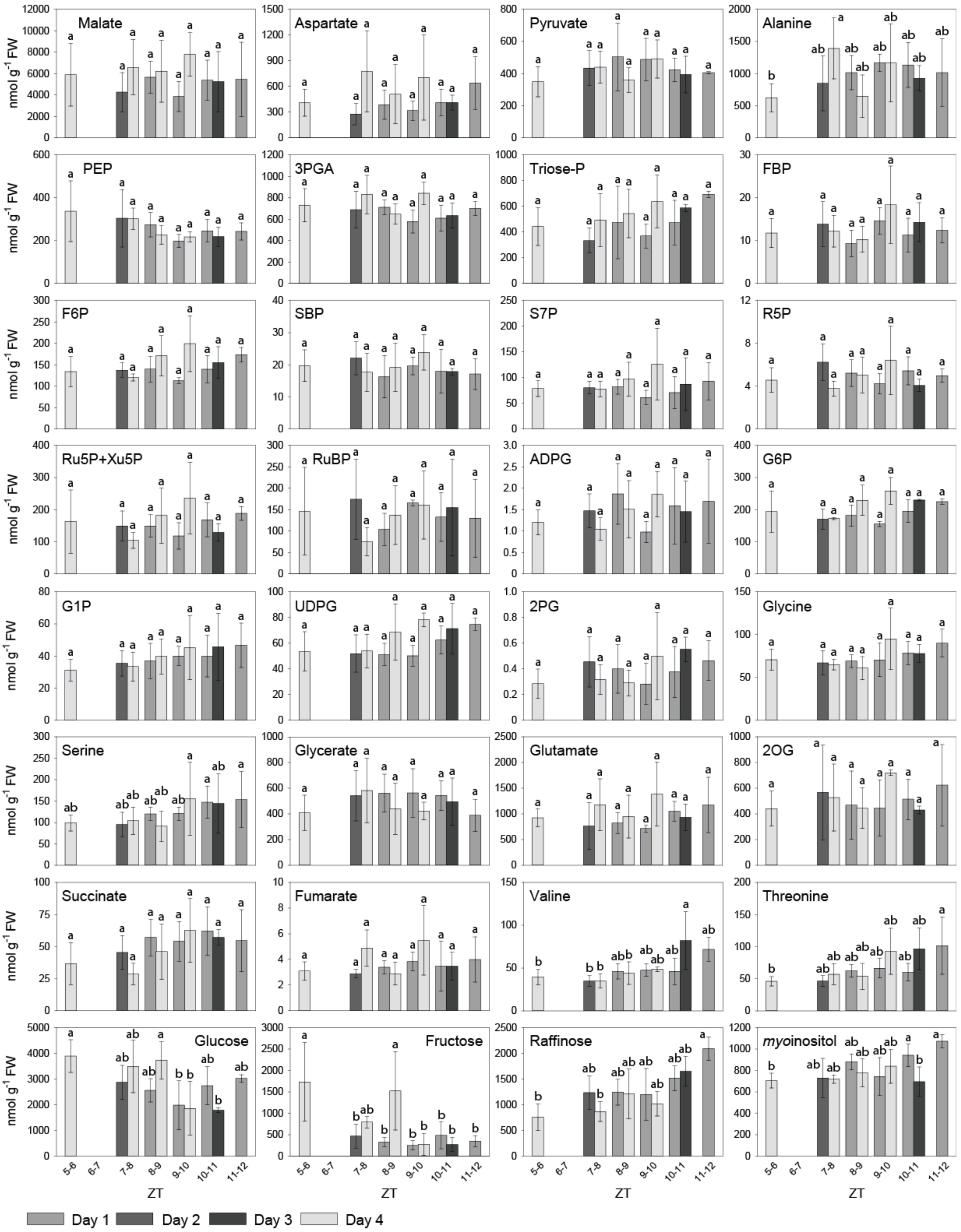

Supplemental Figure S2. Comparison of metabolite pool sizes at different days and times during the harvesting period.

Continued on the next page. See also next page for legend.

B

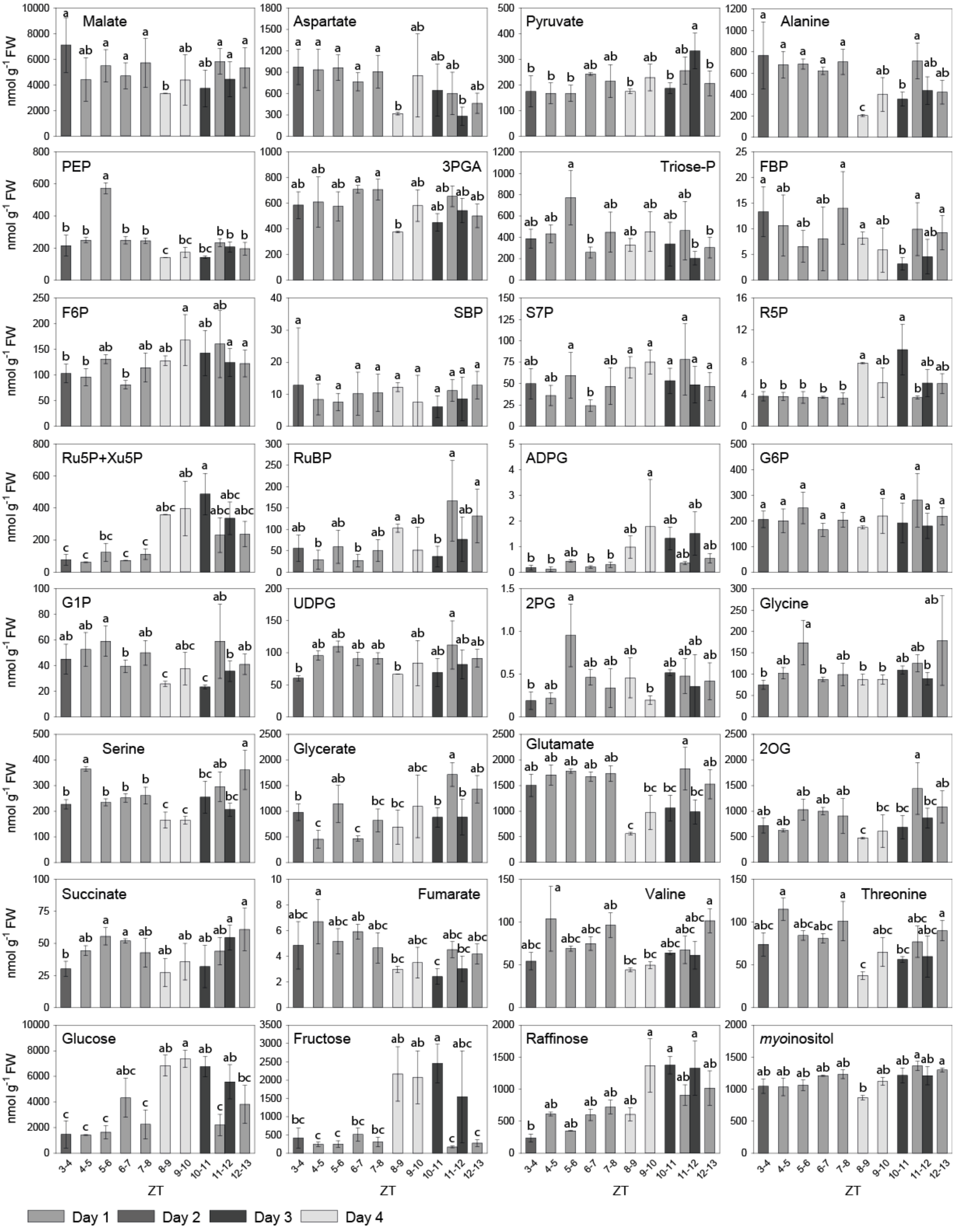

Supplemental Figure S2. Comparison of metabolite pool sizes at different days and times during the harvesting period. (A) Metabolic contents in medium light (ML), and **(B)** in low light (LL). For a given compound, amounts of all isotopomers were summed. Data were not corrected for the presence of inactive pools. Total amounts of pyruvate, PEP and 3PGA were determined enzymatically. Amounts

are expressed as nmol g^{-1} FW. Both labelling experiments were performed over the course of 4 days. For each day of labelling, samples were grouped by 1 hour harvesting period. Days are indicated by a colour code. The x-axis corresponds to ZT expressed as 1 hour periods. Mean ± SD, n = 2 to 19 and 2 to 19 replicates in ML and LL, respectively. Statistical analysis was performed using one-way ANOVA and different letters indicate significantly different values according to Tukey's *post-hoc* test (α > 0.05). The amounts in each sample and the day and time of labelling are provided in Supplemental Dataset S5. Abbreviations listed in alphabetical order: 2PG, 2-phosphoglycolate; 2OG, 2-oxoglutarate, 3PGA, 3-phosphoglycerate; ADPG, ADP-glucose; F6P, fructose 6-phosphate; FBP, fructose 1,6-bisphosphate; G1P, glucose 1-phosphate; G6P, glucose 6-phosphate; PEP, phospho*enol*pyruvate; R5P, ribose 5-phosphate; Ru5P+Xu5P , ribulose 5-phosphate + xylulose 5-phosphate; RuBP, ribulose 1,5-bisphosphate; S7P, sedoheptulose 7-phosphate; SBP, sedoheptulose 1,7-bisphosphate; Triose-P, triose-Phosphate (dihydroxyacetone phosphate); UDPG, UDP-glucose.

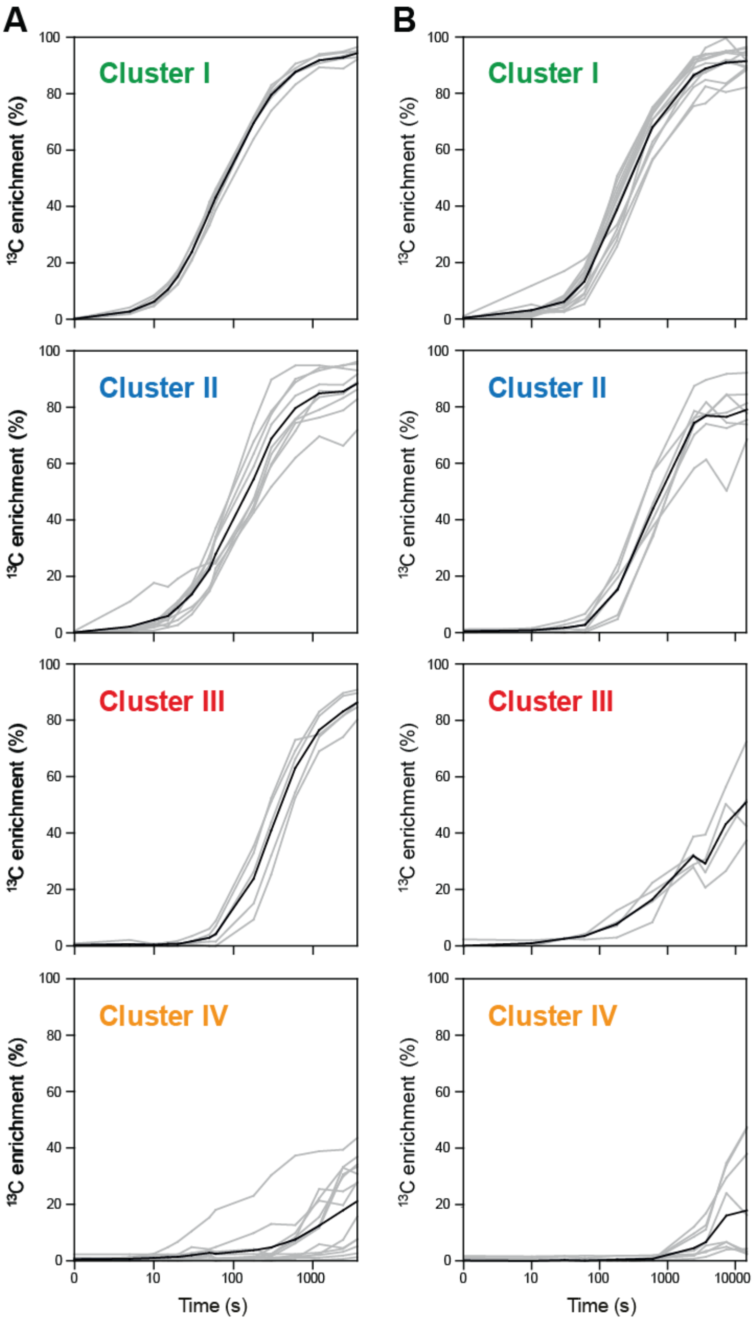

C

	ML	LL
**CBC, starch and sucrose synthesis intermediates**		
3PGA	I	I
Triose-P	I	I
FBP	I	I
F6P	II	I
SBP	IV	III
S7P	I	I
R5P	II	I
Ru5P+Xu5P	II	I
RuBP	I	I
ADPG	II	I
G6P	II	I
G1P	II	II
UDPG	II	II
**C carriers in CO_2 shuttle**		
PEP	III	II
Malate	IV	III
Aspartate	II	I
Pyruvate	III	II
Alanine	III	II
**Photorespiratory intermediates**		
2PG	II	I
Glycine	III	II
Serine	III	II
Glycerate	IV	III
**Other organic acids, amino acids and sugars**		
Glutamate	IV	IV
2OG	IV	IV
Succinate	IV	IV
Fumarate	IV	III
Valine	IV	IV
Threonine	IV	IV
Glucose	IV	IV
Fructose	IV	IV
Raffinose	IV	IV
*myo*inositol	IV	IV

Supplemental Figure S3. Overview of measured ^{13}C enrichment kinetics by *k*-means. (A) Clustering in medium light (ML), and **(B)** in low light (LL) using raw data (not corrected for inactive pools). In each clusters, grey lines show the ^{13}C enrichment of individual metabolites and black lines show average ^{13}C enrichment of all metabolites in the cluster. The x-axis corresponds to the labelling time on a log scale. The x-axis corresponds to the labelling time on a log scale. The duration of the applied pulses were extended to match the expected slower flow of ^{13}C through metabolism in LL. Therefore the scale of the x-axes in is adjusted such that the x-axis is 4-fold more expanded in LL **(B)** than in ML **(A)** plots. Data are presented in Supplemental Dataset S6. **(C)** Metabolites grouped according to the process in which they are primarily involved and summary of their cluster grouping in ML and LL. For abbreviations, see legend of Supplemental Figure S2 or Supplemental Table S1.

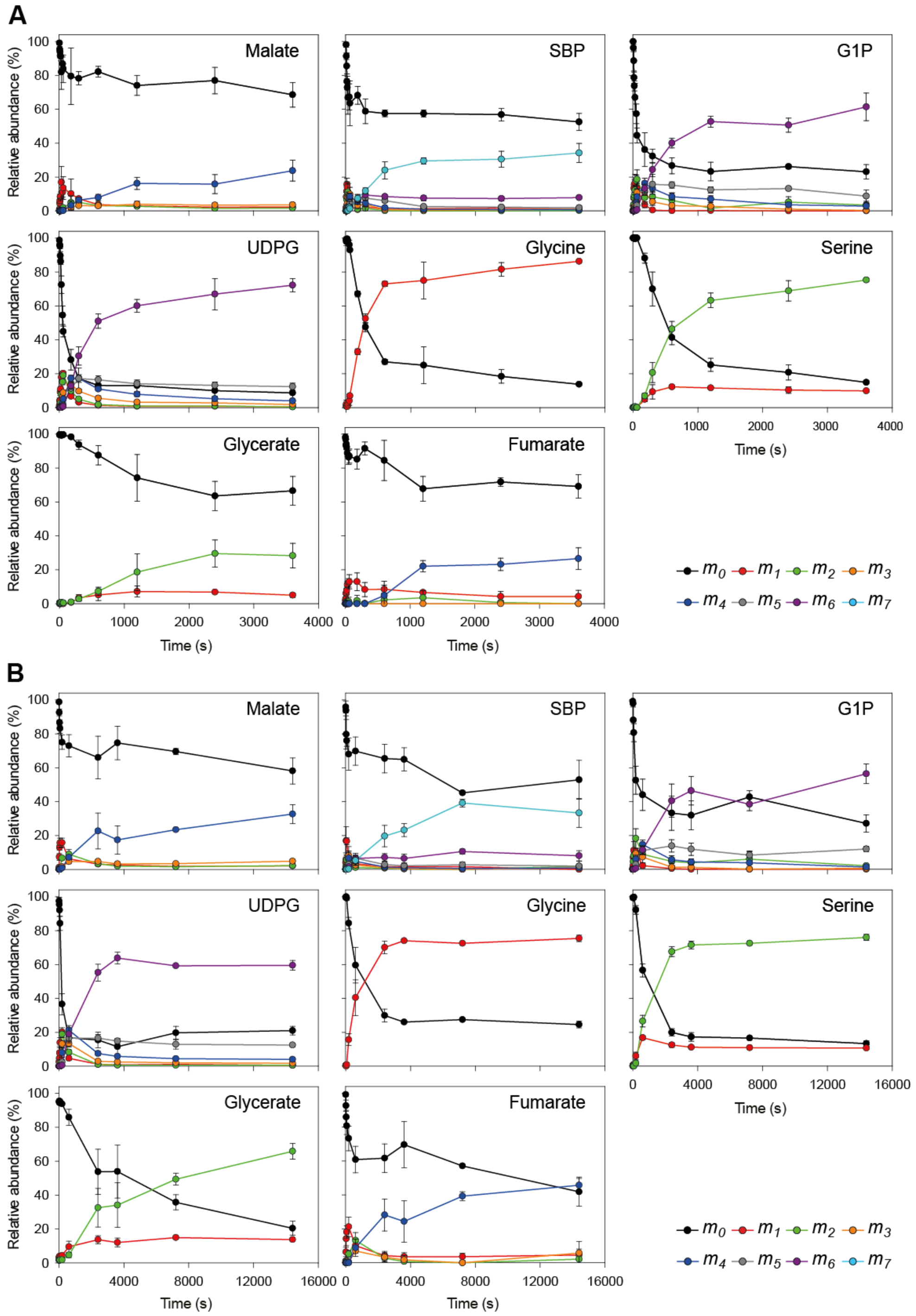

Supplemental Figure S4. Detailed labelling kinetics of metabolites presenting two or more pools, of which only one is labelled by newly fixed ^{13}C. (A) Isotopomer abundance kinetics in medium light (ML), and **(B)** in low light (LL). The relative abundance of each isotopomer (m_n) for a given metabolite is represented; n is the number of ^{13}C atoms incorporated. For all metabolites presented the m_o isotopomer (shown in black) falls initially but then plateaus, whilst the labelled isotopomers, including the heavily labelled isotopomers rise but also approach a plateau with the heavily labelled isotopomers dominating. Glycerate in LL did not exhibit an inactive pool but is still presented. The x-axis corresponds to the labelling time on a log scale. Mean ± SD, n = 3 to 6 and 2 to 6 replicates in ML and LL, respectively. Data are presented in Supplemental Datasets S2 and S4. For abbreviations, see legend of Supplemental Figure S2 or Supplemental Table S1.

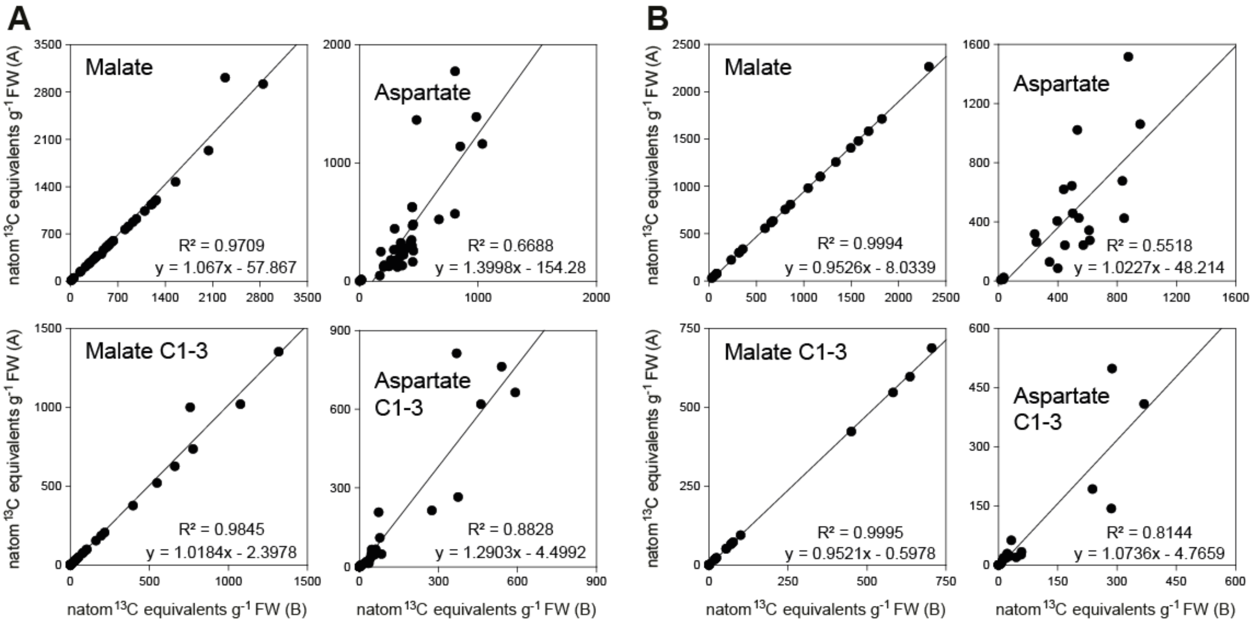

Supplemental Figure S5. Regression plots of ^{13}C amounts and positional ^{13}C amounts in malate and aspartate calculated with two approaches. (A) Regression plots in medium light (ML), and **(B)** in low light (LL). ^{13}C amounts (natom ^{13}C equivalents g^{-1} FW) in metabolites were estimated by multiplying the number of ^{13}C atoms per isotopomer by the corresponding isotopomer amount, and then summing the results. ^{13}C amounts in the C1-3 positions of malate and aspartate were similarly estimated, considering these compounds as three-carbon compounds and modifying the number of ^{13}C atoms per isotopomer accordingly (calculation steps are presented in Supplemental Datasets S7-8). These estimations were done using two approaches, either using isotopomer amounts obtained by multiplying isotopomer abundance by the average metabolite pool size (approach A), or using isotopomer amounts determined at each time point (approach B). ^{13}C amounts for malate, aspartate, C1-3 positions of malate and aspartate until time point 180 sec were used to compare both approaches. Results obtained with approach A (y-axis) were plotted against results obtained with approach B (x-axis). Slope and R^2 for each regression line are indicated.

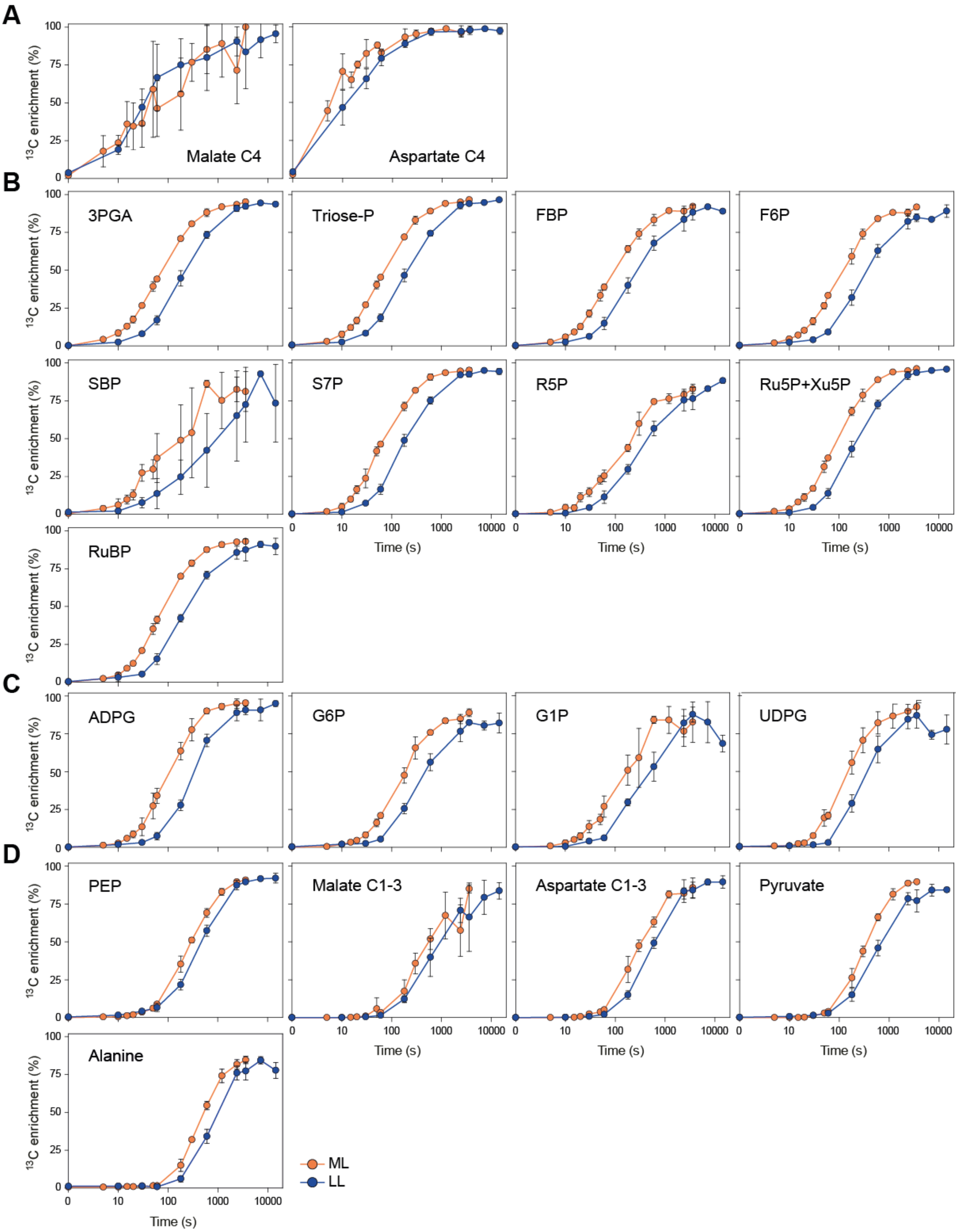

Supplemental Figure S6. ^{13}C enrichments (%) of individual metabolites in medium and low light.

Continued on the next page. See also next page for legend.

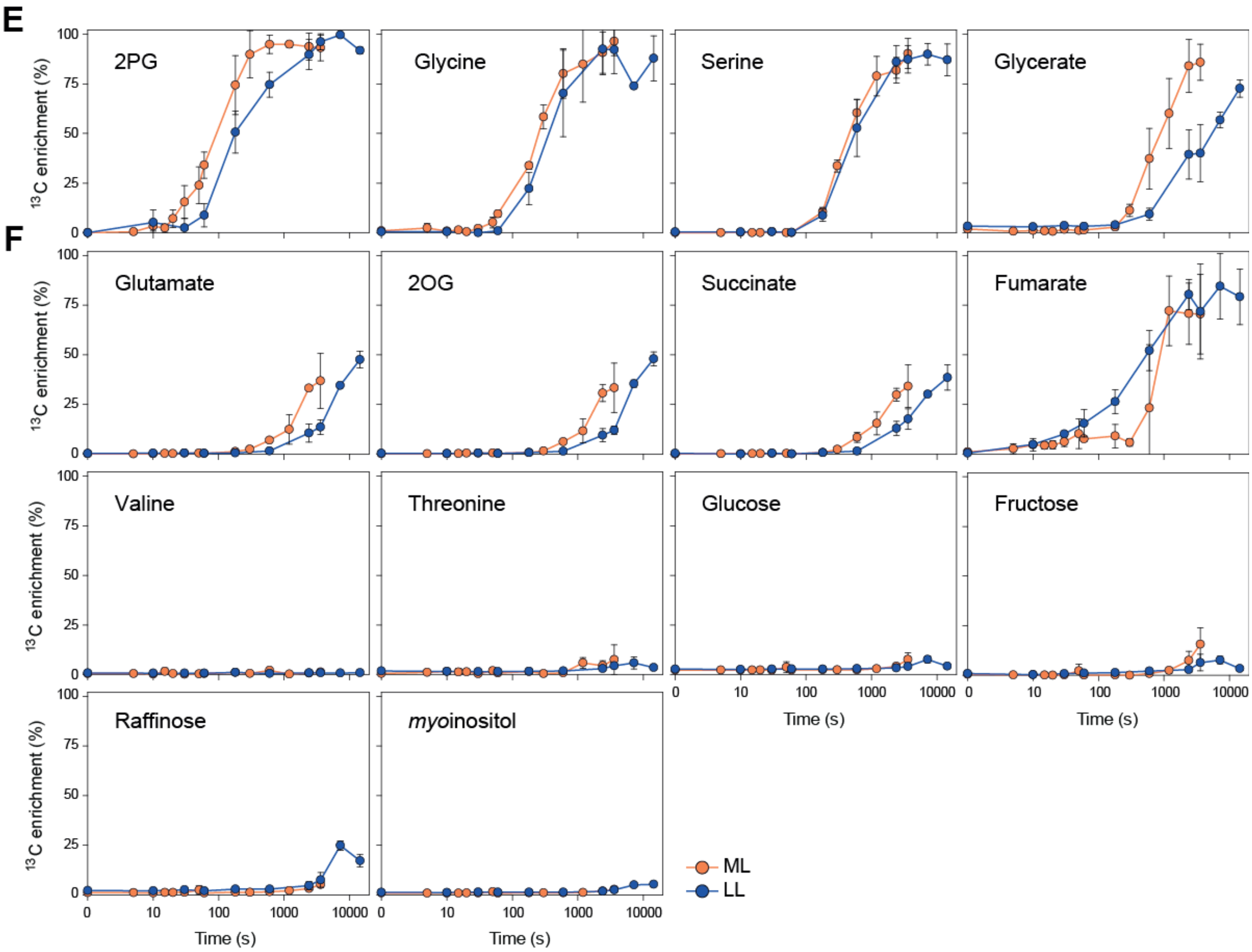

Supplemental Figure S6. ^{13}C enrichments (%) of individual metabolites in medium and low light. Carbon position-dependent ^{13}C enrichments were separately calculated for the C4 and C1-3 positions of malate and aspartate (for further information about calculations see Supplemental Dataset S9). **(A)** C4 positions of malate and aspartate, **(B)** 3PGA, Triose-P and other CBC intermediates, **(C)** starch and sugar synthesis intermediates, **(D)** PEP, C1-3 positions of malate and aspartate, pyruvate and alanine, **(E)** photorespiratory intermediates, and **(F)** other organic acids, amino acids and sugars. Enrichments are shown in orange for medium light (ML) and in blue for low light (LL). Mean ± SD, n = 3 to 6 and 2 to 6 replicates in ML and LL, respectively. Data are presented in Supplemental Table S3. For abbreviations, see legend of Supplemental Figure S2 or Supplemental Table S1.

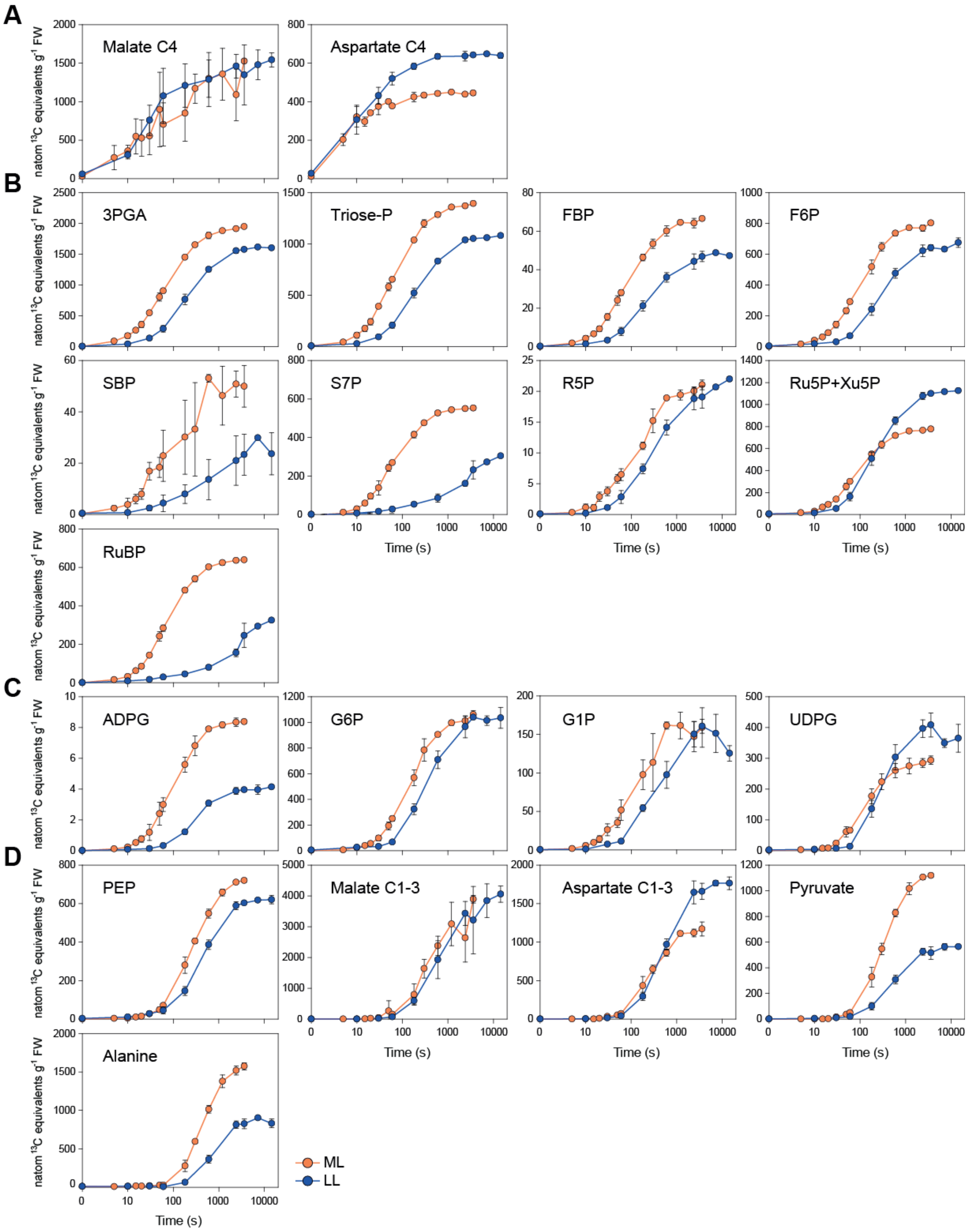

Supplemental Figure S7. **^{13}C amounts (natom ^{13}C equivalents g^{-1} FW) of individual metabolites in medium and low light.**

Continued on the next page. See also next page for legend.

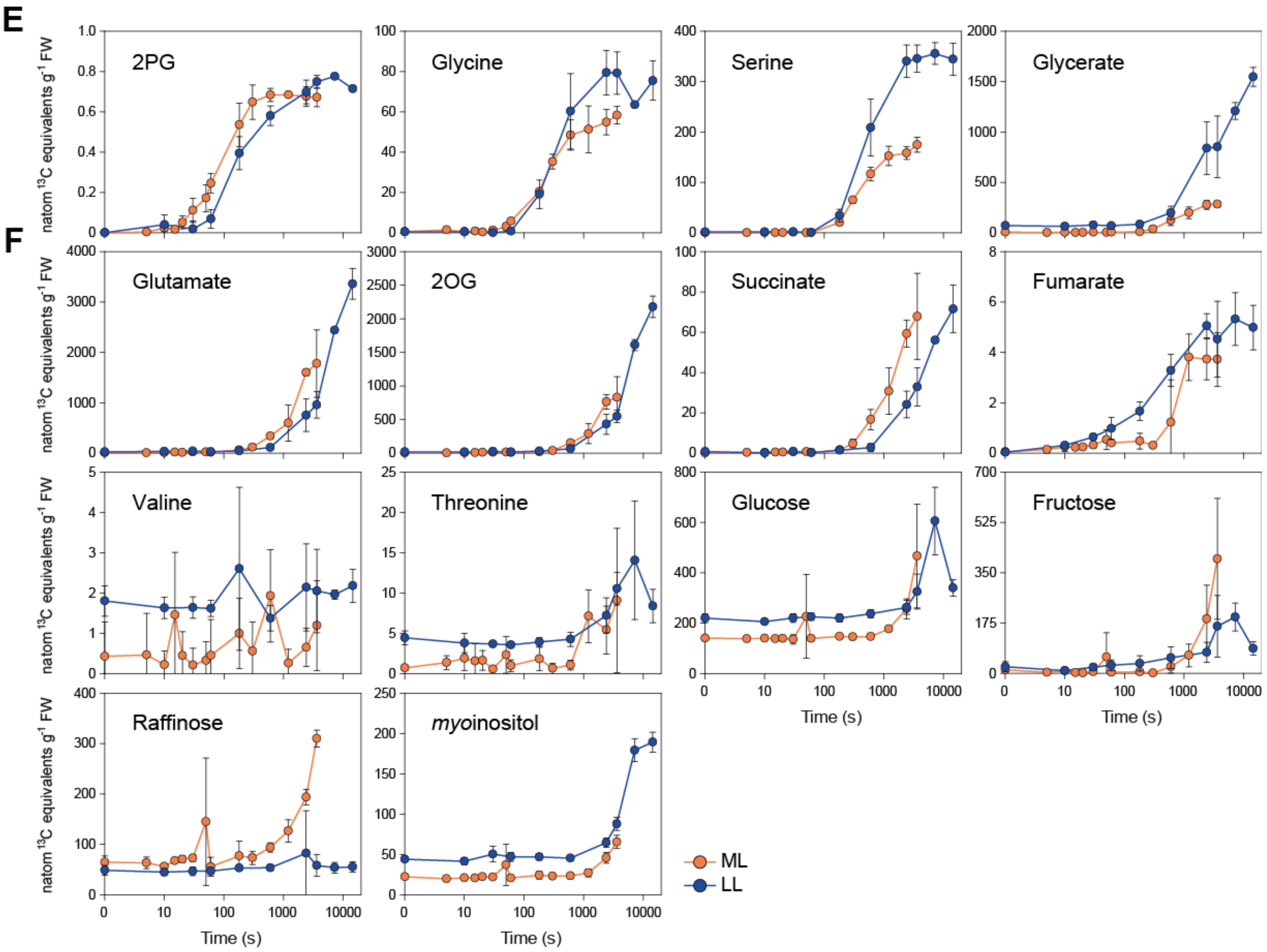

Supplemental Figure S7. ^{13}C amounts (natom ^{13}C equivalents g^{-1} FW) of individual metabolites in medium and low light. Carbon position-dependent ^{13}C amounts were separately calculated for the C4 and C1-3 positions of malate and aspartate (for further information about calculations see Supplemental Datasets S7-8). **(A)** C4 positions of malate and aspartate, **(B)** 3PGA, Triose-P and other CBC intermediates, **(C)** starch and sugar synthesis intermediates, **(D)** PEP, C1-3 positions of malate and aspartate, pyruvate and alanine, **(E)** photorespiratory intermediates, and **(F)** other organic acids, amino acids and sugars. ^{13}C amounts (natom ^{13}C equivalents g^{-1} FW) are shown in orange for medium light (ML) and in blue for low light (LL). Mean ± SD, n = 3 to 6 and 2 to 6 replicates in ML and LL, respectively. Data are presented in Supplemental Table S4. For abbreviations, see legend of Supplemental Figure S2 or Supplemental Table S1.

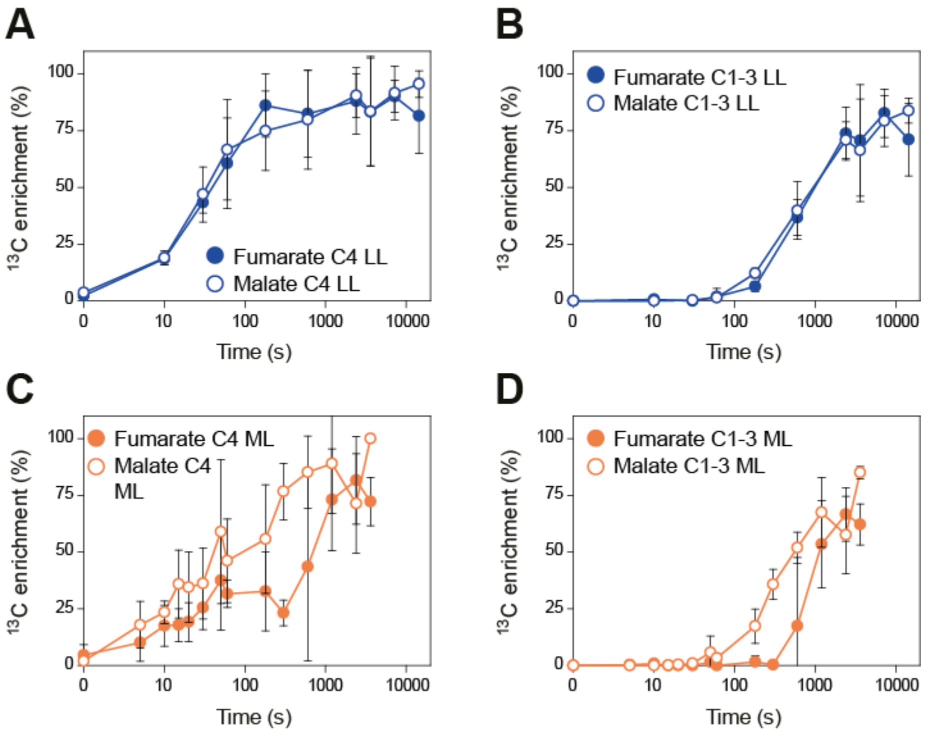

Supplemental Figure S8. Comparison of the positional labelling kinetics of fumarate and malate. Carbon position-dependent ^{13}C enrichments were separately calculated for the C4 and C1-3 positions of the active pools of fumarate and malate (for further information about calculations see Supplemental Dataset S9). ^{13}C enrichment (%) kinetics of **(A)** C4 positions of fumarate and malate in low light (LL), **(B)** C1-3 positions of fumarate and malate in LL, **(C)** C4 positions of fumarate and malate in medium light (ML), and **(D)** C1-3 positions of fumarate and malate in ML. Enrichments are shown in orange for ML and in blue for LL. Fumarate is shown as filled circle, malate as open circle. Mean ± SD, n = 3 to 6 and 2 to 6 replicates in ML and LL, respectively. Data are presented in Supplemental Table S3.

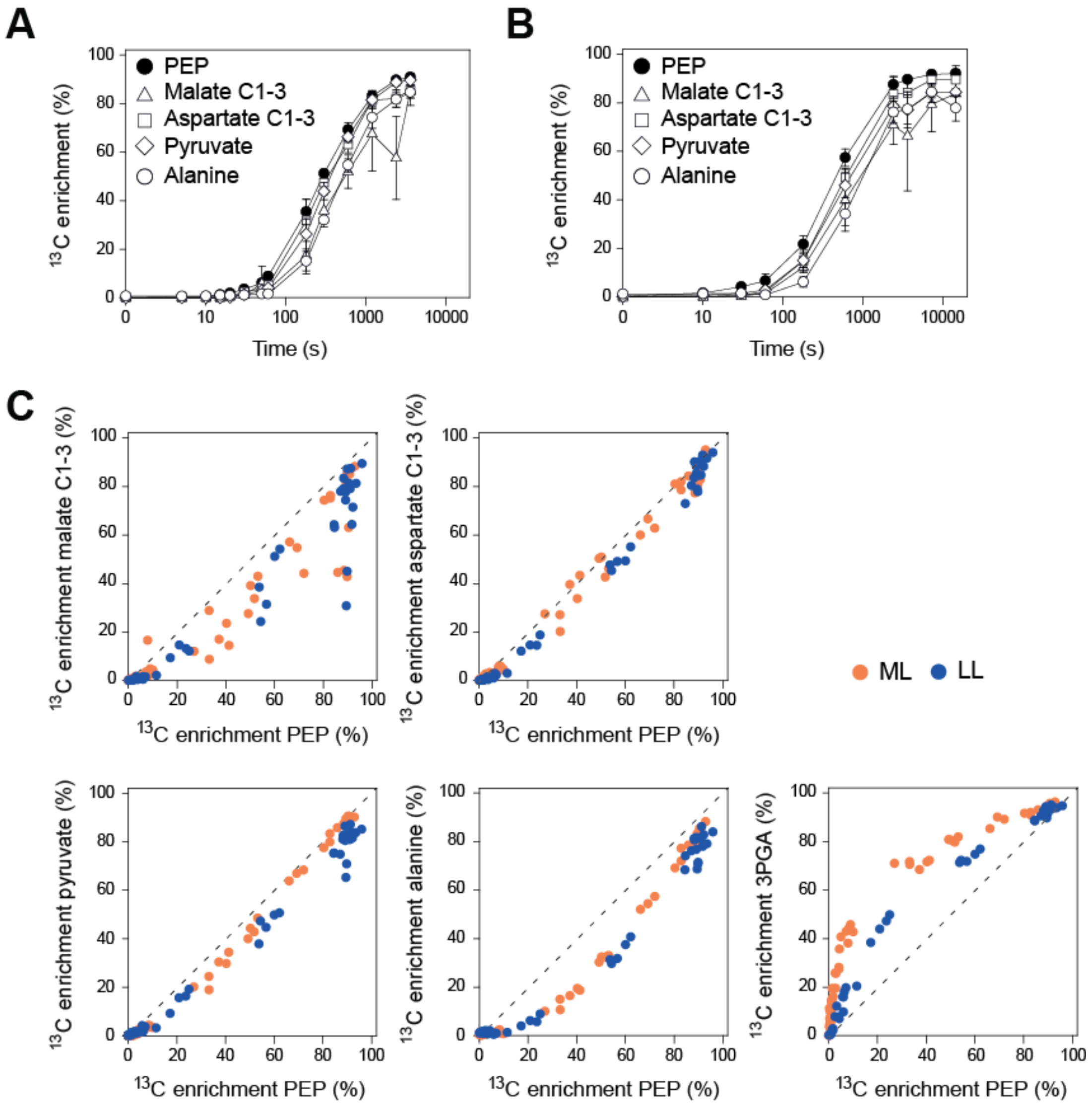

Supplemental Figure S9. Labelling kinetics of PEP, C1-3 positions of malate and aspartate, pyruvate and alanine. **(A)** ^{13}C enrichment (%) kinetics in medium light (ML), and **(B)** in low light (LL). PEP is shown as filled circle, C4 position of malate as open triangle, C4 position of aspartate as open square, pyruvate as open diamond, and alanine as open circle. Mean ± SD, n = 3 to 6 and 2 to 6 replicates in ML and LL, respectively. **(C)** Regression plot of ^{13}C enrichment of PEP (x-axis) versus ^{13}C enrichment of metabolite X (y-axis) in ML (orange) and LL (blue). All individual samples are shown. The dotted line indicates a regression with a slope of 1. In **(A)** and **(B)**, x-axes correspond to the labelling time on a log scale. Data are presented in Supplemental Table S3 and Supplemental Dataset S6. For abbreviations, see legend of Supplemental Figure S2 or Supplemental Table S1.

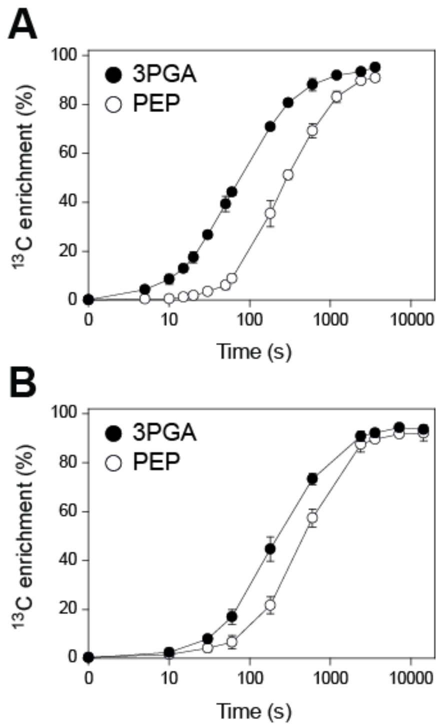

Supplemental Figure S10. Labelling kinetics of 3PGA and PEP. (A) ^{13}C enrichment (%) of 3PGA and PEP in medium light (ML), and **(B)** in low light (LL). 3PGA is shown as filled circle and PEP as open circle. Mean ± SD, n = 3 to 6 and 2 to 6 replicates in ML and LL, respectively. All x-axes correspond to the labelling time on a log scale. Data are presented in Supplemental Table S3. For abbreviations, see legend of Supplemental Figure S2 or Supplemental Table S1.

Supplemental Table S1. Information about analyzed metabolites. Metabolite abbreviations, the metabolic sector they are assigned to, the method of analysis, and whether the labelling pattern indicated the presence of a pool that is not labelled by newly fixed ^{13}C ('inactive' pools). *Metabolites for which total amounts were determined enzymatically.

Metabolic sector	Metabolite	Abbreviation	Method of analysis	Presence of an "inactive" pool
CO_2 shuttle	Phospho*enol*pyruvate	PEP	LC-MS/MS*	yes
	Malate		LC-MS/MS	
	Aspartate		LC-MS/MS	
	Pyruvate		GC-MS*	
	Alanine		GC-MS	
CBC	3-phosphoglycerate	3PGA	LC-MS/MS*	yes
	Dihydroxyacetone phosphate	DHAP (Triose-P)	LC-MS/MS	
	Fructose 1,6-bisphosphate	FBP	LC-MS/MS	
	Fructose 6-phosphate	F6P	LC-MS/MS	
	Sedoheptulose 1,7-bisphosphate	SBP	LC-MS/MS	
	Sedoheptulose 7-phosphate	S7P	LC-MS/MS	
	Ribose 5-phosphate	R5P	LC-MS/MS	
	Ribulose 5-phosphate + xylulose 5-phosphate	Ru5P+Xu5P	LC-MS/MS	
	Ribulose 1,5-bisphosphate	RuBP	LC-MS/MS	
Starch and sucrose synthesis	ADP-glucose	ADPG	LC-MS/MS	
	Glucose 6-phosphate	G6P	LC-MS/MS	
	Glucose 1-phosphate	G1P	LC-MS/MS	yes
	UDP-glucose	UDPG	LC-MS/MS	yes
Photorespiration	2-phosphoglycolate	2PG	LC-MS/MS	
	Glycine		GC-MS	yes
	Serine		GC-MS	yes
	Glycerate		GC-MS	yes (at medium light)
Other organic acids, amino acids and sugars	Glutamate		LC-MS/MS	yes
	2-oxoglutarate	2OG	LC-MS/MS	
	Succinate		LC-MS/MS	
	Fumarate		GC-MS	
	Valine		GC-MS	
	Threonine		GC-MS	
	Glucose		GC-MS	
	Fructose		GC-MS	
	Raffinose		GC-MS	
	*myo*inositol		GC-MS	

Supplemental Table S2. Metabolite active pool sizes in in medium and low light. For a given compound, amounts of all isotopomers were summed. Total amounts of pyruvate, PEP and 3PGA were determined enzymatically. Amounts are expressed as nmol g^{-1} FW. Mean ± SD, n = 50 to 60 and 43 to 45 replicates in medium light (ML) and in low light (LL), respectively. The amounts of the unlabelled form and each ^{13}C-isotopomer are provided in Supplemental Datasets S2 and S4, and the total amounts in Supplemental Dataset S5. Data are presented as plots in Fig. 2. Malate, SBP, G1P, UDPG, serine, glycine, fumarate and (in ML only) glycerate were corrected for the presence of an inactive pool. For abbreviations, see legend of Supplemental Figure S2 or Supplemental Table S1.

Metabolite	mean ± SD	
	ML	LL
Malate	1527 ± 260	1615 ± 281
Aspartate	455 ± 269	657 ± 345
Pyruvate	416 ± 114	223 ± 68.9
Alanine	930 ± 403	535 ± 210
PEP	264 ± 89	225 ± 89.8
3PGA	683 ± 138	571 ± 123
Triose-P	481 ± 184	374 ± 182
FBP	12 ± 4.27	8.85 ± 5.26
F6P	146 ± 38.6	126 ± 37.5
SBP	8.8 ± 1.5	4.6 ± 1.47
S7P	83.1 ± 29.7	53.1 ± 23.1
R5P	5.08 ± 1.51	4.98 ± 2.04
Ru5P+Xu5P	161 ± 70.1	235 ± 150
RuBP	138 ± 71.5	82.7 ± 63.9
ADPG	1.46 ± 0.64	0.73 ± 0.81
G6P	199 ± 46.6	210 ± 54.7
G1P	32 ± 5.74	30.5 ± 6.92
UDPG	60.1 ± 16.1	87 ± 22.7
UDPG	53 ± 9.17	78.3 ± 17
2PG	0.36 ± 0.17	0.39 ± 0.26
Glycine	60.5 ± 7.57	86 ± 13.9
Serine	96.8 ± 20.4	198 ± 28.7
Glycerate	165 ± 21	1065 ± 444
Valine	45.1 ± 14.9	76.1 ± 24.4
Threonine	60 ± 21.6	78.5 ± 23.6
Glutamate	963 ± 346	1412 ± 426
2OG	498 ± 205	909 ± 357
Succinate	49.6 ± 19.5	46.4 ± 15.8
Fumarate	1.32 ± 0.19	1.58 ± 0.23
Glucose	3039 ± 958	3962 ± 2271
Fructose	848 ± 806	852 ± 958
Raffinose	1213 ± 436	912 ± 430
*myo*inositol	808 ± 149	1196 ± 142

Supplemental Table S3. ^{13}C enrichment kinetics for individual metabolites in medium and low light. **(A)** ^{13}C enrichment kinetics in medium light (ML), and **(B)** in low light (LL). Carbon position-dependent ^{13}C enrichments were separately calculated for the C4 and C1-3 positions of malate and aspartate (for further information about calculations see Supplemental Dataset S9). Data used for malate, SBP, G1P, UDPG, serine, glycine, fumarate and (in ML only) glycerate were corrected for the presence of an inactive pool. Mean ± SD, n = 3 to 6 and 2 to 6 replicates in ML and LL, respectively. Data are presented as plots in Supplemental Fig. S6. For abbreviations, see legend of Supplemental Figure S2 or Supplemental Table S1.

**A**	Kinetic (s)													
Metabolite	0	5	10	15	20	30	50	60	180	300	600	1200	2400	3600
Malate C4	2 ± 1.3	17.9 ± 10.3	23.5 ± 5	35.9 ± 14.9	34.5 ± 15.4	36.2 ± 15.7	58.9 ± 31.7	46.1 ± 18.4	55.7 ± 23.8	76.7 ± 12.4	85.2 ± 15.9	89 ± 22	71.4 ± 21.9	100 ± 0
Aspartate C4	2.6 ± 1.6	44.5 ± 6.6	70.5 ± 11.7	65 ± 5.2	75 ± 2.2	82.4 ± 9.3	87.9 ± 1.9	82.9 ± 2.5	93.2 ± 5.5	95.2 ± 2.7	97.1 ± 1.9	98.8 ± 0.3	96.2 ± 2.3	97.8 ± 2.9
3PGA	0.1 ± 0.1	4.3 ± 1	8.5 ± 2	12.9 ± 1.3	17.4 ± 2.3	26.6 ± 1.2	39.3 ± 3.2	44.1 ± 1.2	70.8 ± 1.4	80.6 ± 0.9	88 ± 2.6	91.8 ± 0.9	93.4 ± 0.9	95.1 ± 0.9
Triose-P	0.4 ± 0.4	3 ± 1.1	7.7 ± 2	12.2 ± 1.9	16.7 ± 2.1	27.1 ± 1.2	40.3 ± 2.6	45.3 ± 0.8	71.9 ± 1.1	83.1 ± 2.6	89 ± 1.3	94 ± 1.1	95 ± 0.2	96.5 ± 0.8
FBP	0 ± 0.1	2.5 ± 0.7	5.8 ± 1.8	9.1 ± 1.2	12.7 ± 2	21.4 ± 2.4	33.3 ± 3.5	38.8 ± 2	64.1 ± 2.3	74.1 ± 3.3	83.2 ± 3.6	89.3 ± 1	88.9 ± 3.4	92.2 ± 1.1
F6P	0.1 ± 0.1	1.9 ± 0.6	4.6 ± 1.1	7.1 ± 0.8	10.3 ± 1.5	16.4 ± 2.5	26.5 ± 2.1	33.3 ± 1.5	59.1 ± 5.1	73.9 ± 3.2	84.1 ± 1.8	88.1 ± 1.1	88 ± 2.3	91.7 ± 1.5
SBP	0.5 ± 0.4	3.9 ± 1.7	6.2 ± 4.1	9.8 ± 2.9	12.9 ± 3.3	27.4 ± 5.5	29.8 ± 6.3	37.1 ± 16.2	48.9 ± 23.4	53.9 ± 29.6	86.2 ± 2.4	75.2 ± 18.6	82.5 ± 8.2	81.1 ± 13.2
S7P	0.1 ± 0.1	1.9 ± 0.7	4.9 ± 2.4	10 ± 1.7	16.4 ± 2.6	23.8 ± 6.2	41.7 ± 3	46.3 ± 1.4	71.3 ± 2.8	81.9 ± 1.2	90.7 ± 2.1	93.5 ± 1	94.6 ± 0.6	95.1 ± 1.9
R5P	0.3 ± 0.2	1.3 ± 0.5	4.4 ± 2.2	4.3 ± 2	11.3 ± 2.9	14.7 ± 2.9	22.8 ± 2.8	25.5 ± 3.8	43.9 ± 2.4	59.9 ± 7.6	74.4 ± 0.5	76.4 ± 3.2	78.9 ± 2.5	82.9 ± 3.1
Ru5P+Xu5P	0.2 ± 0.2	1.9 ± 0.3	3.5 ± 0.8	8.1 ± 1.3	11.2 ± 2.5	17.1 ± 1.6	31.4 ± 3.8	37.2 ± 1	68 ± 2.6	78.6 ± 4	88.9 ± 0.3	94 ± 0.9	94.7 ± 0.8	96.2 ± 0.6
RuBP	0.2 ± 0.2	2.4 ± 0.7	4.7 ± 1.4	9.1 ± 0.9	12.4 ± 1.3	20.8 ± 1.3	35.2 ± 3.6	41.3 ± 2.4	69.9 ± 1.6	78.7 ± 2.1	87.6 ± 1.7	90.8 ± 1.7	92.5 ± 1.6	92.9 ± 0.9
ADPG	0.1 ± 0.1	1.4 ± 0.6	2.7 ± 1.2	5.9 ± 0.8	8.6 ± 2.3	13.6 ± 5.9	27.4 ± 8.4	34.1 ± 5	63.6 ± 5.6	77.6 ± 7.3	90 ± 1.7	93 ± 2	95 ± 3.1	95.4 ± 1.4
G6P	0.2 ± 0.1	0.5 ± 0.4	2 ± 0.4	3.3 ± 0.5	4.6 ± 0.6	8.2 ± 1.8	16.2 ± 2.6	21 ± 1.7	47.6 ± 5.1	65.7 ± 7.3	75.8 ± 1.4	83.6 ± 1.6	84.8 ± 3.1	89 ± 2.6
G1P	0 ± 0	0.9 ± 0.5	2.8 ± 1.9	5 ± 1.2	7.3 ± 2.4	13.6 ± 4	18.4 ± 3.4	26.9 ± 7	50.8 ± 10	59 ± 19.5	84.1 ± 2.4	84 ± 8.9	76.6 ± 10.1	82.7 ± 13.3
UDPG	0.3 ± 0.2	0.9 ± 0.4	0.9 ± 0.3	2.3 ± 0.7	2.8 ± 0.4	7.6 ± 1.2	19.3 ± 5.4	20.8 ± 2.2	55.8 ± 7.6	70.6 ± 8.2	82.1 ± 7.3	86.6 ± 8	89.7 ± 4.7	92.6 ± 4.3
PEP	0.1 ± 0.1	0.5 ± 0.2	0.5 ± 0.2	1.2 ± 0.2	1.8 ± 0.7	3.5 ± 0.8	6.1 ± 1.7	8.8 ± 0.8	35.3 ± 5.3	51.1 ± 1.7	69.2 ± 2.9	83 ± 2.3	89.6 ± 0.8	90.8 ± 1.5
Malate C1-3	0 ± 0	0.1 ± 0.1	0 ± 0	0.1 ± 0	0.4 ± 0.3	1 ± 0.5	5.8 ± 7.3	3.4 ± 1.3	17.4 ± 7.5	35.8 ± 6.7	51.9 ± 6.9	67.5 ± 15.4	57.5 ± 17	85 ± 2.7
Aspartate C1-3	0 ± 0	0 ± 0	0.2 ± 0.2	0.1 ± 0.1	0.6 ± 0.6	2.4 ± 0.3	3.8 ± 1.1	5.1 ± 0.9	31.8 ± 8.7	47.4 ± 3.8	63.1 ± 3.3	81.4 ± 2.4	82 ± 3.7	85.8 ± 6.6
Pyruvate	0 ± 0	0 ± 0	0 ± 0	0 ± 0	0.2 ± 0.2	1.3 ± 0.5	2.9 ± 0.4	3.9 ± 0.4	26.3 ± 6.2	43.8 ± 3.6	66.3 ± 2.3	81.5 ± 3.6	88.7 ± 1.4	89.7 ± 0.7
Alanine	0.7 ± 0.2	0.6 ± 0.4	0.7 ± 0.1	0.9 ± 0.1	0.9 ± 0.3	1.1 ± 0.5	1.5 ± 0.3	1.5 ± 0.7	15 ± 4	32 ± 1.2	54.6 ± 2.7	74.2 ± 4.4	81.7 ± 3.2	84.7 ± 2.5
Malate	0.5 ± 0.3	4.5 ± 2.6	5.9 ± 1.3	9 ± 3.7	8.9 ± 4	9.8 ± 4.3	19 ± 13.1	14 ± 5.6	27 ± 11.4	46 ± 8.1	60.2 ± 9.1	72.8 ± 17	61 ± 18.2	88.8 ± 2
Aspartate	0.6 ± 0.4	11.1 ± 1.7	17.8 ± 3	16.3 ± 1.3	19.2 ± 0.9	22.4 ± 2.5	24.8 ± 1.2	24.6 ± 0.4	47.2 ± 7.3	59.4 ± 3.5	71.6 ± 2.2	85.7 ± 1.8	85.6 ± 3.1	88.8 ± 5.3
2PG	0 ± 0	0.5 ± 1	3.3 ± 2.5	2.4 ± 2.1	7.1 ± 4.5	15.5 ± 8.3	23.9 ± 9.2	34.1 ± 6.7	74.3 ± 14.9	89.8 ± 11.9	94.8 ± 4.5	94.9 ± 1.5	93.7 ± 6.9	93.1 ± 6.5
Glycine	1 ± 1	2.4 ± 2.4	0.8 ± 1	1.4 ± 1.1	0.5 ± 1	2.1 ± 1.3	5.2 ± 2.8	9.5 ± 1.7	33.8 ± 1.9	58.3 ± 6	80.2 ± 12.4	84.9 ± 19.1	90.7 ± 10.4	96.4 ± 7.2
Serine	0 ± 0	0 ± 0	0.2 ± 0.5	0 ± 0	0 ± 0	0.6 ± 0.7	0 ± 0	0 ± 0	10.6 ± 2.2	33.7 ± 3	60.4 ± 6.9	78.9 ± 9.9	81.7 ± 6.5	90.2 ± 7.6
Glycerate	1.9 ± 0.6	0.8 ± 0.3	1.1 ± 0.6	0.9 ± 1	0.7 ± 0.8	1.7 ± 0.7	1.1 ± 1	1.4 ± 0.3	2.9 ± 1.4	11.3 ± 3.1	37.3 ± 15.3	60.1 ± 17.6	84 ± 13.3	85.8 ± 9.1
Glutamate	0.2 ± 0.2	0.1 ± 0.1	0.2 ± 0.2	0.4 ± 0.2	0.3 ± 0.1	0.5 ± 0.1	0.5 ± 0.3	0.3 ± 0.1	1.3 ± 0.3	2.5 ± 0.4	7 ± 1	12.4 ± 7.4	33.2 ± 1.2	36.9 ± 13.9
2OG	0.2 ± 0.3	0.2 ± 0.1	0.2 ± 0.2	0.3 ± 0.2	0.5 ± 0.2	0.5 ± 0.3	0.6 ± 0.6	0.4 ± 0.1	0.8 ± 0.7	1.6 ± 0.7	6.2 ± 1	11.7 ± 6	30.7 ± 4.3	33.4 ± 12.5
Succinate	0 ± 0.1	0.2 ± 0.1	0.2 ± 0.1	0.2 ± 0.1	0.3 ± 0.1	0.3 ± 0.1	0.4 ± 0.3	0.1 ± 0.1	0.8 ± 0.3	2.4 ± 1.1	8.4 ± 2.5	15.5 ± 5.8	29.9 ± 3.3	34.2 ± 10.8
Fumarate	1.1 ± 1.1	2.7 ± 2.6	4.7 ± 3.2	4.3 ± 1.7	4.7 ± 2.1	6.2 ± 2.3	10.2 ± 7.5	7.6 ± 1.5	9.1 ± 5.9	5.9 ± 1.8	23.2 ± 32	72.2 ± 17.5	70.7 ± 15.4	70.5 ± 20.1
Fumarate C4	2.3 ± 2.8	19 ± 2.6	43 ± 5	61 ± 20	86 ± 14	82 ± 19	89 ± 14	83 ± 24	90 ± 7	82 ± 16	2.3 ± 2.8	19 ± 2.6	43 ± 5	61 ± 20
Fumarate C1-3	0 ± 0	1 ± 0.9	0 ± 0	2 ± 4	6 ± 2	37 ± 8	72 ± 11	71 ± 25	83 ± 11	71 ± 16	0 ± 0	1 ± 0.9	0 ± 0	2 ± 4
Valine	0.5 ± 0.9	0.5 ± 1.1	0.2 ± 0.4	1.6 ± 1.7	0.5 ± 0.7	0.2 ± 0.5	0.4 ± 0.5	0.5 ± 1	1.1 ± 1	0.6 ± 0.8	2.1 ± 1.3	0.3 ± 0.4	0.7 ± 0.5	1.3 ± 1.2
Threonine	0.6 ± 0.3	1.1 ± 0.7	1.6 ± 1.3	1.3 ± 1.8	1.4 ± 1	0.5 ± 0.2	1.9 ± 1.9	0.8 ± 0.6	1.5 ± 1.2	0.5 ± 0.5	0.9 ± 0.5	5.9 ± 2.7	4.5 ± 2.5	7.6 ± 7.5
Glucose	2.3 ± 0.1	2.3 ± 0.1	2.3 ± 0	2.3 ± 0.1	2.3 ± 0.1	2.2 ± 0.3	3.7 ± 2.7	2.3 ± 0.1	2.4 ± 0.1	2.4 ± 0	2.4 ± 0.1	2.9 ± 0.2	4.2 ± 0.6	7.7 ± 3.4
Fructose	0.5 ± 0.6	0.2 ± 0.2	0.4 ± 0.5	0.1 ± 0.2	0.1 ± 0.1	0.2 ± 0.2	2.3 ± 3.3	0.2 ± 0.1	0.2 ± 0.3	0.1 ± 0.1	0.9 ± 0.9	2.5 ± 1.6	7.5 ± 4.6	15.7 ± 8.3
Raffinose	1.1 ± 0.2	1 ± 0.2	0.9 ± 0.1	1.1 ± 0.1	1.2 ± 0.1	1.2 ± 0.1	2.4 ± 2.1	0.9 ± 0.3	1.3 ± 0.5	1.2 ± 0.2	1.5 ± 0.2	2.1 ± 0.4	3.2 ± 0.3	5.1 ± 0.3
*myo*inositol	0.9 ± 0.1	0.8 ± 0.1	0.9 ± 0.1	0.9 ± 0	0.9 ± 0	0.9 ± 0.1	1.5 ± 1.1	0.8 ± 0.1	1 ± 0.2	0.9 ± 0.1	1 ± 0.1	1.1 ± 0.2	1.9 ± 0.3	2.7 ± 0.3

B

Metabolite	Kinetic (s) 0	10	30	60	180	600	2400	3600	7200	14400
Malate C4	3.7 ± 1.5	19 ± 3.2	47 ± 12.1	66.6 ± 22.1	75 ± 17.5	79.8 ± 21.7	90.5 ± 9.6	83.5 ± 24.3	91.6 ± 11.9	95.5 ± 5.8
Aspartate C4	4.2 ± 1.3	46.6 ± 11.4	65.7 ± 6.6	79.2 ± 4.9	88.8 ± 2.3	96.6 ± 2.2	96.9 ± 3.8	97.8 ± 1.5	98.7 ± 0.1	97.3 ± 2.3
3PGA	0.3 ± 0.3	2.4 ± 0.4	8 ± 1.2	16.9 ± 3	44.7 ± 5	73.3 ± 2.4	90.8 ± 2.1	92.1 ± 1.5	94.4 ± 0.8	93.5 ± 1.3
Triose-P	0.6 ± 0.3	2.5 ± 0.6	8.4 ± 1.2	18.6 ± 2.5	46.6 ± 4.2	74.3 ± 1.7	92.8 ± 2.2	94 ± 1.3	94.6 ± 0.5	96.5 ± 0.4
FBP	0.4 ± 0.3	2.6 ± 2.1	6.2 ± 0.4	14.9 ± 4	39.9 ± 5.2	67.9 ± 4.7	83.4 ± 7.4	88.2 ± 5	91.8 ± 0.5	89 ± 1.3
F6P	0.5 ± 0.4	2.3 ± 1.2	4.1 ± 0.5	9.2 ± 1.6	31.9 ± 5	62.8 ± 4.2	82.2 ± 4.9	84.8 ± 2.5	83.5 ± 0.9	89.1 ± 4.2
SBP	1.4 ± 1	2.2 ± 1.5	7.7 ± 3.3	13.7 ± 10	24.7 ± 11.3	42.3 ± 24.3	65.1 ± 29.9	72.4 ± 24.8	92.7 ± 1.1	73.4 ± 25.6
S7P	0.3 ± 0.1	1.4 ± 0.5	7.3 ± 0.9	16.5 ± 3.3	48.9 ± 3.9	75.1 ± 2.4	92.1 ± 1.1	92.5 ± 1.7	95 ± 1	94.3 ± 2
R5P	0.4 ± 0.4	0.8 ± 0.5	4.4 ± 1	11.4 ± 4.1	29.7 ± 3.2	56.7 ± 4.8	75.5 ± 7.1	76.5 ± 7.3	83 ± 1.4	88.3 ± 1.5
Ru5P+Xu5P	0.5 ± 0.3	1.1 ± 0.4	4.5 ± 0.6	13.8 ± 3.2	43.2 ± 5.2	72.6 ± 2.9	91.7 ± 2.7	93.6 ± 1	95 ± 0.4	95.9 ± 0.6
RuBP	0.4 ± 0.3	3.2 ± 2.8	5.3 ± 0.1	15.4 ± 3.6	42.2 ± 2.5	70.7 ± 2.5	85.7 ± 4.2	87.4 ± 7.4	91 ± 2	89.7 ± 5.3
ADPG	0.2 ± 0.3	1.9 ± 1.4	3.2 ± 1.3	7.4 ± 2.5	27.9 ± 3.4	70.6 ± 4.2	88.9 ± 5.2	90.6 ± 2.9	90.8 ± 7.1	94.9 ± 1.9
G6P	0.4 ± 0.4	2.1 ± 0.5	2.6 ± 1	5.3 ± 1	25.6 ± 3.3	56.2 ± 5.5	76.6 ± 6.8	82.5 ± 0.8	80.5 ± 3	82.1 ± 6.5
G1P	0.2 ± 0.1	0.5 ± 0.4	4 ± 0.9	6.1 ± 1.5	29.6 ± 2.3	53.2 ± 9.5	82 ± 9	87.7 ± 5	82.6 ± 13.6	68.5 ± 5.4
UDPG	0.5 ± 0.4	0.9 ± 0.8	1.5 ± 0.7	3 ± 0.9	28.9 ± 5.8	64.7 ± 8.8	84.4 ± 6.1	87 ± 8.3	74.4 ± 2.9	77.8 ± 9.6
PEP	0.5 ± 0.4	1.6 ± 0.2	4.2 ± 1.4	6.6 ± 2.7	21.7 ± 3.4	57.4 ± 3.6	87.4 ± 3.1	89.6 ± 1.1	91.7 ± 0.6	92.1 ± 3.2
Malate C1-3	0 ± 0	0 ± 0	0.4 ± 0.1	1.5 ± 0.3	12.2 ± 2.2	39.8 ± 12.7	70.8 ± 8.1	66.4 ± 22.6	79.3 ± 11.2	83.8 ± 5.4
Aspartate C1-3	0 ± 0	0.2 ± 0.2	0.7 ± 0.3	2.1 ± 0.8	14.9 ± 2.7	49.2 ± 3.6	83.5 ± 7.5	84.1 ± 5.3	89.5 ± 1.9	89.5 ± 4.1
Pyruvate	0.2 ± 0.3	0.7 ± 0.6	1.5 ± 0.4	2.9 ± 1.1	15 ± 4.2	46 ± 5.1	78.6 ± 4	77.2 ± 7.4	84.2 ± 3.9	84.4 ± 1.6
Alanine	1.2 ± 0.2	1.3 ± 0.5	1.5 ± 0.7	0.8 ± 0.3	6.2 ± 2	34.2 ± 4.7	76.1 ± 4.6	77.3 ± 5.9	84.4 ± 2.4	77.8 ± 5.2
Malate	0.9 ± 0.4	4.8 ± 0.8	12.1 ± 3.1	17.8 ± 5.6	27.9 ± 5.7	49.8 ± 14.9	75.8 ± 8.3	70.6 ± 23	82.4 ± 11.4	86.7 ± 5.5
Aspartate	1.1 ± 0.3	11.8 ± 2.8	17 ± 1.6	21.3 ± 1.8	33.4 ± 2.1	61.1 ± 2.5	86.9 ± 6.5	87.5 ± 4.3	91.8 ± 1.5	91.5 ± 3.4
2PG	0 ± 0	5.2 ± 6.3	2.4 ± 4.9	8.8 ± 5.9	50.7 ± 10.5	74.6 ± 6.3	89.7 ± 7.6	96.1 ± 4	99.6 ± 0.6	91.7 ± 1.6
Glycine	0.5 ± 1	0.5 ± 1.1	0 ± 0.1	1 ± 1.3	22.3 ± 8.1	70.2 ± 21.8	92.5 ± 12.9	92.2 ± 12.2	73.9 ± 1.3	87.8 ± 11.3
Serine	0.4 ± 0.4	0.4 ± 0.6	0.5 ± 0.6	0.2 ± 0.2	8.7 ± 3.1	52.7 ± 14.3	86 ± 8.2	87.3 ± 6.9	89.9 ± 5.5	87.1 ± 8
Glycerate	3.3 ± 0.3	3 ± 0.1	3.6 ± 0.3	3.3 ± 0.1	4 ± 0.5	9.4 ± 3.1	39.4 ± 12.3	40.1 ± 14.4	56.8 ± 3.9	72.7 ± 4.4
Glutamate	0.4 ± 0.1	0.5 ± 0.1	0.6 ± 0.2	0.4 ± 0.1	0.6 ± 0.1	1.6 ± 0.4	10.7 ± 4.6	13.6 ± 3.7	34.6 ± 0.6	47.6 ± 4.3
2OG	0.4 ± 0.1	0.4 ± 0.2	0.6 ± 0.3	0.4 ± 0.2	0.7 ± 0.2	1.4 ± 0.5	9.5 ± 3.3	12.1 ± 2.1	35.5 ± 1.8	47.9 ± 3.5
Succinate	0.4 ± 0.5	0.1 ± 0.1	0.5 ± 0.6	0.1 ± 0.3	0.8 ± 0.9	1.5 ± 1.1	13 ± 3.5	17.7 ± 5.1	30.2 ± 0.5	38.6 ± 6.4
Fumarate	0.5 ± 0.6	4.9 ± 0.6	10.2 ± 1.1	15.5 ± 6.9	26.4 ± 6	52.1 ± 10.1	80.4 ± 7.6	71.8 ± 23.9	84.5 ± 16.6	79.2 ± 14
Fumarate C4	4.6 ± 4.5	10.1 ± 8.2	17.2 ± 8.1	17.9 ± 7.2	19.2 ± 8.5	25.5 ± 9.6	38 ± 22	32 ± 6	33 ± 17	23 ± 6
Fumarate C1-3	0 ± 0	0.4 ± 0.8	0.7 ± 1.8	0 ± 0	0 ± 0	0 ± 0	2 ± 3	0 ± 0	2 ± 3	0 ± 1
Valine	0.8 ± 0.2	0.7 ± 0.1	0.7 ± 0.1	0.7 ± 0.1	1.1 ± 0.9	0.6 ± 0.1	0.9 ± 0.5	0.9 ± 0.5	0.9 ± 0.1	1 ± 0.2
Threonine	1.9 ± 0.4	1.6 ± 0.5	1.6 ± 0.1	1.5 ± 0.1	1.7 ± 0.2	1.8 ± 0.4	3.1 ± 0.9	4.5 ± 0.9	6 ± 3.1	3.6 ± 0.9
Glucose	2.8 ± 0.2	2.6 ± 0	2.8 ± 0.2	2.9 ± 0.2	2.8 ± 0.2	3 ± 0.2	3.3 ± 0.4	4.1 ± 0.9	7.7 ± 1.7	4.3 ± 0.4
Fructose	0.9 ± 0.7	0.4 ± 0.4	0.9 ± 0.4	1.1 ± 0.7	1.4 ± 1	2.1 ± 1.5	2.9 ± 1.4	6.4 ± 4.2	7.7 ± 1.9	3.4 ± 0.9
Raffinose	2.2 ± 0.4	2 ± 0.4	2.6 ± 0.2	2 ± 0.4	2.8 ± 0.4	2.9 ± 0.7	4.7 ± 2.1	7.5 ± 3.7	24.8 ± 2.3	17.1 ± 3.1
*myo*inositol	1.2 ± 0.1	1.2 ± 0.1	1.4 ± 0.3	1.3 ± 0.2	1.3 ± 0.1	1.3 ± 0.1	1.8 ± 0.2	2.4 ± 0.2	5 ± 0.4	5.3 ± 0.3

Supplemental Table S4. ^{13}C amounts for individual metabolites in medium and low light. (A) ^{13}C amounts in medium light (ML), and **(B)** in low light (LL). ^{13}C amounts were separately calculated for the C4 and C1-3 positions of malate and aspartate (for further information about calculations see Supplemental Datasets S7-8). ^{13}C amounts are expressed as natom ^{13}C equivalents g^{-1} FW. Ratio of ^{13}C amounts in the C4 position of aspartate : ^{13}C amounts in the C4 position of malate (Aspartate C4 : Malate C4) and of ^{13}C amounts in PEP : ^{13}C amounts in 3PGA (PEP : 3PGA) are also indicated. Data used for malate C4, malate C1-3, SBP, G1P, UDPG, serine, glycine, fumarate and (in ML only) glycerate were corrected for the presence of an inactive pool. Mean ± SD, n = 3 to 6 and 2 to 6 replicates in ML and LL, respectively. Data are presented as plots in Supplemental Fig. S7 and ratio Aspartate C4 : Malate C4 as plot in Fig. 5. n.d. stands for not determined. For abbreviations, see legend of Supplemental Figure S2 or Supplemental Table S1.

A

Metabolite	Kinetic (s)													
	0	5	10	15	20	30	50	60	180	300	600	1200	2400	3600
Malate C4	30.5 ± 19.9	274 ± 157.2	359 ± 76.3	548 ± 227.8	527 ± 235.7	553 ± 240	899 ± 483.2	704 ± 281.1	851 ± 363.9	1171 ± 190	1300 ± 242.5	1359 ± 335.8	1090 ± 334.6	1527 ± 0
Aspartate C4	11.7 ± 7.1	203 ± 30.1	321 ± 53.3	296 ± 23.6	342 ± 10.2	375 ± 42.4	400 ± 8.6	378 ± 11.3	424 ± 25	434 ± 12.5	442 ± 8.5	450 ± 1.4	438 ± 10.5	445 ± 13.1
3PGA	2.6 ± 2.9	87.2 ± 20	175 ± 41.4	265 ± 26.9	358 ± 46.8	546 ± 25.1	805 ± 64.9	904 ± 25	1451 ± 28	1653 ± 18.3	1805 ± 52.6	1882 ± 18.4	1914 ± 17.7	1950 ± 18.2
DHAP	6.5 ± 5.4	42.8 ± 16.6	111 ± 28.6	176 ± 28.2	242 ± 30.8	391 ± 17.3	582 ± 38	655 ± 11.3	1038 ± 15.6	1200 ± 38	1286 ± 18.9	1358 ± 16.1	1372 ± 3	1395 ± 11.4
FBP	0 ± 0.1	1.8 ± 0.5	4.2 ± 1.3	6.6 ± 0.9	9.2 ± 1.4	15.4 ± 1.7	24 ± 2.5	28 ± 1.4	46.3 ± 1.7	53.5 ± 2.4	60.1 ± 2.6	64.5 ± 0.7	64.2 ± 2.5	66.5 ± 0.8
F6P	0.9 ± 0.8	16.8 ± 5.6	40.1 ± 9.5	62.2 ± 7.2	89.9 ± 12.8	143 ± 21.6	233 ± 18.1	291 ± 13.1	518 ± 44.6	648 ± 27.6	737 ± 15.3	772 ± 9.3	771 ± 20.2	804 ± 13.5
SBPa	0.3 ± 0.3	2.4 ± 1	3.8 ± 2.5	6 ± 1.8	7.9 ± 2.1	16.9 ± 3.4	18.3 ± 3.9	22.9 ± 10	30.2 ± 14.4	33.2 ± 18.2	53.1 ± 1.5	46.3 ± 11.5	50.8 ± 5	50 ± 8.1
S7P	0.4 ± 0.6	10.8 ± 3.8	28.5 ± 13.8	58.2 ± 9.9	95.2 ± 14.9	138 ± 36	242 ± 17.2	269 ± 7.9	415 ± 16.5	476 ± 7	527 ± 12	543 ± 5.8	550 ± 3.6	553 ± 10.9
R5P	0.1 ± 0.1	0.3 ± 0.1	1.1 ± 0.5	1.1 ± 0.5	2.9 ± 0.7	3.7 ± 0.7	5.8 ± 0.7	6.5 ± 1	11.1 ± 0.6	15.2 ± 1.9	18.9 ± 0.1	19.4 ± 0.8	20.1 ± 0.6	21.1 ± 0.8
Ru5P+Xu5P	1.9 ± 1.5	15.3 ± 2.3	28.6 ± 6.5	65.5 ± 10.7	90.3 ± 19.8	138.1 ± 13	253.9 ± 30.9	300.4 ± 8	549 ± 21.4	635 ± 32.4	717.6 ± 2.4	758.8 ± 7	764.8 ± 6.2	776.7 ± 5.2
RuBP	1.1 ± 1.2	16.3 ± 4.9	32.5 ± 9.5	62.6 ± 5.9	85.1 ± 9.1	143 ± 8.7	242 ± 24.8	284 ± 16.5	481 ± 10.7	541 ± 14.2	602 ± 11.4	624 ± 11.5	636 ± 10.8	639 ± 6.1
G6P	2.7 ± 1.5	6.2 ± 4.3	23.5 ± 5.1	39.5 ± 5.8	54.6 ± 7.7	97.7 ± 21.9	193 ± 31	250 ± 20	568 ± 60.9	784 ± 87.5	905 ± 17.1	997 ± 19	1012 ± 37.1	1061 ± 30.5
G1P	0 ± 0	1.7 ± 1	5.4 ± 3.7	9.5 ± 2.3	13.9 ± 4.5	26.1 ± 7.7	35.3 ± 6.5	51.6 ± 13.4	97.6 ± 19.2	114 ± 37.4	162 ± 4.6	161 ± 17.1	147 ± 19.4	159 ± 25.6
ADPG	0 ± 0	0.1 ± 0	0.2 ± 0.1	0.5 ± 0.1	0.8 ± 0.2	1.2 ± 0.5	2.4 ± 0.7	3 ± 0.4	5.6 ± 0.5	6.8 ± 0.6	7.9 ± 0.2	8.2 ± 0.2	8.3 ± 0.3	8.4 ± 0.1
UDPG	0.8 ± 0.6	2.7 ± 1.3	2.9 ± 0.9	7.3 ± 2.2	8.8 ± 1.3	24.1 ± 3.7	61.3 ± 17.2	66 ± 7.1	177 ± 24	224 ± 26	260 ± 23.1	275 ± 25.3	285 ± 14.8	294 ± 13.7
PEP	0.6 ± 0.5	3.6 ± 1.8	4.2 ± 1.5	9.9 ± 2	14.4 ± 5.6	27.5 ± 6.3	48.3 ± 13.7	70.2 ± 6.6	280 ± 42.4	405 ± 13.7	549 ± 23.2	659 ± 18.2	711 ± 6.2	720 ± 12.1
Malate C1-3	0.2 ± 0.5	3.1 ± 6.8	0.4 ± 0.9	4 ± 1.9	19.4 ± 14.8	43.9 ± 22.8	264 ± 332.4	153 ± 60.4	796 ± 343.8	1639 ± 306	2377 ± 314.9	3090 ± 703.6	2635 ± 779	3895 ± 124
Aspartate C1-3	0 ± 0	0 ± 0	2.3 ± 3.2	1.4 ± 1.6	8.8 ± 8.8	33.1 ± 4.6	51.4 ± 14.6	69.8 ± 12.6	435 ± 118.3	648 ± 52.5	862 ± 45.7	1112 ± 32.2	1121 ± 50.1	1172 ± 90.2
Pyruvate	0 ± 0	0 ± 0	0 ± 0	0 ± 0	2.4 ± 2	15.9 ± 5.6	36.5 ± 4.9	49.1 ± 4.8	328 ± 77.2	547 ± 44.4	827 ± 28.4	1017 ± 45.5	1107 ± 17.7	1119 ± 8.2
Alanine	12.9 ± 4.4	10.7 ± 8.2	12.5 ± 2.7	17.6 ± 2.4	16.2 ± 5.5	20.2 ± 9.2	28.2 ± 5.7	28 ± 13.7	279 ± 74.2	595 ± 22.1	1015 ± 49.9	1380 ± 82.3	1519 ± 58.7	1575 ± 46.4
2PG	0 ± 0	0 ± 0	0 ± 0	0 ± 0	0.1 ± 0	0.1 ± 0.1	0.2 ± 0.1	0.2 ± 0	0.5 ± 0.1	0.6 ± 0.1	0.7 ± 0	0.7 ± 0	0.7 ± 0	0.7 ± 0
Glycine	0.6 ± 0.6	1.4 ± 1.5	0.5 ± 0.6	0.8 ± 0.7	0.3 ± 0.6	1.3 ± 0.8	3.1 ± 1.7	5.8 ± 1.1	20.4 ± 1.2	35.3 ± 3.6	48.5 ± 7.5	51.4 ± 11.6	54.9 ± 6.3	58.3 ± 4.4
Serine	0 ± 0	0 ± 0	0.4 ± 1	0 ± 0	0 ± 0	1.1 ± 1.3	0 ± 0.1	0 ± 0	20.5 ± 4.2	65.1 ± 5.8	117 ± 13.3	153 ± 19.2	158 ± 12.5	175 ± 14.6
Glycerate	6.3 ± 2	2.5 ± 0.9	3.8 ± 2	2.9 ± 3.2	2.3 ± 2.7	5.6 ± 2.2	3.8 ± 3.3	4.7 ± 0.9	9.5 ± 4.6	37.3 ± 10.2	123 ± 50.5	199 ± 58.3	278 ± 43.8	284 ± 30.1
Valine	0.4 ± 0.8	0.5 ± 1	0.2 ± 0.3	1.5 ± 1.6	0.4 ± 0.6	0.2 ± 0.4	0.3 ± 0.5	0.4 ± 0.9	1 ± 0.9	0.6 ± 0.7	1.9 ± 1.1	0.3 ± 0.3	0.6 ± 0.5	1.2 ± 1.1
Threonine	0.7 ± 0.3	1.4 ± 0.8	1.9 ± 1.5	1.6 ± 2.2	1.6 ± 1.2	0.5 ± 0.2	2.3 ± 2.3	1 ± 0.7	1.8 ± 1.4	0.6 ± 0.6	1 ± 0.6	7.1 ± 3.3	5.4 ± 3	9.1 ± 9
Glutamate	7.8 ± 9.6	6.8 ± 4.8	10 ± 8	18.6 ± 9	13.2 ± 4.6	23.9 ± 6	22.7 ± 16.3	16.3 ± 4.9	62.1 ± 13.3	119 ± 18.8	339 ± 50	598 ± 358	1601 ± 58.6	1779 ± 670
Fumaratea	0.1 ± 0.1	0.1 ± 0.1	0.2 ± 0.2	0.2 ± 0.1	0.2 ± 0.1	0.3 ± 0.1	0.5 ± 0.4	0.4 ± 0.1	0.5 ± 0.3	0.3 ± 0.1	1.2 ± 1.7	3.8 ± 0.9	3.7 ± 0.8	3.7 ± 1.1
Succinate	0.1 ± 0.1	0.4 ± 0.2	0.3 ± 0.2	0.5 ± 0.2	0.5 ± 0.1	0.7 ± 0.2	0.7 ± 0.6	0.3 ± 0.2	1.7 ± 0.6	4.8 ± 2.1	16.7 ± 5	30.8 ± 11.5	59.3 ± 6.6	67.9 ± 21.4
2OG	4.6 ± 6.8	4.2 ± 3.4	4.8 ± 5.5	7.3 ± 5.1	12.1 ± 4.8	12 ± 6.9	15.2 ± 14.1	9.6 ± 1.4	20.7 ± 16.6	39.1 ± 17	154 ± 25.1	290 ± 149	765 ± 106	831 ± 311
Glucose	142 ± 3.3	139 ± 6.5	141 ± 2.4	139 ± 5.3	140 ± 4.3	136 ± 18.3	227 ± 166	140 ± 3.6	148 ± 9	146 ± 1	147 ± 4.8	177 ± 9.8	258 ± 39.3	467 ± 206
Fructose	12.6 ± 16.2	4 ± 6.2	9.3 ± 12	3.1 ± 6.3	1.7 ± 3.4	5.9 ± 6.1	57.5 ± 84.6	3.9 ± 3.3	5.5 ± 8.2	1.8 ± 2.8	23.2 ± 21.9	63.1 ± 39.7	190 ± 116.9	398 ± 210.4
Raffinose	64.3 ± 12.3	63.3 ± 11.4	56.2 ± 5.4	67.7 ± 3.3	70.4 ± 6.4	72.8 ± 6.8	145 ± 127	55.7 ± 18.7	76.9 ± 28.9	73.3 ± 13.2	93.7 ± 9.3	127 ± 22.1	194 ± 15.6	310 ± 16.6
myoinositol	22.1 ± 2.5	19.7 ± 3.1	20.9 ± 2.9	20.6 ± 0.9	22.3 ± 0.5	21.8 ± 1.6	37.1 ± 25.4	20.6 ± 2	24.2 ± 4.7	22.9 ± 2.9	23.3 ± 3.5	27.1 ± 5	45.6 ± 6.7	65.3 ± 8.2
Aspartate C4 : Malate C4	0.65 ± 0.59	0.92 ± 0.41	0.92 ± 0.15	0.6 ± 0.21	0.79 ± 0.46	0.75 ± 0.23	0.55 ± 0.26	0.61 ± 0.24	0.56 ± 0.18	0.38 ± 0.07	0.35 ± 0.06	0.35 ± 0.12	0.43 ± 0.11	0.29 ± 0.01
PEP : 3PGA	0.72 ± 0.98	0.04 ± 0.01	0.03 ± 0.01	0.04 ± 0.01	0.04 ± 0.01	0.05 ± 0.01	0.06 ± 0.02	0.08 ± 0.01	0.19 ± 0.03	0.25 ± 0.01	0.3 ± 0.01	0.35 ± 0.01	0.37 ± 0	0.37 ± 0

**B**	Kinetic (s)									
Metabolite	0	10	30	60	180	600	2400	3600	7200	14400
Malate C4	60 ± 24.9	307 ± 51.3	759 ± 196	1076 ± 357	1211 ± 282	1289 ± 351	1462 ± 154	1349 ± 392	1480 ± 191	1543 ± 94.3
Aspartate C4	27.6 ± 8.3	306 ± 75.1	432 ± 43.6	520 ± 32.3	584 ± 15.4	635 ± 14.4	637 ± 25	643 ± 10	649 ± 0.7	640 ± 14.9
3PGA	4.8 ± 5	41.8 ± 6.1	137 ± 21	290 ± 52.2	766 ± 84.9	1255 ± 40.5	1556 ± 35.5	1578 ± 26.1	1617 ± 13.8	1602 ± 21.6
Triose-P	7.1 ± 3.3	28.1 ± 7.2	94 ± 13	208 ± 28.2	522 ± 47.1	833 ± 19.5	1040 ± 24.4	1053 ± 14.6	1061 ± 5.9	1081 ± 4.5
FBP	0.2 ± 0.2	1.4 ± 1.1	3.3 ± 0.2	7.9 ± 2.1	21.2 ± 2.7	36 ± 2.5	44.3 ± 3.9	46.8 ± 2.7	48.7 ± 0.3	47.2 ± 0.7
F6P	3.9 ± 3.2	17.7 ± 9.1	30.7 ± 4	69.4 ± 11.8	242 ± 38.1	476 ± 31.6	623 ± 37.3	643 ± 19.2	633 ± 6.6	675 ± 31.5
SBP	0.5 ± 0.3	0.7 ± 0.5	2.5 ± 1.1	4.4 ± 3.2	7.9 ± 3.7	13.6 ± 7.8	21 ± 9.6	23.3 ± 8	29.9 ± 0.3	23.7 ± 8.2
S7P	0.2 ± 0.4	6.7 ± 2.8	15.5 ± 10.1	27.2 ± 12.4	52.9 ± 10.2	85.2 ± 21.2	161 ± 13.1	232 ± 47.2	272 ± 8.4	305 ± 4.5
R5P	0.1 ± 0.1	0.2 ± 0.1	1.1 ± 0.2	2.8 ± 1	7.4 ± 0.8	14.1 ± 1.2	18.8 ± 1.8	19.1 ± 1.8	20.7 ± 0.4	22 ± 0.4
Ru5P+Xu5P	6.2 ± 4	13.4 ± 4.9	53.4 ± 6.7	163 ± 37.9	508 ± 60.7	853 ± 33.7	1078 ± 32.1	1100 ± 11.2	1116 ± 4.5	1126 ± 6.9
RuBP	0.6 ± 0.7	9.3 ± 3.2	16.9 ± 4.9	29.6 ± 11.5	45.1 ± 8	80.6 ± 13.3	156 ± 19.7	246 ± 63.3	294 ± 0.3	325 ± 8.5
ADPG	5 ± 4.5	26.5 ± 5.8	32.5 ± 13	67.3 ± 12	323 ± 41.5	709 ± 69.5	966 ± 85.6	1040 ± 10.3	1015 ± 37.6	1036 ± 81.7
G6P	0.3 ± 0.3	0.9 ± 0.7	7.3 ± 1.7	11.1 ± 2.7	54.2 ± 4.2	97.5 ± 17.4	150 ± 16.5	161 ± 9.1	151 ± 24.9	126 ± 10
G1P	0 ± 0	0.1 ± 0.1	0.1 ± 0.1	0.3 ± 0.1	1.2 ± 0.1	3.1 ± 0.2	3.9 ± 0.2	4 ± 0.1	4 ± 0.3	4.1 ± 0.1
UDPG	2.4 ± 2	4.3 ± 3.7	7.1 ± 3.4	14 ± 4.4	136 ± 27.4	304 ± 41.4	397 ± 28.6	409 ± 39.2	349 ± 13.8	365 ± 45
PEP	3.2 ± 2.4	10.8 ± 1.1	28 ± 9.5	44.8 ± 18.3	146 ± 23.2	387 ± 24.6	589 ± 21.1	604 ± 7.6	618 ± 4.2	621 ± 21.5
Malate C1-3	0 ± 0	0.6 ± 1.2	20.2 ± 2.9	71.1 ± 16.8	593 ± 108	1930 ± 615	3432 ± 391	3215 ± 1095	3844 ± 545	4060 ± 262
Aspartate C1-3	0 ± 0	4 ± 4.1	14.3 ± 6.1	40.5 ± 15.4	295 ± 54.2	971 ± 71.3	1647 ± 147.3	1659 ± 103.9	1766 ± 37.4	1766 ± 81.2
Pyruvate	1.4 ± 2.1	4.9 ± 4.2	10 ± 2.4	19.1 ± 7.1	101 ± 28.1	308 ± 34.3	526 ± 27.1	516 ± 49.4	563 ± 26.2	565 ± 10.4
Alanine	12.7 ± 1.9	14.2 ± 5	15.8 ± 7.8	9 ± 2.7	66.1 ± 21.9	366 ± 50	814 ± 48.8	827 ± 63.5	903 ± 25.3	832 ± 56
2PG	0 ± 0	0 ± 0	0 ± 0	0.1 ± 0	0.4 ± 0.1	0.6 ± 0	0.7 ± 0.1	0.7 ± 0	0.8 ± 0	0.7 ± 0
Glycine	0.4 ± 0.8	0.5 ± 0.9	0 ± 0.1	0.8 ± 1.1	19.1 ± 7	60.3 ± 18.7	79.5 ± 11.1	79.2 ± 10.5	63.5 ± 1.1	75.5 ± 9.7
Serine	1.7 ± 1.8	1.6 ± 2.3	2 ± 2.4	0.6 ± 0.9	34.6 ± 12.1	209 ± 56.8	341 ± 32.4	346 ± 27.2	356 ± 21.6	345 ± 31.7
Glycerate	71 ± 7.4	64.2 ± 2.6	77.5 ± 6	69.4 ± 1.7	84.8 ± 10.4	199 ± 65.5	839 ± 262	854 ± 307	1209 ± 84.1	1548 ± 93.8
Glutamate	1.8 ± 0.4	1.6 ± 0.3	1.6 ± 0.3	1.6 ± 0.2	2.6 ± 2	1.4 ± 0.3	2.1 ± 1.1	2 ± 1	2 ± 0.1	2.2 ± 0.4
2OG	4.4 ± 0.9	3.8 ± 1.2	3.7 ± 0.2	3.5 ± 0.3	3.9 ± 0.5	4.3 ± 0.9	7.2 ± 2.2	10.5 ± 2	14 ± 7.4	8.4 ± 2.1
Succinate	24.7 ± 4.4	36.8 ± 9.9	41.7 ± 11.7	28.5 ± 6	43.5 ± 10.2	113 ± 30	753 ± 325	959 ± 262	2441 ± 43.1	3361 ± 300.9
Fumarate	0 ± 0	0.3 ± 0	0.6 ± 0.1	1 ± 0.4	1.7 ± 0.4	3.3 ± 0.6	5.1 ± 0.5	4.5 ± 1.5	5.3 ± 1	5 ± 0.9
Valine	0.8 ± 0.9	0.1 ± 0.2	0.9 ± 1.2	0.3 ± 0.5	1.4 ± 1.7	2.8 ± 2	24.1 ± 6.6	32.9 ± 9.4	56 ± 0.9	71.6 ± 11.8
Threonine	20.2 ± 4.8	17.3 ± 10	27.1 ± 11.5	16.9 ± 8.5	32.3 ± 11.2	64.8 ± 21.6	432 ± 150	549 ± 94.5	1612 ± 82.8	2180 ± 160.3
Glucose	221 ± 18.1	207 ± 2.7	221 ± 16.8	226 ± 14.7	221 ± 15.1	238 ± 16.9	263 ± 28	326 ± 69.1	607 ± 133.7	341 ± 33.2
Fructose	23.4 ± 17.9	10 ± 10.2	21.8 ± 10.8	28.8 ± 18.6	35 ± 26.5	54.8 ± 38.5	73.3 ± 35	163.7 ± 107.2	196.4 ± 48.7	87.2 ± 23.3
Raffinose	48.3 ± 9.3	44.7 ± 6.6	46.7 ± 8.6	46.8 ± 6.5	53.1 ± 4.6	53.4 ± 5.3	82.5 ± 84.3	58 ± 21.2	53.5 ± 10.2	55.1 ± 9.9
*myo*inositol	43.9 ± 2.2	41.4 ± 4.5	50.3 ± 9.5	47 ± 5.6	46.8 ± 3.9	45.3 ± 3	64.4 ± 6	87.9 ± 8	179 ± 14.3	189 ± 12.3
Aspartate C4 : Malate C4	n.d	1.05 ± 0.45	0.6 ± 0.16	0.54 ± 0.22	0.5 ± 0.1	0.53 ± 0.17	0.44 ± 0.05	0.52 ± 0.2	0.44 ± 0.06	0.42 ± 0.02
PEP : 3PGA	n.d.	0.27 ± 0.04	0.2 ± 0.05	0.15 ± 0.04	0.19 ± 0.01	0.31 ± 0.01	0.38 ± 0	0.38 ± 0.01	0.38 ± 0.01	0.39 ± 0.01

Supplemental Table S5. Summed ^{13}C amounts in the C4 and C1-3 positions of malate and aspartate, and summed ^{13}C amounts in other sets of metabolites in in medium and low light. **(A)** Summed ^{13}C amounts in medium light (ML), and **(B)** in low light (LL). C carriers in CO_2 shuttle corresponds to C1-3 positions of malate and aspartate, PEP, pyruvate and alanine. ^{13}C amounts are expressed as natom ^{13}C equivalents g^{-1} FW. Mean ± SD, n = 3 to 6 and 2 to 6 replicates in ML and LL, respectively. Data used for malate C4, malate C1-3, SBP, serine, glycine and (in ML only) glycerate were corrected for the presence of an inactive pool. Data are presented as plots in Fig. 3, and single data are presented in Supplemental Dataset S10. For abbreviations, see legend of Supplemental Figure S2 or Supplemental Table S1.

**A**	Kinetic (s)													
Metabolic sector	0	5	10	15	20	30	50	60	180	300	600	1200	2400	3600
C4 positions of malate and aspartate	42.1 ± 18.2	476 ± 155	626 ± 167	844 ± 237	868 ± 243	928 ± 265	1299 ± 489	1082 ± 282	1275 ± 368	1605 ± 191	1742 ± 250	1809 ± 336	1528 ± 344	1972 ± 13.1
C1-3 positions of malate and aspartate	0.2 ± 0.5	3.1 ± 6.8	2.3 ± 2.8	5.3 ± 1.6	28.3 ± 22.1	77 ± 24.4	315 ± 347	223 ± 62	1231 ± 379	2287 ± 303	3239 ± 314	4201 ± 679	3756 ± 821	5066 ± 211
PEP, pyruvate and alanine	13.5 ± 4.7	14.3 ± 7.4	16.7 ± 4.1	27.5 ± 3.9	33 ± 4.3	63.6 ± 11.8	113 ± 23.1	147 ± 11.9	888 ± 188	1548 ± 76.7	2391 ± 101	3056 ± 144	3337 ± 78.3	3414 ± 64.3
Carriers in CO_2 shuttle	13.7 ± 5.1	17.4 ± 8.4	18.9 ± 4	32.8 ± 4.4	61.3 ± 25.1	141 ± 27.6	428 ± 368	371 ± 53.4	2118 ± 518	3835 ± 372	5630 ± 233	7257 ± 592	7093 ± 805	8481 ± 264
3PGA and triose-P	9.1 ± 5.5	130 ± 35.2	285 ± 68.8	440 ± 54.5	599 ± 72	937 ± 40.2	1388 ± 102	1559 ± 16.7	2490 ± 41.6	2853 ± 36.2	3091 ± 65.7	3240 ± 25.2	3285 ± 20.7	3344 ± 25.1
CBC except 3PGA and triose-P	4.7 ± 1.3	63.6 ± 14	139 ± 38.7	262 ± 20.7	380 ± 54.6	599 ± 83.2	1019 ± 87.5	1202 ± 30.6	2050 ± 55.9	2402 ± 66.9	2716 ± 44.3	2829 ± 26.5	2857 ± 43.5	2910 ± 32.5
All CBC intermediates	13.8 ± 4.6	194 ± 49	424 ± 107	702 ± 74.7	980 ± 124	1536 ± 122	2407 ± 188	2761 ± 46.6	4540 ± 86.8	5254 ± 96.1	5806 ± 110	6069 ± 46.2	6142 ± 62.9	6254 ± 54.9
Starch and sucrose synthesis intermediates	3.5 ± 1.6	10.7 ± 4.3	32 ± 6.8	56.9 ± 9.7	78.2 ± 12.3	149 ± 22.6	292 ± 45.5	371 ± 35.5	848 ± 74.7	1128 ± 80.9	1335 ± 34.2	1441 ± 43	1452 ± 65	1522 ± 33.2
Photorespiratory intermediates	6.9 ± 2	4 ± 1.7	4.7 ± 1.7	3.7 ± 3.2	2.7 ± 3.1	8 ± 1.6	6.2 ± 2.7	10.7 ± 1.2	51 ± 8.2	138 ± 13.2	289 ± 59.7	403 ± 81.1	491 ± 29.3	517 ± 34.1
All measured metabolites	94 ± 37	700 ± 174	1110 ± 249	1659 ± 291	2012 ± 290	2796 ± 259	4700 ± 1293	4603 ± 322	8934 ± 869	12129 ± 409	15363 ± 372	18063 ± 507	19587 ± 1140	22439 ± 491

**B**	Kinetic (s)									
Metabolic sector	0	10	30	60	180	600	2400	3600	7200	14400
C4 positions of malate and aspartate	87.6 ± 29.4	613 ± 43	1190 ± 177	1597 ± 340	1794 ± 281	1924 ± 340	1993 ± 312	1992 ± 399	2128 ± 192	2183 ± 107
C1-3 positions of malate and aspartate	0 ± 0	4.6 ± 5.2	34.6 ± 8.7	112 ± 26.5	888 ± 137	2900 ± 675	4804 ± 921	4874 ± 1186	5609 ± 582	5826 ± 286
PEP, pyruvate and alanine	17.3 ± 4.6	27.2 ± 12.7	53.8 ± 10.8	72.9 ± 24.5	313 ± 71.2	1060 ± 101	1831 ± 232	1947 ± 112	2084 ± 47.3	2017 ± 79.2
Carriers in CO_2 shuttle	17.3 ± 4.6	31.8 ± 15	88.4 ± 19.5	184 ± 46.8	1201 ± 199	3960 ± 761	6635 ± 910	6821 ± 1297	7694 ± 629	7843 ± 332
3PGA and triose-P	11.9 ± 6.4	70 ± 6.1	230 ± 34	498 ± 77.1	1288 ± 130	2088 ± 59.3	2596 ± 58.7	2631 ± 34	2677 ± 19.7	2684 ± 17.2
CBC except 3PGA and triose-P	11.8 ± 7.4	49.5 ± 9	124 ± 22.3	304 ± 45.4	884 ± 110	1559 ± 58.6	2098 ± 53.1	2306 ± 126	2414 ± 2	2524 ± 30.7
All CBC intermediates	23.7 ± 12.1	119 ± 7.4	354 ± 55.9	802 ± 119	2172 ± 238	3647 ± 113.8	4694 ± 104	4937 ± 157	5092 ± 21.7	5208 ± 45.3
Starch and sucrose synthesis intermediates	7.7 ± 6	31.7 ± 9.8	47 ± 15.6	93 ± 14.6	515 ± 69.1	1114 ± 122	1517 ± 112	1613 ± 50.8	1519 ± 48.9	1531 ± 34.8
Photorespiratory intermediates	73.1 ± 8.5	66.3 ± 4.6	80 ± 8.5	71 ± 2.6	139 ± 24.9	469 ± 67.1	1260 ± 253	1279 ± 283	1629 ± 63.6	1969 ± 117
All measured metabolites	261 ± 106	889 ± 23	1839 ± 189	2811 ± 308	5926 ± 581	11358 ± 1287	17469 ± 1026	18499 ± 2330	22891 ± 526	24698 ± 691

Supplemental Table S6. Estimation of fluxes based on leaf area through PEPC, the C4 positions of malate and aspartate, Rubisco, the C carriers in CO_2 shuttle, and photorespiration in medium and low light. This Table is Supplemental to Table 1. Estimated fluxes are presented on a leaf area basis using the leaf area factor of 0.005484 m^2 g^{-1}. ML and LL stand for medium light and low light, respectively. For additional abbreviations, see legend of Supplemental Figure S2 or Supplemental Table S1.

		ML		LL	
Parameter	Unit	Value	time points used	Value	time points used
**Rate of photosynthesis (gas exchange)**	nmol CO_2 m^{-2} s^{-1}	**22.4 ± 1.5**		**8.3 ± 0.7**	
**Estimated flux at PEPC** (Rate of ^{13}C accumulation in all measured intermediates)	nmol ^{13}C m^{-2} s^{-1}	22.12	0-5s	11.45	0-10s
Rate of ^{13}C accumulation in C4 positions of malate and aspartate	nmol ^{13}C m^{-2} s^{-1}	15.83	0-5s	9.59	0-10s
Rate of ^{13}C accumulation in all measured intermediates except C4 positions of malate and aspartate	nmol ^{13}C m^{-2} s^{-1}	6.29	0-5s	1.86	0-10s
**Rate of ^{13}C accumulation in C4 position of malate**	nmol ^{13}C m^{-2} s^{-1}	**8.86**	0-5s	**4.50**	0-10s
**Rate of ^{13}C accumulation in C4 position of aspartate**	nmol ^{13}C m^{-2} s^{-1}	**6.97**	0-5s	**5.09**	0-10s
Rate of ^{13}C accumulation in all measured intermediates except C4 positions of malate and aspartate	nmol ^{13}C m^{-2} s^{-1}	11.29	0-30s	4.08	0-180s
Estimated movement of ^{13}C to starch and sucrose	nmol ^{13}C m^{-2} s^{-1}	0.53	0-30s	0.51	0-180s
Total estimated ^{13}C accumulation	nmol ^{13}C m^{-2} s^{-1}	11.82	0-30s	4.59	0-180s
Average ^{13}C enrichment in C4 positions of malate and aspartate (%)	%	31.5-71	0-30s	65.1-80.5	0-180s
**Estimated flux at Rubisco**	nmol C m^{-2} s^{-1}	**16.64-37.52**	0-30s	**5.07-7.05**	0-180s
Rate of ^{13}C accumulation in C1-3 positions of malate and aspartate plus pyruvate, alanine and PEP	nmol ^{13}C m^{-2} s^{-1}	2.46	15-300s	1.26	30-600s
Average ^{13}C enrichment of 3PGA (%)	%	60.2	15-300s	50.7	30-600s
**Estimated flux between CBC and CCM**	nmol C m^{-2} s^{-1}	**4.09**	15-300s	**2.48**	30-600s
Rate of ^{13}C accumulation in alanine	nmol ^{13}C m^{-2} s^{-1}	0.36	15-300s	0.12	30-600s
Rate of ^{13}C accumulation in pyruvate	nmol ^{13}C m^{-2} s^{-1}	0.36	15-300s	0.09	30-600s
Rate of ^{13}C accumulation in photorespiratory intermediates	nmol ^{13}C m^{-2} s^{-1}	0.09	50-600s	0.14	60-600s
Estimated ^{13}C released by glycine decarboxylase	nmol ^{13}C m^{-2} s^{-1}	0.02	50-600s	0.03	60-600s
Estimated ^{13}C entering photorespiration	nmol ^{13}C m^{-2} s^{-1}	0.11	50-600s	0.17	60-600s
Average ^{13}C enrichment in RuBP	%	70.1	50-600s	50.3	60-600s
**C entering photorespiration**	nmol CO_2 m^{-2} s^{-1}	**0.16**	50-600s	**1.34**	60-600s
**Rate of RuBP oxygenation**	nmol O_2 m^{-2} s^{-1}	**0.08**	50-600s	**0.17**	60-600s
**Relative rates of oxygenation and carboxylation**	ratio	**0.004**	50-600s	**0.02**	60-600s
